# Trajectories of depression and anxiety symptom severity during psychological therapy for common mental health problems

**DOI:** 10.1017/S0033291722003403

**Published:** 2023-10

**Authors:** Megan Skelton, Ewan Carr, Joshua E. J. Buckman, Molly R. Davies, Kimberley A. Goldsmith, Colette R. Hirsch, Alicia J. Peel, Christopher Rayner, Katharine A. Rimes, Rob Saunders, Janet Wingrove, Gerome Breen, Thalia C. Eley

**Affiliations:** 1 Institute of Psychiatry, Psychology and Neuroscience, King's College London, London, UK; 2 National Institute for Health and Care Research (NIHR) Maudsley Biomedical Research Centre, South London and Maudsley NHS Foundation Trust, London, UK; 3 Research Department of Clinical, Centre for Outcomes Research and Effectiveness (CORE), Educational and Health Psychology, University College London, London, UK; 4 iCope – Camden and Islington Psychological Therapies Services, Camden and Islington NHS Foundation Trust, London, UK; 5 South London and Maudsley NHS Foundation Trust, London, UK; 6 Talking Therapies Southwark, South London and Maudsley NHS Foundation Trust, London, UK

**Keywords:** cognitive-behavioural therapy, counselling, dose-response, internalising, person-centred, psychotherapy, structural equation modelling, symptom change, treatment response

## Abstract

**Background:**

There is substantial variation in patient symptoms following psychological therapy for depression and anxiety. However, reliance on endpoint outcomes ignores additional interindividual variation during therapy. Knowing a patient's likely symptom trajectories could guide clinical decisions. We aimed to identify latent classes of patients with similar symptom trajectories over the course of psychological therapy and explore associations between baseline variables and trajectory class.

**Methods:**

Patients received high-intensity psychological treatment for common mental health problems at National Health Service Improving Access to Psychological Therapies services in South London (*N* = 16 258). To identify trajectories, we performed growth mixture modelling of depression and anxiety symptoms over 11 sessions. We then ran multinomial regressions to identify baseline variables associated with trajectory class membership.

**Results:**

Trajectories of depression and anxiety symptoms were highly similar and best modelled by four classes. Three classes started with moderate-severe symptoms and showed (1) no change, (2) gradual improvement, and (3) fast improvement. A final class (4) showed initially mild symptoms and minimal improvement. Within the moderate-severe baseline symptom classes, patients in the two showing improvement as opposed to no change tended not to be prescribed psychotropic medication or report a disability and were in employment. Patients showing fast improvement additionally reported lower baseline functional impairment on average.

**Conclusions:**

Multiple trajectory classes of depression and anxiety symptoms were associated with baseline characteristics. Identifying the most likely trajectory for a patient at the start of treatment could inform decisions about the suitability and continuation of therapy, ultimately improving patient outcomes.

## Background

Routinely collected patient information can be used to explain some of the variability in outcomes following psychological therapies for depression and anxiety (Delgadillo, Moreea, & Lutz, 2016; Goddard, Wingrove, & Moran, 2015; Kessler et al., 2017; Saunders, Buckman, & Pilling, 2020a). Most studies to date have focused on outcomes measured at the end of treatment, such as remission or recovery. Although clinically informative, reliance on endpoint outcomes may obscure interindividual differences in trajectories of symptoms occurring *during* treatment. Subgroups of patients may follow distinct trajectories, such as initially slow responders who nevertheless show clinically significant improvement by the end of treatment. Without a good understanding of these trajectories, treatments might be ended or altered early because patients, or their clinicians, consider the treatment unsuitable.

Person-centered structural equation modelling techniques can be used to reveal unobserved classes (groups or clusters) of individuals who exhibit similar longitudinal trajectories, which differ to those of the other classes. It is then possible to explore how baseline variables are associated with trajectory class membership. Relevant variables could be inspected at the start of treatment to identify a patient's most likely pattern of symptoms throughout therapy, and therefore inform patient and clinician expectations and decisions. Such information could also help monitor whether patients are doing less well than expected and thereby increase positive outcomes (de Jong et al., 2021; Delgadillo et al., 2018; Lambert, 2007). Studies that have employed these methods with psychological treatment data include analyses of symptoms of depression (Lutz, Stulz, & Köck, 2009), panic disorder (Lutz et al., 2014) and post-traumatic stress disorder (PTSD; Dewar, Paradis, & Fortin, 2020), which each reported between three and five trajectory classes and some associations with baseline variables such as comorbid anxiety and depression symptoms. However, most existing analyses are arguably underpowered to reliably identify trajectory classes and associations, with samples of fewer than 500 participants. One study investigated symptom trajectories at two North London National Health Service (NHS) Improving Access to Psychological Therapies (IAPT) sites, in a larger sample of 4394 patients (Saunders et al., 2019). As part of the NHS in England, the IAPT services provide psychological therapy for adults experiencing common mental health problems such as depression and anxiety. A stepped-care framework is followed, offering evidence based low- and high-intensity treatments that primarily differ in terms of therapist involvement and number of sessions. The North London study analysed data from patients who received high-intensity therapy between 2008 and 2013 (Saunders et al., 2019). Four classes of depression symptom trajectories were identified, and five classes of anxiety symptom trajectories. Compared with trajectory classes showing improvement, classes with initially moderate-to-severe symptoms and limited change over time were associated with higher baseline levels of functional impairment, depression, anxiety, and phobia symptoms. No associations were found with age, gender, ethnicity, or prescribed psychotropic medication. In the present study, we extended these findings in a larger and more ethnically diverse sample of IAPT patients and considered additional baseline variables to test for association. We also used more recent data which is likely of higher quality due to improvements in recording patient information in IAPT (HSCIC, 2014; Saunders et al., 2020b).

## Method

### Sample

**Fig. 1.**
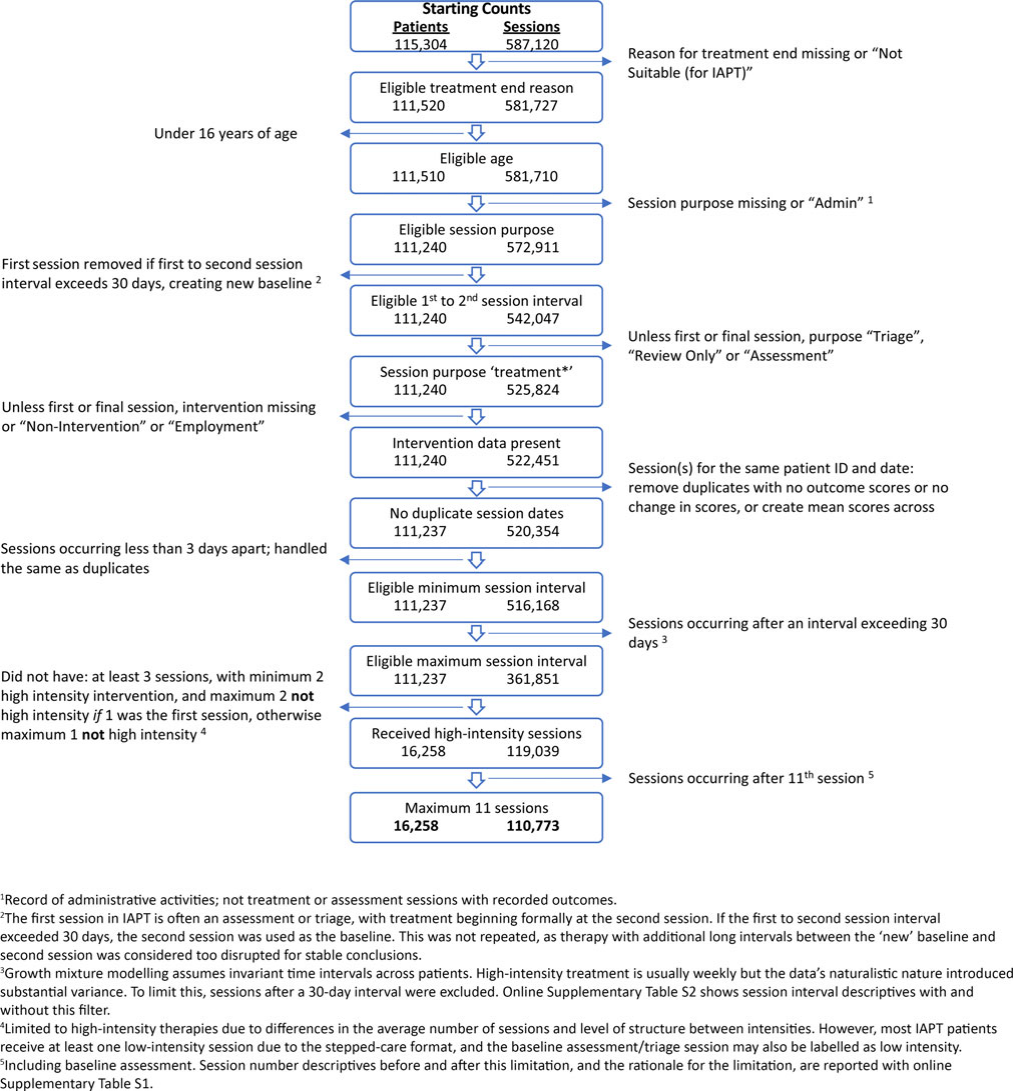
Flowchart detailing the exclusions applied to retain patient data suitable for analysis.

The sample came from anonymised patient records of routinely collected data from the four IAPT services of the South London and Maudsley (SLaM) NHS Foundation Trust. Using the Clinical Record Interactive Search (Stewart et al., 2009), we extracted treatment records of patients who had started and ended psychological therapy for symptoms of depression or anxiety between July 2014 and September 2020, irrespective of the reason for ending. From the 115 304 patients covering 587 120 sessions extracted, we identified a high-intensity treatment sample of 16 258 patients and 110 773 sessions suitable for analysis (see Fig. 1 and online Supplementary Information S1). IAPT high-intensity therapies are formal psychological treatments consistent with National Institute for Health and Care Excellence guidelines (NICE, 2011) and include disorder-specific cognitive-behavioral therapy (CBT; Roth and Pilling, 2008), counselling for depression, and other types such as interpersonal therapy (see the IAPT manual for details of therapy protocols and techniques; NCCMH, 2021). We modelled up to 11 sessions, including a baseline assessment, to ensure sufficient complete data across time points for the analysis and as modelling more sessions than a substantial proportion of patients had data for (see online Supplementary Table S1) could make optimal solutions invalid and unstable (Lutz et al., 2005). To be representative of patients receiving treatment, we did not exclude patients scoring below clinical thresholds on depression and anxiety symptom scales.

### Measures

Outcomes were self-report questionnaires administered at each session. Current symptoms of depression and anxiety were measured respectively by the Patient Health Questionnaire 9-item version (PHQ9) (Kroenke, Spitzer, & Williams, 2001; range 0-27; case threshold in IAPT ⩾10) and the Generalised Anxiety Disorder 7-item scale (GAD7) (Spitzer, Kroenke, Williams, & Löwe, 2006; range 0-21; IAPT case threshold ⩾8). Reliable improvement within trajectories was defined as a decrease of six or more points on the PHQ9, and at least four on the GAD7 (Gyani, Shafran, Layard, & Clark, 2013; NCCMH, 2021). We selected baseline self-reported patient characteristics to investigate for associations with trajectory class membership based on the available data and previously reported associations with treatment outcomes (Buckman et al., 2022; Delgadillo et al., 2016; Delgadillo, Dawson, Gilbody, & Böhnke, 2017a; Robinson, Kellett, & Delgadillo, 2020b; Saunders et al., 2019). These were: total scores from each of the PHQ9 and GAD7 (when not the outcome modelled), Work and Social Adjustment Scale (WSAS; functional impairment measure; range 0–40; Marks, 1986; Mundt, Marks, Shear, & Greist, 2002), age (in 10 year blocks), gender (female, male), ethnicity (White, Black, Asian, Mixed, other), employment (employed, unemployed, non-worker (e.g. retired, student)), psychotropic medication (not prescribed, prescribed) and disability (no, yes). In IAPT, ‘problem descriptors’ are used to indicate the disorder that is the agreed focus of treatment, in-line with NICE guidance (NICE, 2011). Problem descriptors are based on ICD-10 diagnostic codes, but their use does not mean that a patient necessarily met all diagnostic criteria for the disorder or that they would not meet criteria for other disorders. As such, comorbidity of disorders was unknown. Problem descriptor categories were depression, generalised anxiety disorder (GAD), PTSD, adjustment disorder, obsessive-compulsive disorder (OCD), mixed anxiety and depressive disorder, panic/phobia (panic disorder, agoraphobia, social phobia, specific phobia), and ‘other’ which included infrequent descriptors such as somatoform disorder. Despite showing strong associations in an existing study (Saunders et al., 2019), phobia scales were not included due to high, potentially non-random, missingness.

### Statistical analyses

#### Trajectory class models

We estimated separate growth mixture models (GMMs) for symptoms of depression and anxiety. For greater detail of the procedure, including all model selection criteria, see online Supplementary Information S2. To first determine the single trajectory form which best fit the observed data overall we compared linear (Bone, Delgadillo, & Barkham, 2021), quadratic (Phelps et al., 2018; Saunders et al., 2019) and negative log-linear (base 10) (Lutz et al., 2014) trajectories (‘latent growth curves’). We then modelled up to six latent trajectory classes using GMM, each with the best-fitting form we had identified, to determine the optimal number of classes. This revealed whether the data was better explained by multiple latent growth curves than the single average one, indicating clusters of patients with similar trajectories rather than one group with homogeneous symptom change. The upper limit of six classes was informed by past studies of trajectories during psychological therapy, which suggest between three and five classes (Dewar et al., 2020; Lutz et al., 2009; 2014; Saunders et al., 2019), thus the unlikely six class model acted as a ceiling to test against. We compared each model to a model with one fewer class using several fit indices, with a preference for the Bayesian information criterion (BIC; online Supplementary Information S2). The BIC was interpreted with the aid of an elbow plot to identify the point of diminishing gains from the addition of an extra class. We favoured models that were interpretable in terms of clinical utility as well as past literature and theory. For statistical stability, we preferred models where each class contained more than 1% of the sample (Jung & Wickrama, 2008). We described patients in each class and the overlap between depression and anxiety classes.

We followed recommendations (Jung & Wickrama, 2008; Muthén & Muthén, 2000; van de Schoot, Sijbrandij, Winter, Depaoli, & Vermunt, 2017) to first perform a version of GMM with zero variance (latent class growth analysis; LCGA). This is a simpler, less computationally demanding model that makes the often-unrealistic assumption that all individuals in a class follow exactly the same trajectory. This can result in trajectory classes that differ only in terms of starting score. We compared this simpler model to a GMM where the variance in each intercept was freed such that it could have a non-zero value but was constrained to be equal between classes, whilst the slope variance was fixed at zero. This allowed differences to be revealed in the patterns of change over time (slope of each class) rather than initial symptom levels (intercept of each class). This specification has successfully been used in previous symptom based GMMs (e.g. Lutz et al., 2014). Latent growth curve and LCGA results were similar between IAPT service-specific models (online Supplementary Figure S1) therefore analyses were performed across all four IAPT services. We used Mplus version 8.3 (Muthén & Muthén, 2017), alongside R version 3.6.3 (R Core Team, 2020) and the MplusAutomation package (Hallquist & Wiley, 2018).

#### Missing data

Missing outcome data was handled using full information maximum likelihood with robust standard errors (Mplus option ‘MLR’) due to non-normal distributions of the outcomes (see online Supplementary Figure S2). If a patient had no observed data for the PHQ9 or GAD7 they were omitted from the model of that outcome. To determine support for generalisability of the results, patients included in the analytical sample were compared to excluded patients using suitable group difference tests. The large sample size could result in statistical significance of negligible differences, therefore we primarily considered effect sizes.

#### Associations of baseline variables with trajectory class

Associations between variables measured at the start of treatment and trajectory class membership were tested using multinomial regressions. To handle missing data in the baseline variables, we ran multiple imputation using the ‘mice’ package in R, specifying 20 imputed datasets and 20 iterations (van Buuren & Groothuis-Oudshoorn, 2011). Explorations of missing and complete data between variables did now show any association patterns indicating non-random missingness. Patients were allocated to the trajectory class for which they had the highest probability of membership, and this ‘most likely’ class was the regression outcome. IAPT service was included as a covariate. We assessed statistical significance using a Bonferroni adjusted *p* value threshold of *p* < 0.025 to account for the two independent models.

## Results

### Sample descriptives

Descriptives for the sample of 16 258 patients are in Table 1. The majority had received at least one session of CBT (50.1%) or counselling (49.1%); a small proportion received other treatments (6.1%) such as interpersonal therapy. The average number of sessions received, after limiting to a maximum of 11 including the baseline assessment, was 6.81 (s.d. = 2.67), with 11.6% receiving only the minimum 3 sessions and 18.4% receiving 11 (online Supplementary Table S1). At each session, over 99% of the patients remaining in treatment had complete PHQ9 and GAD7 scores (online Supplementary Table S3). Patients in the analytical sample were similar to excluded patients (online Supplementary Table S4), besides differences that reflected inclusion criteria (e.g. number of sessions).

**Table 1.** Baseline descriptives of patients who received high-intensity psychological therapy for symptoms of depression and anxiety (*N* = 16 258)

Variable	Mean (s.d.; range) or Count (Proportion %)
Age (years)	37.55 (13.36; 16–94)
Gender	
Female	10 979 (67.5%)
Male	5262 (32.4%)
Missing	17 (0.1%)
Depression symptoms (PHQ9)	13.98 (6.39; 0–27)
Missing	153 (0.9%)
Anxiety symptoms (GAD7)	12.53 (5.39; 0–21)
Missing	154 (1.0%)
Case on PHQ9 and/or GAD7^a^	
Yes	13 735 (84.5%)
No	2368 (14.6%)
Missing	155 (1%)
Case on PHQ9^a^	
Yes	11 873 (73.0%)
No	4232 (26.0%)
Missing	153 (0.9%)
Case on GAD7^a^	
Yes	12 760 (78.5%)
No	3344 (20.6%)
Missing	154 (1.0%)
Functional impairment score (WSAS)	17.58 (9.31; 0–40)
Missing	5447 (33.5%)
Problem descriptor^b^	
Depression	6703 (41.2%)
Other	1423 (8.8%)
GAD	1393 (8.6%)
Adjustment disorder	1320 (8.1%)
PTSD	1132 (7.0%)
MADD	1129 (6.9%)
Panic/phobia	1003 (6.2%)
OCD	550 (3.4%)
Missing	1605 (9.9%)
Psychotropic medication	
Prescribed	5545 (34.1%)
Not prescribed	9977 (61.4%)
Missing	736 (4.5%)
Ethnicity	
White	9789 (60.2%)
Black	2964 (18.2%)
Mixed	1111 (6.8%)
Asian	961 (5.9%)
Other	557 (3.4%)
Missing	876 (5.4%)
Employment status	
Employed	10 033 (61.7%)
Unemployed	3572 (22.0%)
Non-worker^c^	2222 (13.7%)
Missing	431 (2.7%)
Disability^d^	
Yes	1575 (9.7%)
No	14 683 (90.3%)
Number of sessions (including baseline assessment)	6.81 (2.91; 3–11)
Recovered^e^	
Yes	5903 (36.3%)
No	7730 (47.5%)
Missing	2625 (16.1%)
Reason for end of treatment	
Discharge	13 138 (80.8%)
Dropout	2328 (14.3%)
Referral to another service	792 (4.9%)
Service	
0	7027 (43.2%)
1	3402 (20.9%)
2	3244 (20.0%)
3	2585 (15.9%)

*Note*: Only the baseline values are presented for variables that were measured at multiple time points. The ‘Missing’ row was omitted if there was no missing data.

a Case thresholds were PHQ9 ⩾ 10, GAD7 ⩾ 8.

b Indicates the disorder treated: GAD = generalised anxiety disorder; PTSD = post-traumatic stress disorder; MADD = mixed anxiety and depressive disorder; panic/phobia = panic disorder, agoraphobia, social phobia, specific phobia; OCD = obsessive-compulsive disorder; ‘Other’ included somatoform disorder, severe mental illness.

c ‘Non-worker’ included homemaker, carer, retired, student.

d No negative responses were recorded; the absence of any value was counted as a negative response rather than missing.

e Only calculated for patients above case thresholds on the PHQ9 or GAD7 at baseline and with observed scores for their final session, otherwise coded as missing. Represents whether the patient reached recovery within the 11 sessions modelled; if they received more sessions and then recovered, they would appear unrecovered here.

### Growth mixture models

For both depression and anxiety symptoms, the best-fitting latent growth curve form was quadratic (online Supplementary Information S3). On average, patients' symptoms were initially moderate and improved steadily from baseline (session 0) over approximately the first five treatment sessions, showing reliable improvement by session eight. For LCGA, a four-class model was selected as the optimum solution for each outcome (online Supplementary Information S4). The trajectories were largely uninformative as they primarily differed in terms of baseline severity (intercept). We therefore estimated GMMs with free but equal variance in the intercepts; these are the focus of our results.

#### Depression symptoms growth mixture model

**Fig. 2.**
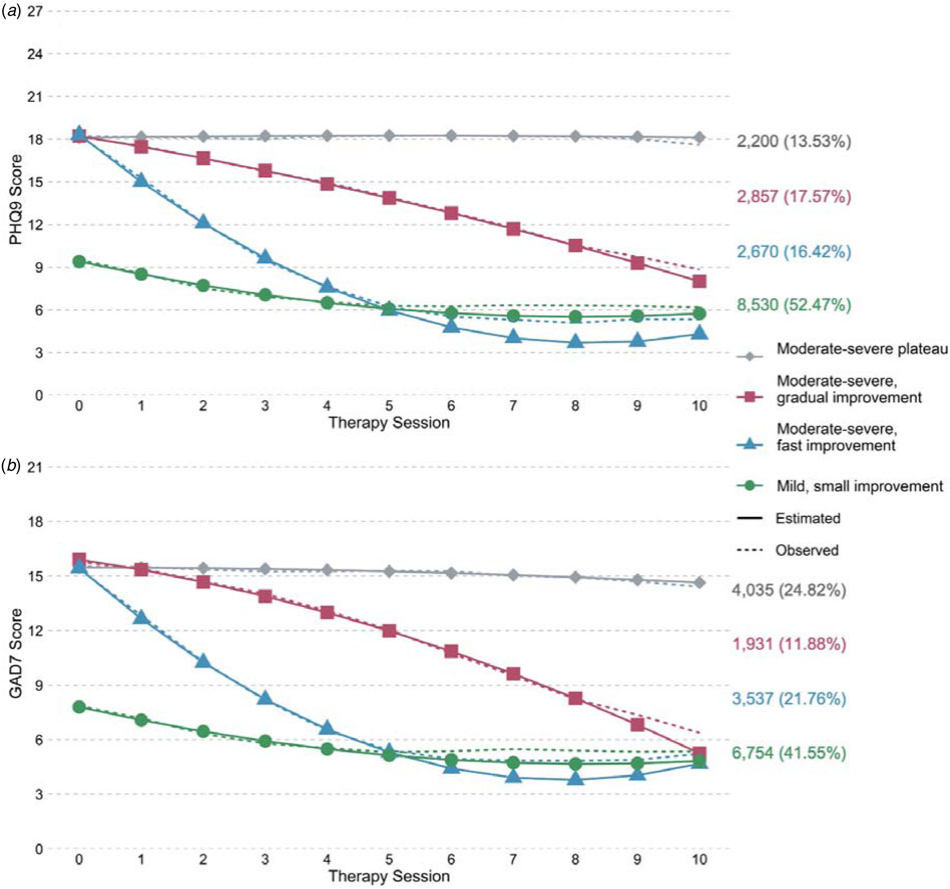
Four-class growth mixture model of (a) depression symptoms (PHQ9) and (b) anxiety symptoms (GAD7), during high-intensity psychological therapy (*N* = 16 258). *Note*: This shows the model estimated mean and observed mean trajectories of each class. Patients had a likelihood of belonging to each trajectory class; counts and proportions (%) are based on their ‘most likely’ class membership.

For depression symptoms, a four-class GMM was selected based on fit indices alongside other considerations such as previous literature (online Supplementary Information S5). Figure 2a provides the mean trajectories of the four classes. Three classes showed moderate-severe symptoms at baseline. One of these showed no change (*moderate-severe plateau*; grey diamonds), the second had steady improvement (*moderate-severe, gradual improvement*; pink squares) and the third demonstrated fast improvement initially which plateaued after the sixth treatment session (*moderate-severe, fast improvement*; blue triangles). A fourth class showed mild symptoms and minimal improvement over the first four sessions which then plateaued (*mild, small improvement*; green circles). Approximately half the sample were assigned to this *mild* class and 14–18% in each of the other classes. Reliable improvement occurred on average by the seventh treatment session in the *gradual* class, and the second in the *fast* class, but was not observed in the *plateau* or *mild* class. At the final treatment session modelled, almost no (< 1%) *moderate-severe plateau* patients had ‘recovered’ to below PHQ9 and GAD7 case thresholds used in IAPT (< 10 and < 8, respectively). Recovery was also low in the *gradual improvement* class (18.7%), but higher in the *fast improvement* and *mild* classes (67.8% and 58.8%, respectively). Dropout was higher in the *gradual improvement* and *plateau* classes (20.3% and 18.9%) than *fast improvement* and *mild* classes (14.7% and 11.0%). The mean number of sessions was, however, similar across classes. The *plateau* class had the highest proportion referred to other services (11.5%). The full descriptives from the model are in online Supplementary Information S6.

#### Anxiety symptoms growth mixture model

For anxiety symptoms, the GMM also suggested a four-class model (Fig. 2b; model selection and descriptives in online Supplementary Information S7 and S8). The trajectory classes for anxiety were very similar to that of depression and as such we used the same labels. However, more patients in the anxiety GMM were assigned to the *moderate-severe plateau* and *moderate-severe, fast improvement* classes, and fewer to the *moderate-severe, gradual improvement* and *mild, small improvement* classes. Reliable improvement occurred on average by the sixth treatment session in the *gradual* class and the second in the *fast* class but not in the *plateau* or *mild* class. Most patients who followed a *plateau* or *gradual improvement* trajectory had not recovered by the tenth treatment session (1.0% and 34.9% recovered, respectively), whilst most patients in the *fast improvement* and *mild* classes had (69.1% and 64.0%). The *plateau* class had the highest proportion of dropout (22.7%) and referral to other services (9.2%).

Online Supplementary Table S5 shows the overlap of trajectory classes between the depression and anxiety models. For example, 39.5% of patients in the depression *gradual improvement* class were in the anxiety *moderate-severe plateau* class, compared with 6.6% of the depression *fast improvement* class. In the anxiety model, 4.5% of the *gradual* and 0.8% of the *fast improvement* class were in the depression *plateau* class. Of the depression *plateau* class, 88.8% were in the anxiety *plateau* class, whilst 48.4% of the anxiety *plateau* class were in the depression *plateau* class.

### Associations of baseline variables with trajectory class

#### Conditional growth mixture model of depression symptoms

**Fig. 3.**
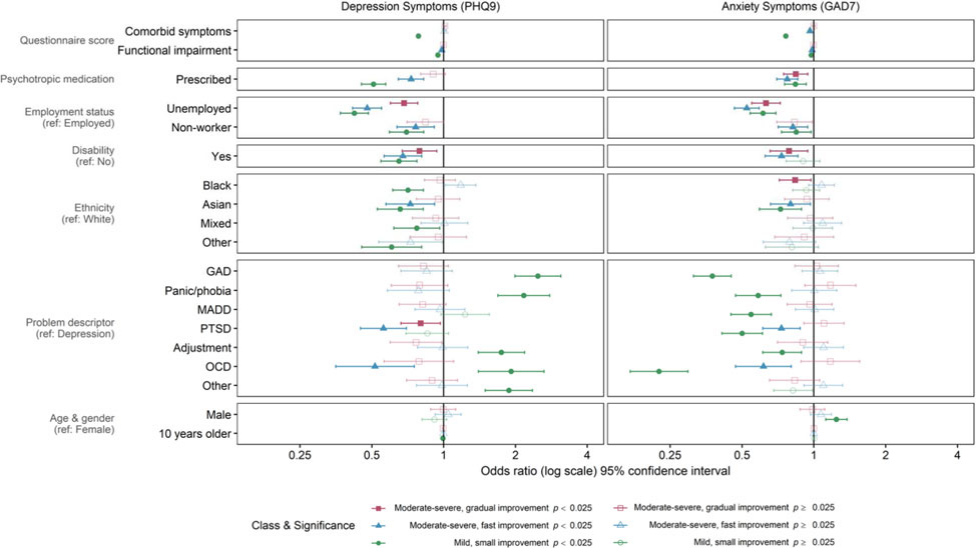
Multinomial regressions of trajectory class membership using baseline variables for a four-class growth mixture model of depression symptoms (PHQ9) and a four-class growth mixture model of anxiety symptoms (GAD7), during high-intensity psychological therapy (*N* = 16 258). *Note*: The reference class in each model was moderate-severe plateau. Service, indicating where the patient received treatment, was included as a covariate with four categories. Error bars represent 95% confidence intervals. PHQ9 = Patient Health Questionnaire 9-item version; GAD7 = Generalised Anxiety Disorder 7-item scale. Comorbid symptoms refers to baseline GAD7 score in the depression model, and baseline PHQ9 score in the anxiety model. Functional impairment was measured by the Work and Social Adjustment Scale. Employment status ‘Non-worker’ included homemaker, carer, retired and student. GAD = generalised anxiety disorder; Panic/phobia = panic disorder, agoraphobia, social phobia, specific phobia; MADD = mixed anxiety and depressive disorder; PTSD = post-traumatic stress disorder; OCD = obsessive-compulsive disorder; ‘Other’ included somatoform disorder and severe mental illness.

Higher baseline anxiety was associated with lower odds of belonging to the *mild, small improvement* class, compared with the reference class *moderate-severe plateau* (Fig. 3, online Supplementary Table S6). Patients who had higher functional impairment, were prescribed psychotropic medication, or were not working (e.g. retired) compared with employed were less likely to be in the *mild* or the *moderate-severe, fast improvement* classes. Unemployed individuals or those reporting a disability had lower odds of being in the *fast* or *gradual improvement* or *mild* classes (i.e. the more favourable classes). Compared with White ethnicity patients, patients who identified as Black, Asian, multi-ethnic (‘mixed’), or ‘other’ ethnicity were less likely to follow the *mild* trajectory compared with *moderate-severe plateau*. Patients identifying as Asian also had lower odds of belonging to the *fast improvement* class. Compared with patients with a depression problem descriptor, patients with GAD, panic/phobia, adjustment, OCD or ‘other’ disorder were more likely to be in the *mild* class. However, OCD and PTSD were associated, respectively, with decreased odds of the *fast improvement,* and *fast* or *gradual improvement* trajectories. Age and gender had no notable associations.

#### Conditional growth mixture model of anxiety symptoms

Higher baseline depression or impairment scores and non-worker status were associated with decreased odds of being in the *moderate-severe, fast improvement* or *mild, small improvement* trajectory classes than the reference, *moderate-severe plateau* (Fig. 3, online Supplementary Table S7). Patients who were unemployed or prescribed psychotropic medication were less likely to belong to any favourable trajectory class. Reporting a disability was associated with decreased odds of the *fast* or *gradual improvement* classes. Patients identifying as Black, compared with White, were less likely to be in the *gradual improvement* class. Asian ethnicity was associated with lower odds of the *fast improvement* or *mild* class. Compared with a depression problem descriptor, all descriptors besides ‘other’ were associated with lower odds of the *mild* class. Additionally, patients with PTSD or OCD were less likely to be in the *fast improvement* class. Patients reporting male gender were more likely to be in the *mild* class than females.

## Discussion

### Overview

We identified patterns of change in depression and anxiety symptoms during high-intensity psychological therapy among 16 258 patients. For both outcomes a quadratic slope was optimal and the data were best explained by four classes of trajectories whereby patients within a class exhibited similar trajectories. Baseline variables were associated with trajectory class membership.

### Growth mixture models

Both the depression and anxiety models had three classes with moderate-severe symptom scores at baseline. Using only intake symptom severity would therefore not allow a clinician to distinguish between three very different trajectories. One of these trajectories showed no change, labelled *moderate-severe plateau*. The other two classes reliably improved: *moderate-severe, gradual improvement* changing more steadily than *moderate-severe, fast improvement*. The fourth class, *mild, small improvement*, had average baseline symptoms below clinical thresholds, and thus less capacity for large improvements. These trajectory classes were broadly consistent in number and shape with a previous IAPT study (Saunders et al., 2019), despite several sample and methodological differences in our study, including a greater proportion of patients receiving counselling and fewer receiving CBT.

The *gradual improvement* class may be particularly clinically relevant as the apparent lack of early response could lead to premature alteration or termination of treatment. Symptom change was initially indistinguishable from the *moderate-severe plateau* trajectory and only showed reliable improvement after six or seven sessions. ‘Recovery’ represents both depression and anxiety symptom scores falling below clinical thresholds by the final session. Recovery was lower in the *gradual* than the *fast improvement* class, especially for the depression model. Reflecting this, patients in the *gradual* class were less likely to experience improvement in the *other* outcome. This can be seen in the higher proportion of the depression *gradual* class than *fast improvement* class in the anxiety *plateau* class. The *gradual improvement* class might represent patients who require a high number of treatment sessions to show recovery. Supporting this, a study of high-intensity CBT found that most patients who significantly improved did so within 14 sessions (Robinson et al., 2020b) and a systematic review of therapy dose recommended up to 26 sessions for patients who have improved but not recovered before this point (Robinson, Delgadillo, & Kellett, 2020a).

The *moderate-severe plateau* class may also represent patients who would benefit from more treatment, although some ‘non-responders’ would probably not recover even after a very high number of sessions (Howard, Kopta, Krause, & Orlinsky, 1986). Notably, the frequency of onward referrals in this class suggested the presence of co-occurring problems that required specialist services. That said, despite limited symptom change, some patients may have experienced other, unmeasured, benefits such as prevention of deterioration or hospitalisation. More patients belonged to the *plateau* class for the anxiety than the depression model. This may be driven by disorders such as OCD and PTSD, which on average require a greater dose of therapy to observe improvement than most depression and anxiety disorders (Robinson et al., 2020b), and are better detected by the GAD7 than the PHQ9 (Kroenke, Spitzer, Williams, Monahan, & Löwe, 2007). However, slightly over half of the anxiety *plateau* class showed mild or improving depression symptoms, whilst most patients in the depression *plateau* class showed no improvement on either measure as were also in the anxiety *plateau* class.

Patients in the *fast improvement* class generally appeared to require fewer than ten treatment sessions. They were below case thresholds after an average of four treatment sessions and had the highest recovery rate, in-line with meta-analytic evidence that reliable symptom improvement by the fourth session predicts recovery (Beard & Delgadillo, 2019). It is important to continue treatment for several sessions after remission to help prevent false positives or relapse (Robinson et al., 2020b), however, this finding warrants further investigation as it could have implications for service efficiency.

The mean initial symptom score estimated by the model for the *mild, small improvement* trajectory class, for both depression and anxiety models, was below case threshold, yet all patients had received high-intensity therapy. This was consistent with the observed baseline means. However, it would be inappropriate to conclude that high-intensity treatment was unwarranted for these patients. Scores varied around these means and many patients met the case threshold on the other symptom measure; the majority of *mild* class patients were an observed case on one or both measures. These patients may also have had additional symptoms not assessed by the PHQ9 or GAD7.

### Associations of baseline variables with trajectory class

The most consistent associations with trajectory class membership were prescribed medication, reporting a disability, and not being in employment, which were generally associated with lower odds of being in any of the more favourable classes compared with *moderate-severe plateau*. These associations are especially informative as they discriminated between individuals with similarly high baseline scores who improved or not. The association with employment status was consistent with findings from endpoint treatment outcome studies (Buckman et al., 2022; Delgadillo et al., 2016; Delgadillo, Huey, Bennett, & McMillan, 2017b). The prior IAPT trajectory study found no association with medication (Saunders et al., 2019) despite associations with treatment outcomes in endpoint studies (Robinson et al., 2020b). Neither employment or disability were included in the prior trajectory study, and the present findings highlight the importance of routinely recording them. These factors may negatively impact therapy response and acknowledging and supporting patients to navigate them might reduce the likelihood of a *moderate-severe plateau* trajectory. This supports the argument for psychological services working closely with employment advisors, as per the IAPT manual (NCCMH, 2021).

Higher functional impairment was associated with lower odds of the *fast improvement* or *mild* class than the *plateau* class, but not the *gradual improvement* class. This variable may therefore be useful for discriminating between *fast* and *gradual improvement* for patients with similar baseline symptoms. The effect sizes for functional impairment were smaller than those for symptom scores, but still notable given they reflected the association for a one-unit difference on the WSAS, which has a wide scoring range. In the previous IAPT trajectory study, higher functional impairment and symptom scores similarly showed greater likelihood of the corresponding *moderate-severe plateau* class (Saunders et al., 2019). Our results were also consistent with a study reporting that patients with higher baseline scores on the PHQ9, GAD7, WSAS, prescribed antidepressants, a disability or unemployment were less likely to improve with an increasing number of sessions, similar to our *plateau* class (Robinson et al., 2020b).

The associations between *mild* trajectory class and problem descriptor in each model were ecologically valid. For example, patients were more likely to be in the *mild* trajectory class of the depression model if they had an anxiety descriptor compared with depression. Additionally, PTSD and OCD were associated with lower odds of a more favourable trajectory class. Improvement may have been observed after a greater number of sessions than modelled here, consistent with existing literature (Robinson et al., 2020b), or using measures more sensitive to symptoms of PTSD and OCD.

Patients who identified as one of the minoritised ethnic groups rather than White ethnicity were less likely to follow a favourable trajectory, especially the *mild* depression class, indicating that they had more severe symptoms at intake. These findings are consistent with lower recovery rates in national IAPT data and indicate that some patients from minoritised ethnic groups may benefit from culturally-adapted treatment (Arundell, Barnett, Buckman, Saunders, & Pilling, 2021; Beck, Naz, Brooks, & Jankowska, 2019). The ethnic diversity of the sample allowed us to study ethnicity in more detail than the binary variable used in similar studies and therefore identify that this pattern was most pronounced for Asian patients. That notwithstanding, there was insufficient power to analyse associations with ethnicity at an even greater level of granularity and this will require further research.

Overall, some variables were generally associated with greater likelihood of the *plateau* trajectory class than any more favourable class. These included reporting a disability, unemployment or prescribed medication. Other baseline characteristics differentiated between the more favourable classes, including non-worker status, functional impairment, specific problem descriptors and ethnicity.

### Strengths and limitations

A large sample size enabled us to produce more accurate estimates and include a broader range of ethnicity categories and baseline variables, such as employment and disability, compared with previous studies. These important and easily recorded factors could help identify likely symptom trajectories at the start of treatment. However, the results may be confounded by the presence of additional variables indicative of treatment prognosis, such as chronicity (Kessler et al., 2017; Lorenzo-Luaces, Rodriguez-Quintana, & Bailey, 2020). Whilst routinely collected data may generalise to clinical settings more readily than clinical trial data, the provision of different interventions, dependent on problem descriptor, makes IAPT data more complex to analyse. Investigations of treatment and descriptor-specific trajectories were beyond the scope of this study. The inclusion of patients with a depression descriptor in the anxiety symptoms model, and vice versa, may therefore have contributed to the high proportion in the *mild* trajectory classes. Although comorbidity is common between depression and anxiety, the extent of comorbidity was unknown in the current sample as the IAPT problem descriptor only represents the condition being treated. That notwithstanding, the assessment of transdiagnostic symptoms appears particularly important for treatment outcomes (O'Driscoll et al., 2021) so we chose to include all patients in both models. Our findings therefore offer a broad representation of what we expect to see in routine clinical settings. The reliability and validity of IAPT problem descriptors has not been established. However, clinicians are trained to use ICD-10 to determine them, and in services with a higher frequency and accuracy of providing problem descriptors, patients are more likely to recover or reliably improve (Clark et al., 2018; Saunders et al., 2020b).

In terms of ethnicity, although generally representative of the local population that SLaM IAPT serves (Office for National Statistics, 2012), the patient sample was more ethnically diverse than IAPT nationally. The two most frequently reported ethnicities in the sample were White (60%) and Black (18%), and in IAPT national data are White (80%) and Asian (6%) (NHS Digital, 2021). It is critical that mental health and treatment outcome research is applicable for all patients, and oversampling from minoritised groups is one way of achieving this. In terms of ethnicity, this overrepresentation occurred naturally within our study, and we view the ethnic diversity of this sample as a strength. Additionally, although caution is recommended when using the IAPT measures across cultures (Beck et al., 2019), the PHQ9 and GAD7 have demonstrated good reliability and validity across a range of communities (see Beck et al., 2019 for a summary).

Another issue was dropout. Dropout is also observed in clinical trials and can occur due to different reasons, including dissatisfaction with treatment or recovery before the clinically recommended number of sessions. Regardless, and despite the use of missing data methods, scores at later sessions were based on remaining individuals. Replication in a sample receiving a fixed treatment length is therefore required.

Finally, the GAD7 does not cover core symptoms for disorders such as OCD and PTSD. However, it does show reasonably high specificity and sensitivity for several disorders, including these (Kroenke et al., 2007), and therefore provides some indication of likely trajectories for them. Future studies should aim to model trajectories of disorder specific symptoms, and also functional impairment, which is particularly important to patients and potentially improves later in treatment than symptoms (Howard, Lueger, Maling, & Martinovich, 1993). This was not possible in the present sample due to the extent of missingness on these variables. Modelling a higher number of sessions would also be beneficial, to reveal more about patients who require a greater dose of therapy.

## Implications and conclusions

Using a person-centered analysis allowed us to identify substantial heterogeneity in depression and anxiety symptom change during psychological therapy which could be explained by four classes of trajectories. The identified trajectory classes were differentially associated with baseline variables such as employment and ethnic group. To be implemented clinically, these findings need to be extended into a predictive tool that combines baseline variables and outputs a patient's most likely symptom trajectory. Once validated in an independent dataset, this could be used to inform treatment plans at the start of therapy and also monitor whether patients are ‘on-track’ according to their predicted trajectory, which has been shown to improve patient outcomes (Delgadillo et al., 2018). This would be especially relevant for patients in the gradual improvement class who on average did not show reliable improvement until session six or seven. This study is therefore a crucial first step towards clinical use of trajectory classes to ultimately improve patient outcomes and service efficiency.

## Data Availability

This study used anonymised patient data from the SLaM NHS Foundation Trust that is not publicly available. The data can be accessed within a secure firewall via the Clinical Record Interactive Search (CRIS) tool by persons with the appropriate permissions. For more information please contact: cris.administrator@slam.nhs.uk.

## Supplementary material

For supplementary material accompanying this paper visit https://doi.org/10.1017/S0033291722003403. click here to view supplementary material
