# Supplementary material for: Trajectories of depression and anxiety symptom severity during psychological therapy for common mental health problems
Source: Psychol Med. 2022 Dec 13;53(13):6183–93. doi: 10.1017/S0033291722003403 (PMC10520600; doi:10.1017/S0033291722003403)
Supplement: Supplementary file 1 [file S0033291722003403sup001.docx]

**Supplementary materials for “Trajectories of depression and anxiety symptom severity during psychological therapy for common mental health problems”**

# [Supplementary Information 1. Data pre-processing and additional exclusion criteria](#SI1)

# [Supplementary Table 1. Number of time points (sessions) for patients in the analysis](#SI4)

# [Supplementary Table 2. Descriptives of time intervals between time points (sessions) for patients in the analysis](#_Supplementary_Table_2._1)

# [Supplementary Information 2: Growth mixture model method](#SI5)

# [Supplementary Figure 1 (a - b). Latent growth curves of depression and anxiety symptoms by the four treatment sites, and latent class growth analysis of depression symptoms for each site](#SI6)

# [Supplementary Figure 2. Histograms of observed depression and anxiety symptoms per time point (session)](#SI7)

# [Supplementary Table 3. Proportion of patients in treatment at each time point (session) with observed depression and anxiety symptoms](#SI9)

# [Supplementary Table 4. Comparison of baseline characteristics of patients from the original data extraction who were excluded from analysis to patients included in the analysis](#SI10)

# [Supplementary Information 3. Latent growth curves of depression and anxiety symptoms](#SI11)

# [Supplementary Information 4. Latent class growth analysis of depression and anxiety symptoms](#SI12)

# [Supplementary Information 5. Selection of depression symptoms growth mixture model](#SI13)

# [Supplementary Information 6. Descriptives of four-class growth mixture model of depression symptoms](#SI14)

# [Supplementary Information 7. Selection of anxiety symptoms growth mixture model](#SI15)

# [Supplementary Information 8. Descriptives of four-class growth mixture model of anxiety symptoms](#SI16)

# [Supplementary Table 5. Overlap of patients' most likely class for four-class growth mixture models of depression and anxiety symptoms](#SI17)

# [Supplementary Table 6. Multinomial regression output of four-class growth mixture model of depression symptoms](#SI18)

# [Supplementary Table 7. Multinomial regression output of four-class growth mixture model of anxiety symptoms](#SI19)

# Supplementary Information 1. Data pre-processing and additional exclusion criteria

We initially extracted treatment records for 115,304 patients covering 587,120 sessions. We performed extensive data pre-processing, as is often required for electronic health records, and imposed additional exclusion criteria, under guidance from experienced IAPT clinicians. A core component was classification of free-text responses. For example, the intervention variable exceeded 100 distinct values, which required mapping to categories of high-intensity, low-intensity, and non-intervention (e.g., triage). We were then able to restrict analysis to individuals who had received high-intensity therapy, which was required due to the difference in the average number of sessions and level of structure between intensities. We imposed additional exclusion criteria to patients and sessions to obtain valid outcome data suitable for growth mixture modelling. We did not limit analyses to individuals who scored above clinical thresholds on the PHQ9 or GAD7 at baseline, as this would not accurately reflect the IAPT population. The resulting sample for analysis consisted of 16,258 patients covering 110,773 sessions. The largest exclusion was patients who did not receive at least two sessions of high-intensity therapy (N = 94,979). We ideally would have limited our original extraction to such individuals but were unable to due to the lack of a reliable variable indicating treatment intensity. Furthermore, we could not perform this exclusion earlier in the pre-processing as valid treatment sessions had to first be identified to count the number of each intensity. Several variables of interest were not included in analyses due to high, potentially non-random missingness e.g., at baseline the social phobia, agoraphobia and specific phobia items were each 49% missing. Data pre-processing and descriptives were performed in R version 3.6.3.

# Supplementary Table 1. Number of time points (sessions) for patients in the analysis (N = 16,258)

| **Total sessions received** | **Frequency** | **Proportion (%)** | **Cumulative Proportion (%)** |
| --- | --- | --- | --- |
| 3 | 1885 | 11.59 | 11.59 |
| 4 | 1804 | 11.10 | 22.69 |
| 5 | 2153 | 13.24 | 35.93 |
| 6 | 2706 | 16.64 | 52.58 |
| 7 | 2046 | 12.58 | 65.16 |
| 8 | 1080 | 6.64 | 71.80 |
| 9 | 884 | 5.44 | 77.24 |
| 10 | 717 | 4.41 | 81.65 |
| 11 | 2983 | 18.35 | 100.00 |

*Note:* To identify a sample who received high-intensity therapy, patients were included in the analysis if they had attended at least three sessions, with at least two being high-intensity treatment (and permitting one low-intensity and/or one assessment and triage session). The **mean number of sessions** was 7.32 (SD = 3.79) and ranged 3-55. The number of sessions was then limited to maximum 11, including the baseline assessment, to be reasonably representative of the number received in the sample (within 1 SD of mean; < 15% received more than 11), and to provide sufficient complete data for analysis using maximum likelihood estimation for missing data (covariance coverage above Mplus default of 0.10). Following this, the mean was 6.81 sessions (SD = 2.67).

# Supplementary Table 2. Descriptives of time intervals (days) between time points (sessions) for patients in the analysis (N = 16,258)

|  | **Time point (Session)** | | | | | | | | | | |
| --- | --- | --- | --- | --- | --- | --- | --- | --- | --- | --- | --- |
| **Dataset** | 0 | 1 | 2 | 3 | 4 | 5 | 6 | 7 | 8 | 9 | 10 |
| Unfiltered | 0.00 (0.00) | 31.21 (50.82) | 19.47 (33.80) | 13.28 (17.03) | 12.72 (15.39) | 12.93 (17.90) | 13.13 (15.86) | 13.44 (15.27) | 13.33 (14.07) | 13.89 (16.54) | 13.87 (17.24) |
| Filtered | 0.00 (0.00) | 12.53 (7.17) | 10.41 (5.68) | 10.27 (5.51) | 10.16 (5.40) | 10.13 (5.48) | 10.23 (5.46) | 10.59 (5.78) | 10.46 (5.63) | 10.46 (5.72) | 10.46 (5.64) |

*Note:* Mean (standard deviation) days between column-specified session and previous session. Descriptives are provided for patients *prior* to filtering out sessions occurring after intervals exceeding 30 days (unfiltered) and *following* this (filtered). The filtered dataset was used for analysis. In both datasets we had previously removed the baseline session if there was an interval exceeding 30 days to session 1 as this had the separate purpose of identifying a baseline session (the unfiltered mean interval here exceeds 30 as the baseline to session 1 filter was performed only once therefore the ‘new’ session 1 could have occurred after a long interval from the previous session 1).

# Supplementary Information 2: Growth mixture model method

We estimated separate growth mixture models (GMMs) for anxiety symptoms and depression symptoms. The first step of GMM is latent growth curve analysis, which is used to identify the best-fitting single, average, latent growth curve (trajectory). The latent growth curve consists of an estimated mean intercept and estimated mean slope. This trajectory describes the pattern of symptom change observed across all patients as though they are one homogeneous group. Latent growth curves of different forms (linear, quadratic, and negative log-linear (base 10)) were estimated and compared to determine which most closely represented the observed data. Each form was run with and without correlations between the residuals of adjacent time points. Model fit was assessed using Akaike’s Information Criterion (AIC), Bayesian Information Criterion (BIC), Standardised Root Mean Square Residual (SRMR) and Root Mean Square Error of Approximation (RMSEA). Lower values indicate superior fit, with recommendations of SRMR ≤ 0.08 and RMSEA ≤ 0.06 (Hu & Bentler, 1999; Nylund, Asparouhov & Muthén, 2007; Schwarz, 1978). We also used the Comparative Fit Index (CFI) and Tucker-Lewis Index (TLI), where values closer to 1 indicate better fit, ideally ≥ 0.95 (Hu & Bentler, 1999).

The next step of GMM is to determine whether patients’ observed data is better explained by multiple latent growth curves than a single average one. This is done by introducing a factor of ‘class’ into the model. In our models, each class had a trajectory of the best-fitting form identified in the first step, but with its own specific mean intercept and slope. In GMM, the variance within the intercept and/or slope of each trajectory class is free, allowing varying expressions of estimated individual trajectories around the mean within a class. A trajectory class therefore represents multiple similar trajectories. In a restricted version of GMM called latent class growth analysis (LCGA), the within-class variance is fixed to zero and thus all individuals in a class follow exactly the same trajectory. LCGA is less computationally demanding and can provide a useful representation of the true trajectories. However, it is unlikely that individuals within a class follow exactly the same trajectory (Muthén, 2002; Nagin & Odgers, 2010) and LCGA can result in spurious classes, and classes that differ only by intercept, as only classes can explain variance in the data (Muthén, 2002; Bauer & Curran, 2004). We therefore followed recommendations to first perform LCGA and inspect the results to determine whether to run GMM.

We modelled up to six trajectory classes for LCGA and GMM. Each class model was estimated using a series of steps to help ensure global as opposed to local solutions (see below) and then was compared to the model with one fewer class. The model fit criteria were AIC, BIC, and Vuong-Lo-Mendell-Rubin Likelihood Ratio Test (VLMR-LRT; Mplus ‘TECH11’). A significant *p*-value (< 0.05) for the VLMR-LRT indicates that the current estimated model is a significantly better fit of the observed data than a model with one fewer class. Where there is disagreement between indices, there is a preference in the literature for BIC (van de Schoot et al., 2017). However, it is possible with large sample sizes that as the number of classes increases, information criteria values fail to reach a minimum, in which case it can be useful to identify the point of diminishing decreases in an elbow plot (Meyer & Morin, 2016; Petras & Masyn, 2010).

Entropy was inspected to describe the distinction between classes but was only consulted for model selection when other indices were similar between models (Petras & Masyn, 2010; van de Schoot et al., 2017). In GMM, each individual has a likelihood, ‘posterior probability’, of belonging to each class i.e., of following an estimated trajectory from that class. Entropy is based on individual posterior probabilities and ranges between 0 (equal probability of belonging to each class) and 1 (distinct classifications). A common rule of thumb is that > 0.8 indicates high class separation, 0.6 medium, and 0.4 low (Clark & Muthén, 2009). We favoured models that were clinically interpretable and reasonable in terms of theory and previous literature. More parsimonious models were preferred, especially if a model had an additional class with a similar slope to one in the previous model and only the baseline score (intercept) differed. To aid this process, we plotted the mean estimated trajectory of each class per model.

## *Procedure for conducting growth mixture modelling in Mplus*

To help ensure global as opposed to local solutions, the following procedure was used in Mplus (Version 8.3) for one- to six-class GMMs (based on Asparouhov & Muthén, 2012; Jung & Wickrama, 2008; Wickrama et al., 2016):

1. Run model with 400 initial stage random starting values and 100 top log-likelihood values brought to the final stage, as recommended, with maximum 10 iterations.
2. Check whether the best log-likelihood value replicates. Check for normal termination of model estimation, negative residual variances and other warnings or errors in the output. If the log-likelihood does not replicate, increase the number of random starts in both stages until it does.
3. Once the log-likelihood replicates, check that it is not a local solution by taking the seed values from the top two log-likelihood values and running the model again, using the optseed argument in Mplus and the starts argument set to zero. At this stage, include TECH11 (the VLMR test).
4. Ensure that the log-likelihood value for the K-1 class in the TECH11 output matches that of the actual K-1 class model. If not, increase the starting values in the K-1 starts argument of the input.

# Supplementary Figure 1 (a - b). Latent growth curves of depression (PHQ9) and anxiety (GAD7) symptoms per treatment site, and latent class growth analysis of depression symptoms for each site

Latent growth curves for symptoms of patients from each of the four IAPT treatment sites were highly similar (Figure a). Latent-class growth analysis was run for one outcome (PHQ9) and showed similar trajectories (see four-class models in Figure b).


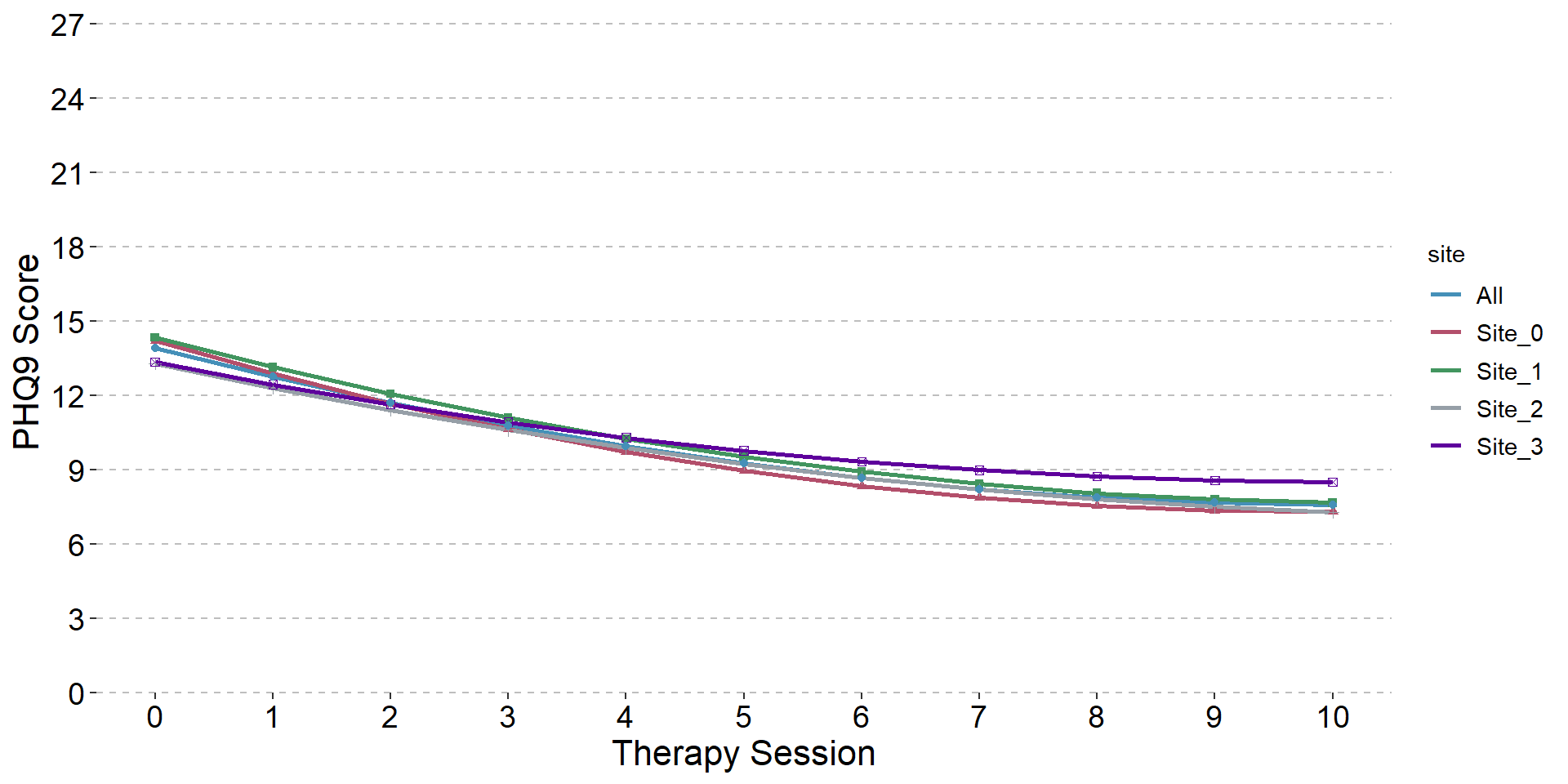

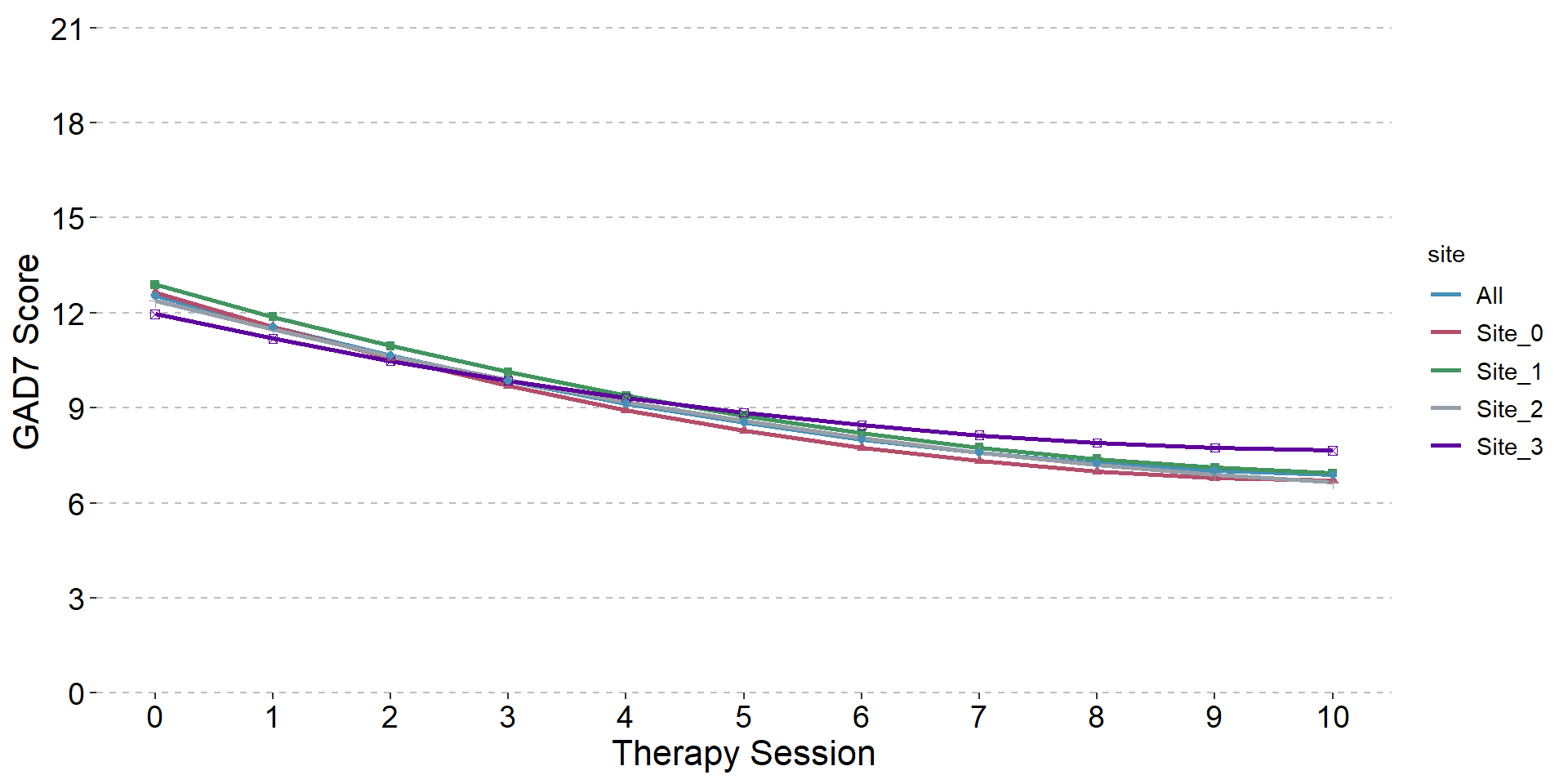


1.
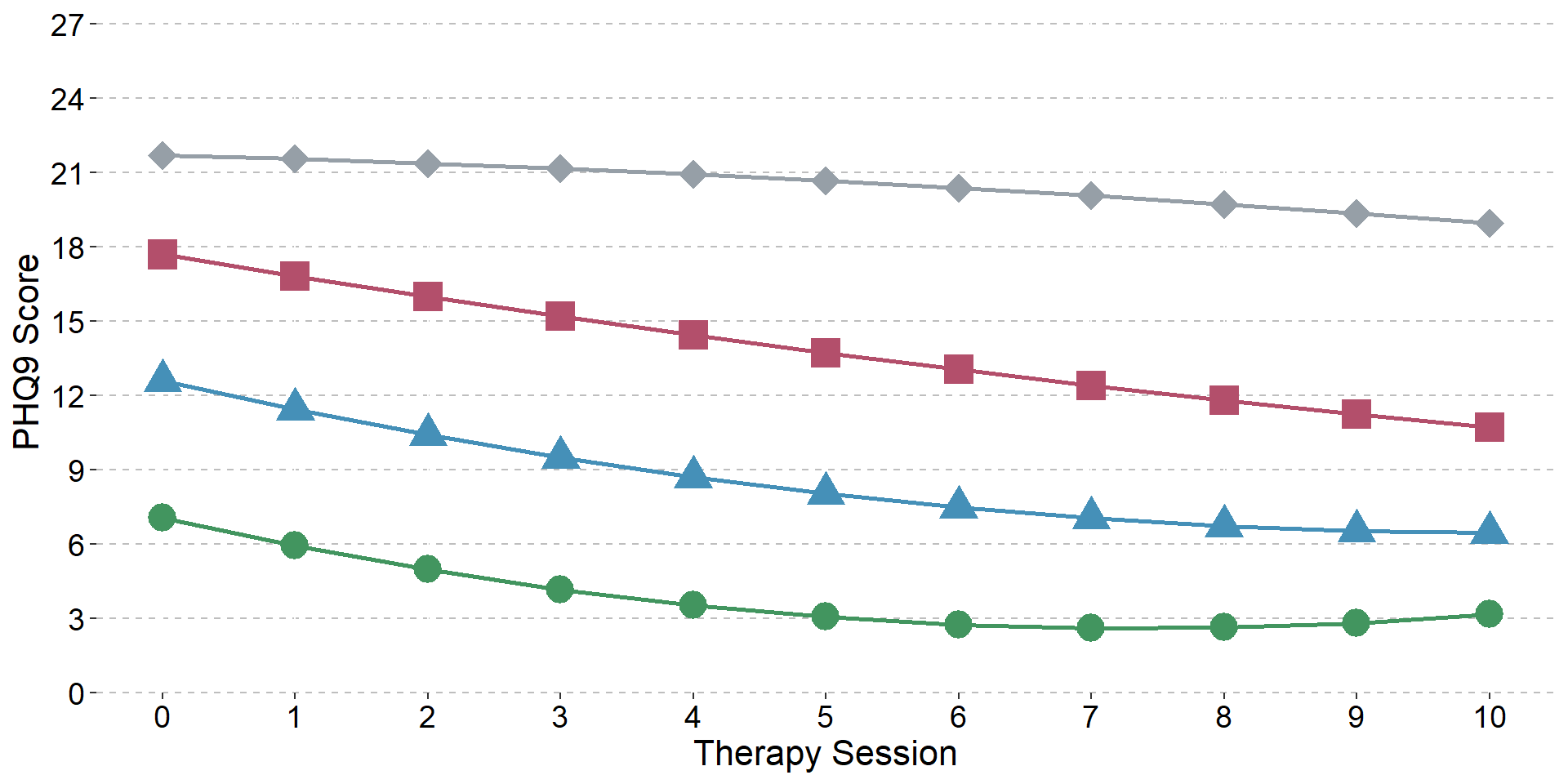

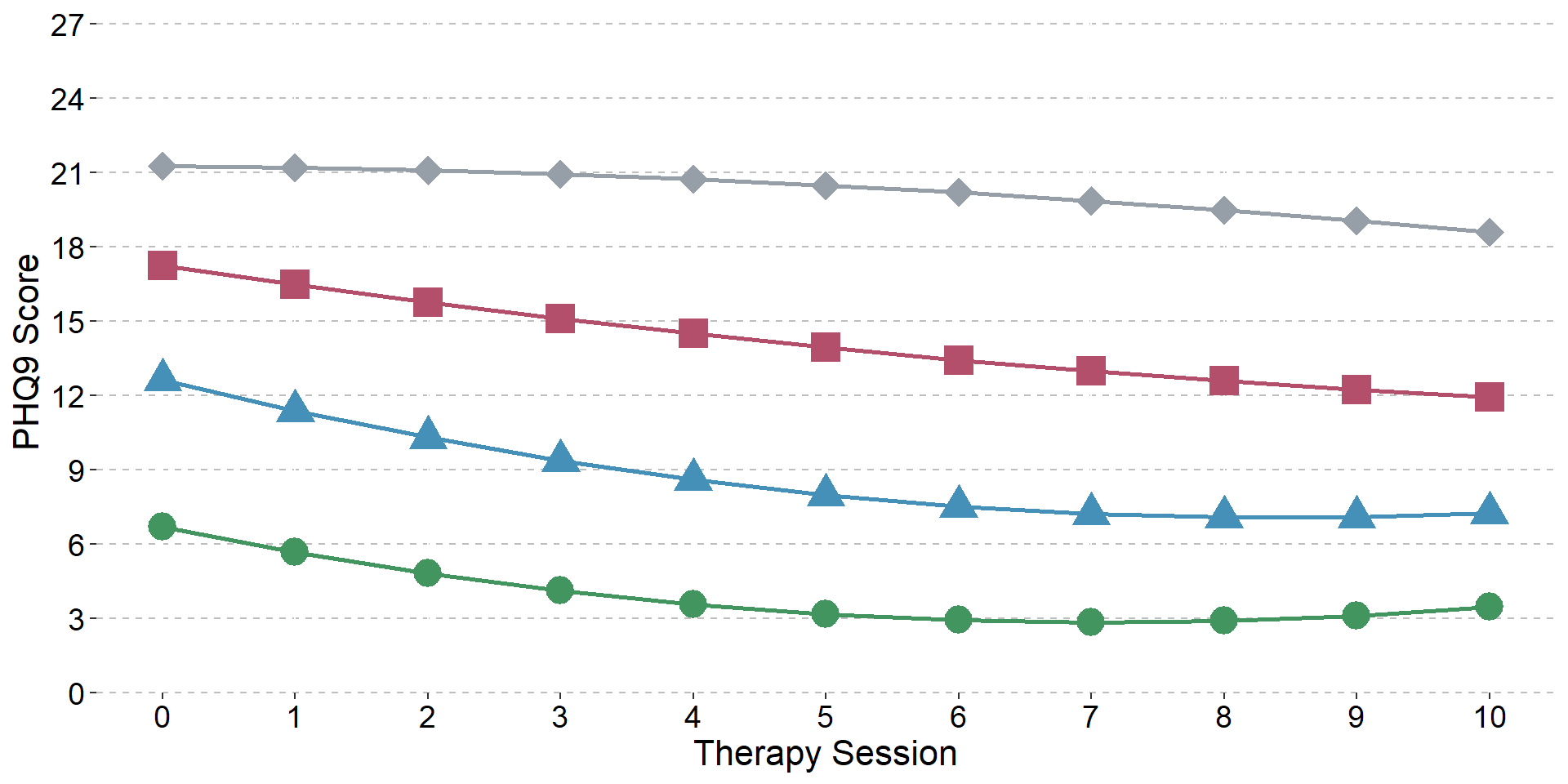

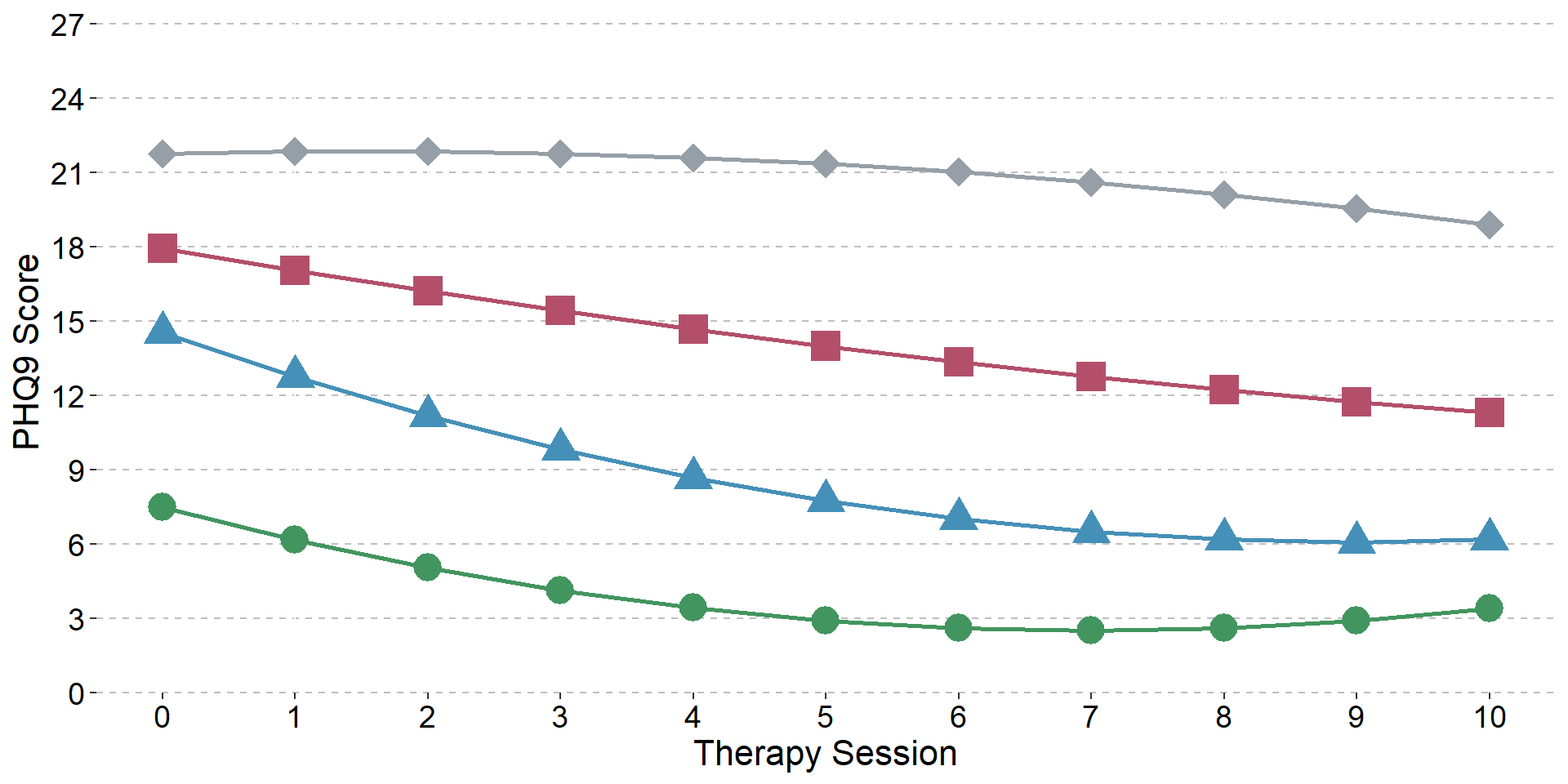

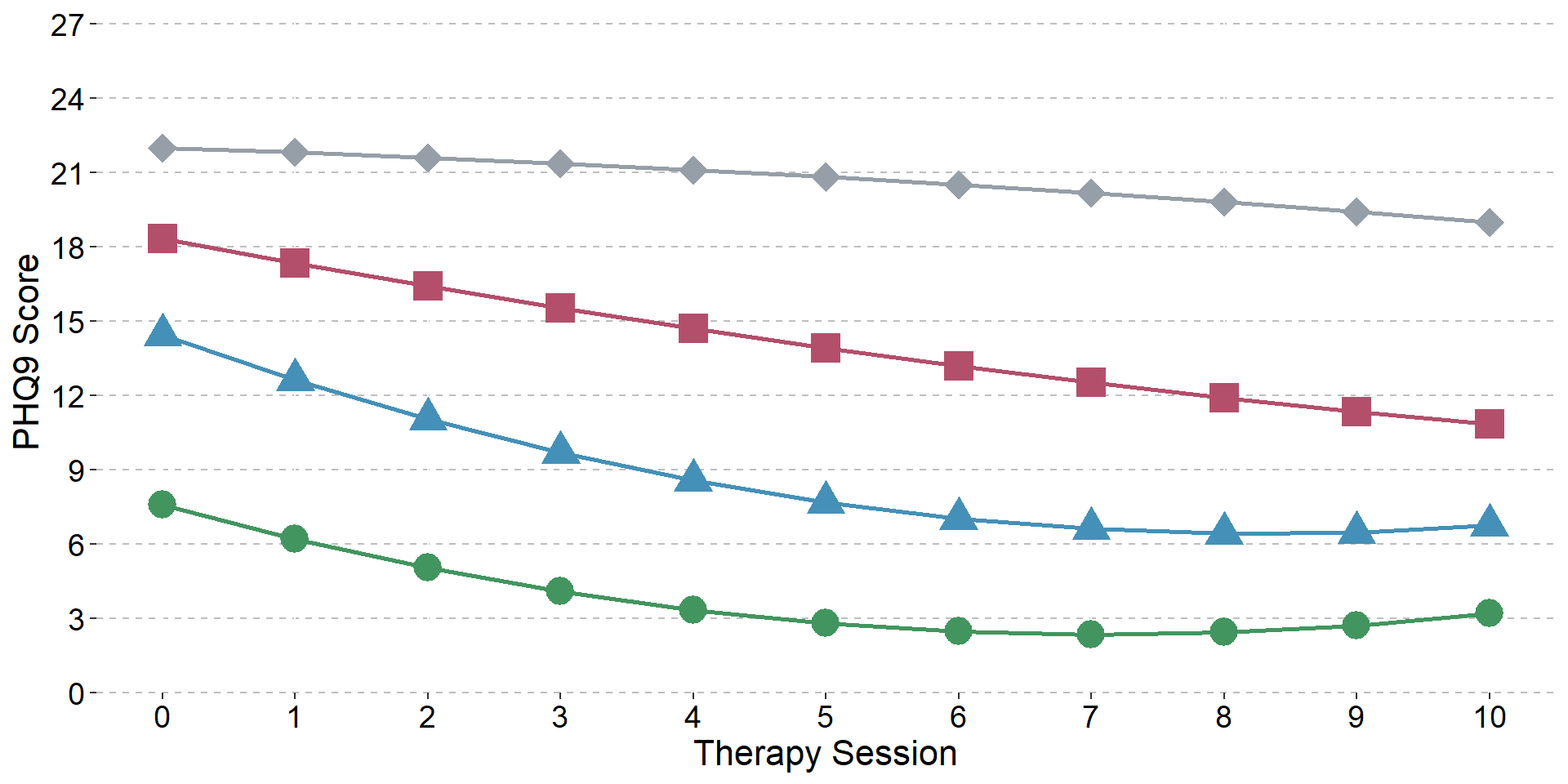
Latent growth curves of depression symptoms (PHQ9, left) and anxiety symptoms (GAD7; right), per treatment site and across all sites (‘All’)
2. Four-class latent class growth analysis of depression symptoms (PHQ9), for each IAPT treatment site

# Supplementary Figure 2. Histograms of observed depression (PHQ9; left) and anxiety symptoms (GAD7; right) per time point (session)


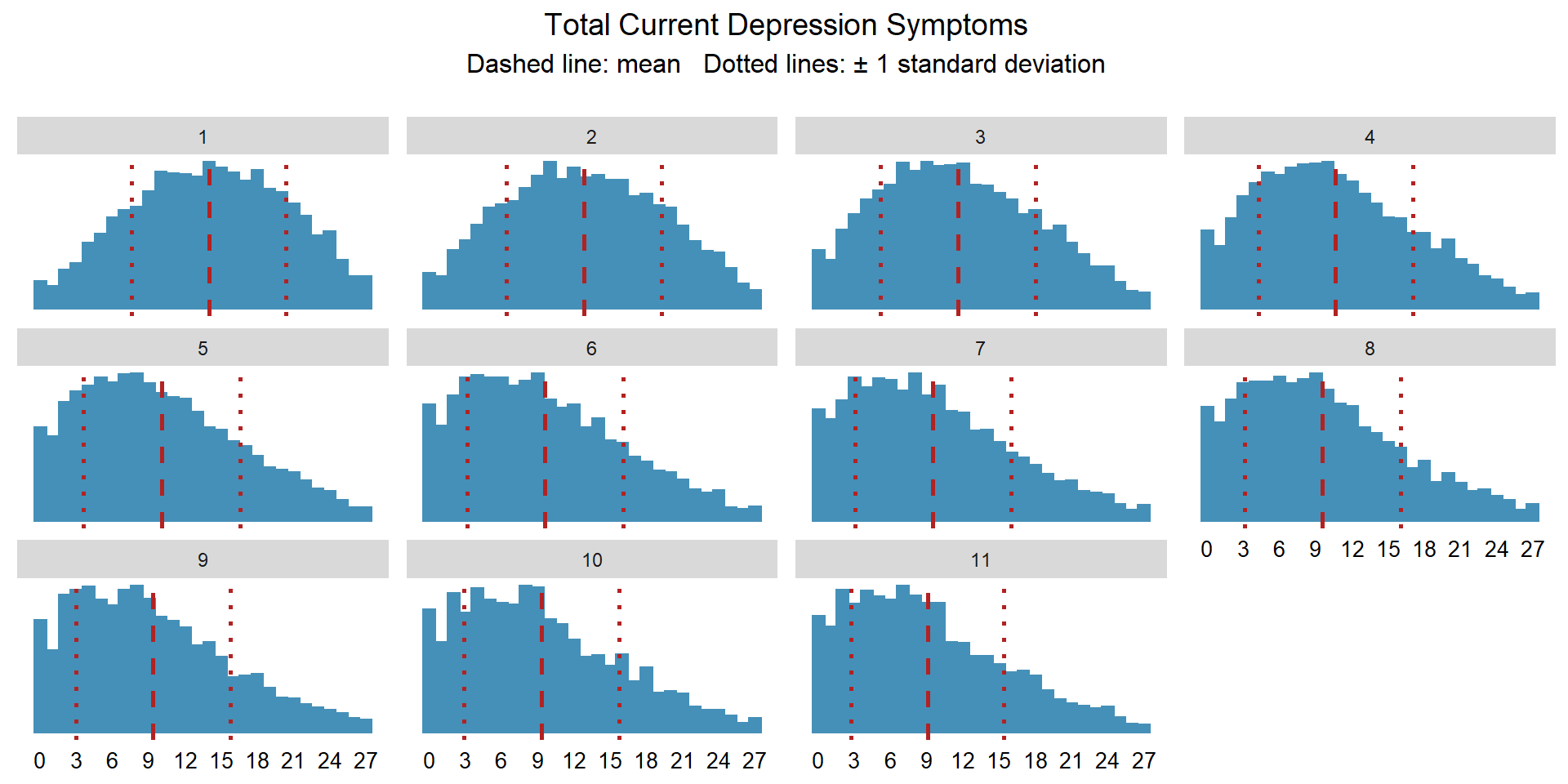

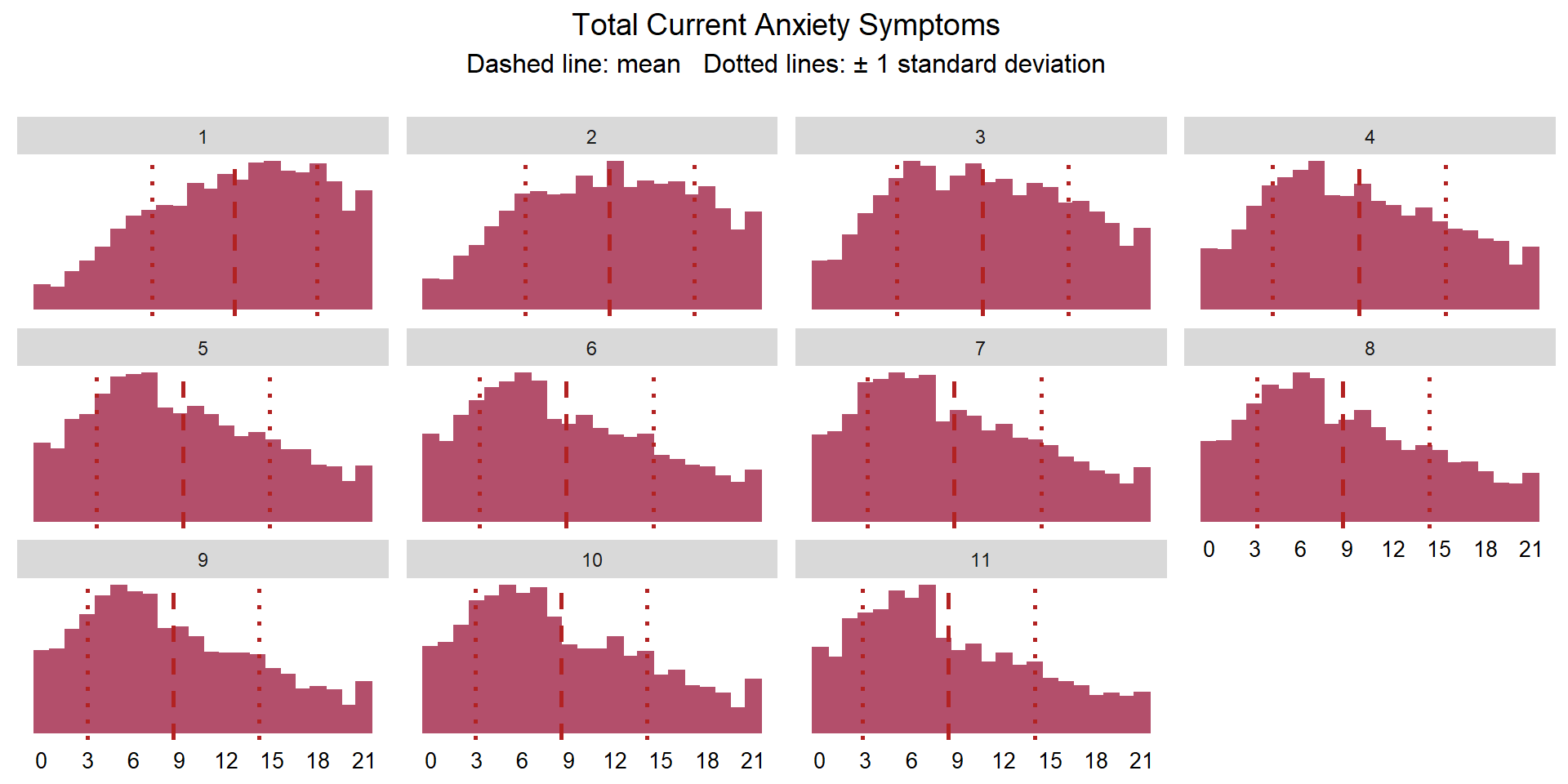


*Note*: Histograms for total symptom scores measured across 11 time points (baseline and 10 treatment sessions). Only observed scores for patients in treatment at each time point are shown; see Supplementary Table 3 for Ns.

# Supplementary Table 3. Proportion of patients in treatment at each time point (session) with observed depression (PHQ9) and anxiety (GAD7) symptoms

| **Time point (session)** | **Number of patients in treatment** | **Proportion of patients  with PHQ9 score** | **Proportion of patients  with GAD7 score** |
| --- | --- | --- | --- |
| 0 | 16258 | 99.06% | 99.05% |
| 1 | 16258 | 99.48% | 99.48% |
| 2 | 16258 | 99.38% | 99.37% |
| 3 | 14373 | 99.52% | 99.49% |
| 4 | 12569 | 99.53% | 99.51% |
| 5 | 10416 | 99.65% | 99.64% |
| 6 | 7710 | 99.52% | 99.49% |
| 7 | 5664 | 99.35% | 99.35% |
| 8 | 4584 | 99.50% | 99.48% |
| 9 | 3700 | 99.70% | 99.70% |
| 10 | 2983 | 99.63% | 99.63% |

# Supplementary Table 4. Comparison of baseline characteristics of patients from the original data extraction who were excluded from analysis to patients included in the analysis

| **Variable** | **Excluded from analysis**  **(n = 94,979)** | **Included in analysis**  **(n = 16,258)** | ***p*-value** | **Effect size** | |
| --- | --- | --- | --- | --- | --- |
| **Age (years)** |  |  | < 0.001 | 0.12 | |
| Mean (SD; range) | 35.96 (13.47; 16.00-100.00) | 37.55 (13.36; 16.00-94.00) |  |  | |
| **Gender** |  |  | < 0.001 | 0.02 | |
| Female | 61801 (65.18%) | 10979 (67.60%) |  |  | |
| Male | 33019 (34.82%) | 5262 (32.40%) |  |  | |
| Missing | 159 | 17 |  |  | |
| **Depression symptoms (PHQ9)** | |  | 0.239 | 0.01 | |
| Mean (SD; range) | 13.91 (6.48; 0.00-27.00) | 13.98 (6.39; 0.00-27.00) |  |  | |
| Missing | 2829 | 153 |  |  | |
| **Anxiety symptoms (GAD7)** |  |  | 0.771 | < 0.01 | |
| Mean (SD) | 12.52 (5.46; 0.00 - 21.00) | 12.53 (5.39; 0.00 - 21.00) |  |  | |
| Missing | 2885 | 154 |  |  | |
| **Case on PHQ9 and/or GAD7** ^1^ |  |  | 0.679 | < 0.01 | |
| Yes | 78434 (85.17%) | 13735 (85.29%) |  |  | |
| No | 13658 (14.83%) | 2368 (14.71%) |  |  | |
| Missing | 2887 | 155 |  |  | |
| **Functional impairment score (WSAS)** | |  | < 0.001 | 0.06 | |
| Mean (SD; range) | 18.15 (9.56; 0.00 - 40.00) | 17.58 (9.31; 0.00 - 40.00) |  |  | |
| Missing | 25282 | 5447 |  |  | |
| **Problem descriptor** ^2^ | |  | < 0.001 | 0.14 | |
| Depression | 35509 (43.62%) | 6703 (45.74%) |  |  | |
| GAD | 17181 (21.11%) | 1393 (9.51%) |  |  | |
| Other | 7240 (8.89%) | 1423 (9.71%) |  |  | |
| MADD | 6789 (8.34%) | 1129 (7.70%) |  |  | |
| Panic/phobia | 6329 (7.77%) | 1003 (6.85%) |  |  | |
| Adjustment disorder | 4429 (5.44%) | 1320 (9.01%) |  |  | |
| PTSD | 2761 (3.39%) | 1132 (7.73%) |  |  | |
| OCD | 1169 (1.44%) | 550 (3.75%) |  |  | |
| Missing | 13572 | 1605 |  |  | |
| **Psychotropic medication** | | | 0.466 | < 0.01 | |
| Prescribed | 31024 (35.42%) | 5545 (35.72%) |  |  | |
| Not prescribed | 56565 (64.58%) | 9977 (64.28%) |  |  | |
| Missing | 7390 | 736 |  |  | |
| **Ethnicity** |  |  | 0.268 | < 0.01 | |
| White | 56994 (62.92%) | 9789 (63.64%) |  |  | |
| Black | 17654 (19.49%) | 2964 (19.27%) |  |  | |
| Mixed | 6565 (7.25%) | 1111 (7.22%) |  |  | |
| Asian | 6050 (6.68%) | 961 (6.25%) |  |  | |
| Other | 3320 (3.67%) | 557 (3.62%) |  |  | |
| Missing | 4396 | 876 |  |  | |
| **Employment status** |  |  | 0.002 | 0.01 | |
| Employed | 57249 (64.21%) | 10033 (63.39%) |  |  | |
| Unemployed | 19009 (21.32%) | 3572 (22.57%) |  |  | |
| Non-worker ^3^ | 12897 (14.47%) | 2222 (14.04%) |  |  | |
| Missing | 5824 | 431 |  |  | |
| **Disability** ^4^ |  |  | 0.004 | < 0.01 | |
| Yes | 8527 (8.98%) | 1575 (9.69%) |  |  | |
| No | 86452 (91.02%) | 14683 (90.31%) |  |  | |
| **Number of sessions** (including baseline assessment) | | | < 0.001 | 0.96 | |
| Mean (SD) | 4.01 (4.53; 1.00 - 73.00) | 8.35 (4.53; 3.00 - 75.00) |  |  | |
| **Recovered** ^5^ |  |  | < 0.001 | 0.20 | |
| Yes | 20424 (26.72%) | 7028 (51.98%) |  |  | |
| No | 56008 (73.28%) | 6493 (48.02%) |  |  | |
| Missing | 18547 | 2737 |  |  | |
| **Reason for end of treatment** |  |  | < 0.001 | 0.30 | |
| Discharged | 36322 (38.24%) | 13138 (80.81%) |  |  | |
| Dropout | 36821 (38.77%) | 2328 (14.32%) |  |  | |
| Referred | 21836 (22.99%) | 792 (4.87%) |  |  | |
| **Service** |  |  | < 0.001 | 0.13 | |
| 0 | 26143 (27.53%) | 7027 (43.22%) |  |  | |
| 1 | 21573 (22.71%) | 3402 (20.93%) |  |  | |
| 2 | 21579 (22.72%) | 3244 (19.95%) |  |  |  |
| 3 | 25684 (27.04%) | 2585 (15.90%) |  |  | |

*Note:* Percentages were calculated using the available sample for each variable, after excluding missing values. The "Missing" row represents the number of missing values and is omitted if there was no missing data. Effect sizes are Cohen’s *d* for continuous variables and Cramer’s *V* for categorical. *p-*values are from Chi-Square tests for categorical variables and ANOVAs/t-tests for continuous variables. The descriptives of the analytical sample here were created prior to any filtering of sessions, hence differences in e.g., number of sessions and recovery rates to those reported in Table 1 for the cleaned analytical sample. ^1^ Case thresholds were PHQ9 ≥10, GAD7 ≥8. ^2^ GAD = generalised anxiety disorder; PTSD = post-traumatic stress disorder; OCD = obsessive-compulsive disorder; MADD = mixed anxiety and depressive disorder; Panic/phobia = panic disorder, agoraphobia, social phobia, specific phobia; ‘Other’ included somatoform disorder, severe mental illness. Differences in problem descriptor frequencies partly reflect inclusion criteria, as some disorder-specific treatments are more likely to be high-intensity. ^3^ ‘Non-worker’ included homemaker, carer, retired, student. ^4^ No negative responses were recorded therefore the absence of any value was taken as a negative response rather than missing. ^5^ Only calculated for patients who scored above case thresholds on either the PHQ9 or GAD7 at the start of treatment and had an observed score for their final session, otherwise coded as missing. Represents whether the patient recovered within their received sessions (not ‘within the 11 sessions modelled’ as for the analysis sample only descriptives).

# Supplementary Information 3. Latent growth curves of depression and anxiety symptoms

This section contains fit indices and plots of single latent growth curves of different forms.

**Fit indices for latent growth curves of depression and anxiety symptoms during psychological therapy**

| **Depression Symptoms (PHQ9) Latent Growth Curve Form** | **Parameters** | **AIC** | **BIC** | **CFI** | **TLI** | **SRMR** | **RMSEA estimate** |
| --- | --- | --- | --- | --- | --- | --- | --- |
| Quadratic with pairwise residual correlations | 30 | 609627 | 609858 | 0.994 | 0.993 | 0.019 | 0.024 |
| Logarithmic with pairwise residual correlations | 26 | 611250 | 611450 | 0.975 | 0.973 | 0.041 | 0.047 |
| Quadratic | 20 | 611570 | 611724 | 0.973 | 0.974 | 0.030 | 0.046 |
| Linear with pairwise residual correlations | 26 | 611771 | 611971 | 0.969 | 0.967 | 0.109 | 0.052 |
| Logarithmic | 16 | 615385 | 615508 | 0.933 | 0.939 | 0.057 | 0.070 |
| Linear | 16 | 615502 | 615625 | 0.930 | 0.937 | 0.149 | 0.071 |

| **Anxiety Symptoms (GAD7) Latent Growth Curve Form** | **Parameters** | **AIC** | **BIC** | **CFI** | **TLI** | **SRMR** | **RMSEA estimate** |
| --- | --- | --- | --- | --- | --- | --- | --- |
| Quadratic with pairwise residual correlations | 30 | 591154 | 591385 | 0.994 | 0.992 | 0.021 | 0.024 |
| Logarithmic with pairwise residual correlations | 26 | 592934 | 593134 | 0.971 | 0.969 | 0.046 | 0.049 |
| Quadratic | 20 | 593028 | 593181 | 0.971 | 0.972 | 0.034 | 0.046 |
| Linear with pairwise residual correlations | 26 | 593040 | 593240 | 0.969 | 0.967 | 0.115 | 0.050 |
| Linear | 16 | 596403 | 596526 | 0.930 | 0.937 | 0.154 | 0.069 |
| Logarithmic | 16 | 596974 | 597097 | 0.924 | 0.932 | 0.064 | 0.072 |

*Note:* Rows are ordered by BIC, with the optimal model first. AIC = Akaike’s Information Criterion, BIC = Bayesian Information Criterion, CFI = Comparative Fit Index, TLI = Tucker-Lewis Index, SRMR = Standardised Root Mean Square Residual, RMSEA = Root Mean Square Error of Approximation. Lower values indicate superior fit for AIC, BIC, SRMR, RMSEA. Higher values closer to 1 indicate better fit for the CFI and TLI.


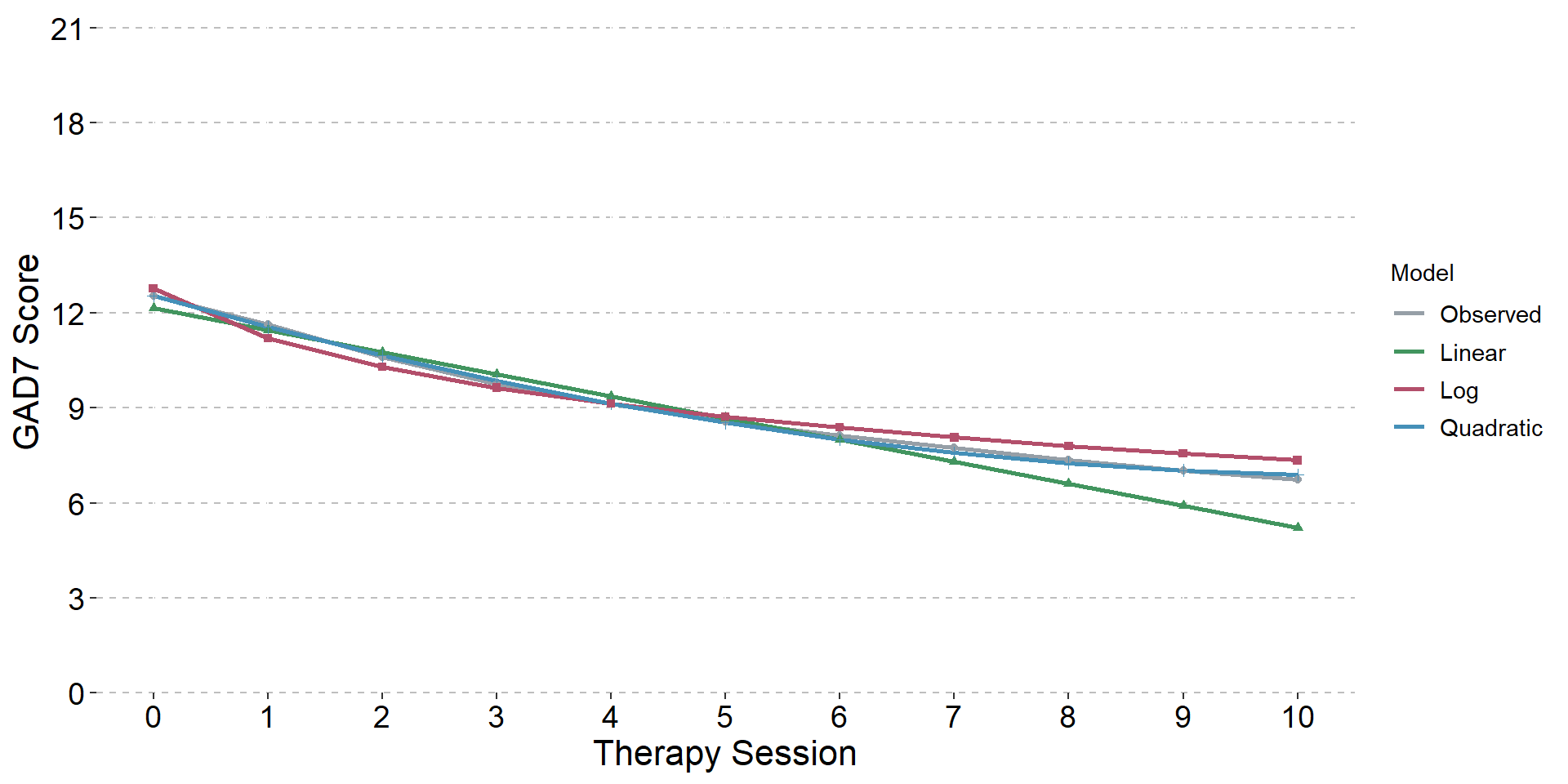

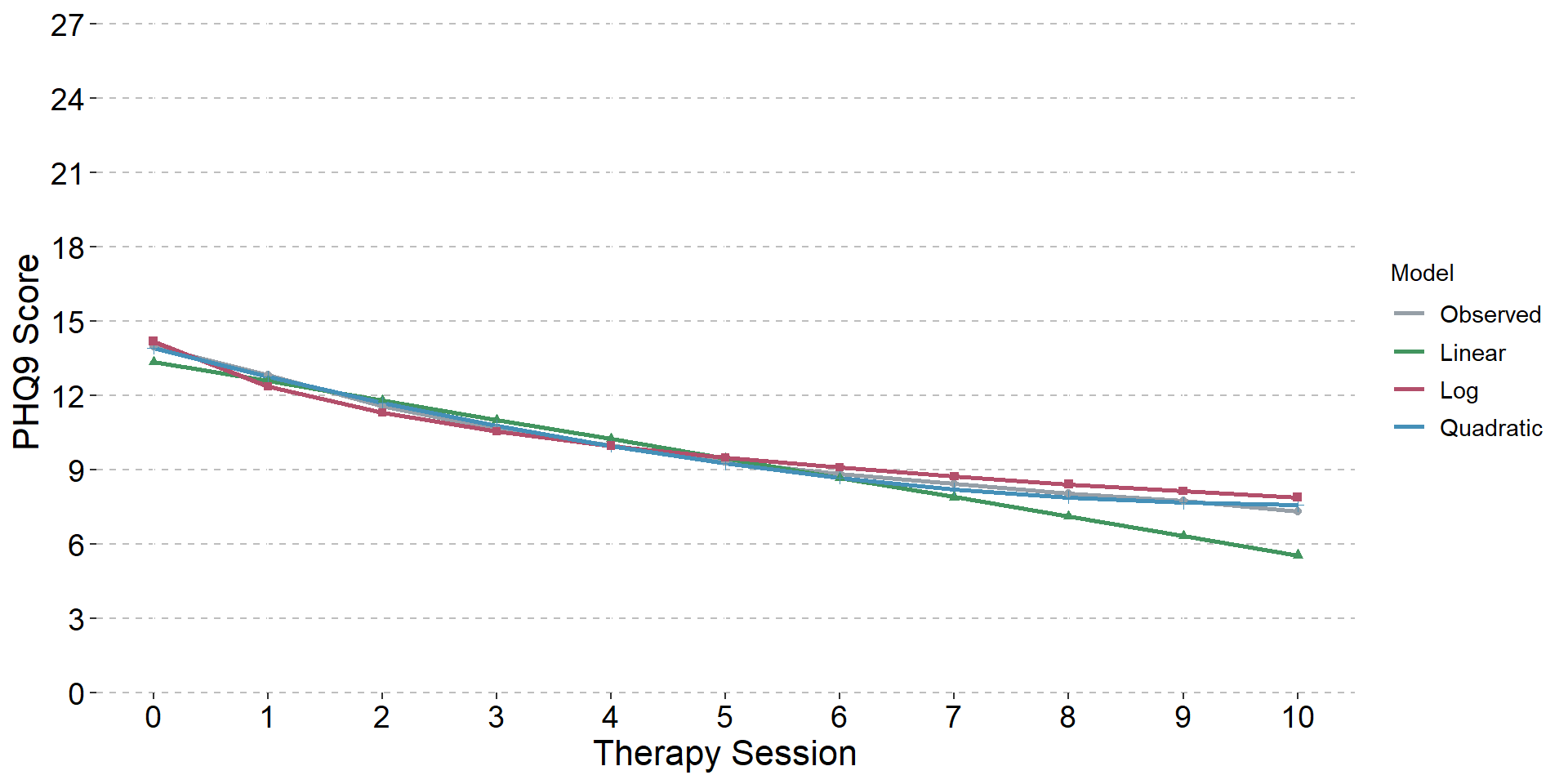
**Latent growth curves of depression (PHQ9; left) and anxiety (GAD7; right) symptoms**

*Note:* The intercept represents the estimated mean outcome score across the whole sample at baseline (session 0). Only trajectories with the residuals correlated are shown as were a better fit than forms with uncorrelated residuals.

# Supplementary Information 4. Latent class growth analysis of depression and anxiety symptoms

This section describes the model selection for latent class growth analysis (LCGA), where the variance within classes is restricted to zero. It includes fit indices for the estimated models, and plots of the selected models. The LCGA of depression symptoms suggested a four-class model. All models had class trajectories that primarily differed in baseline severity, had classes with more than 1% of the sample and entropy values were good for the two- to four-class models (and acceptable for the others). The information criteria continued to decrease up to the six-class model, however, the elbow plot of BIC values showed a plateau around four classes. The VLMR-LRT *p*-values were significant for all models besides the six-class model, indicating that it was a poorer fit of the data than a five class-model. However, BIC values showed a negligible decrease for the five-class model and therefore the four-class model was selected. There was some indication of a moderate-severe plateau class (grey diamonds), moderate-severe with gradual improvement (pink squares), slightly faster improvement to plateau (blue triangles) and a class with mild symptoms that showed small improvement (green circles).

The anxiety symptoms LCGA also suggested a four-class model. All models had class trajectories that primarily differed in baseline severity, with more than 1% of the sample in each class and good entropy for the two- to three-class models (and acceptable for the four- to six-class). The VLMR-LRT *p*-values were significant for all models. The information criteria decreased up to the six-class model, however, the elbow plot of BIC values showed a plateau around four classes. The four-class model indicated a moderate-severe plateau class (grey diamonds), moderate-severe, gradual improvement (pink squares), moderate-severe, fast improvement (blue triangles) and mild, small improvement class (green circles).

#

**Fit indices for latent class growth analysis of depression and anxiety symptoms during psychological therapy (N = 16,258)**

| **Depression symptoms (PHQ9) LCGA** | **Parameters** | **AIC** | **BIC** | **Entropy** | **VLMR LRT**  ***p*-value** | **Individuals per class (%)** |
| --- | --- | --- | --- | --- | --- | --- |
| Growth Curve | 24 | 664047 | 664232 | NA | NA | 100 |
| Two Class | 28 | 630926 | 631142 | 0.847 | < 0.001 | 62.1, 37.9 |
| Three Class | 32 | 618738 | 618984 | 0.826 | < 0.001 | 17.8, 41.8, 40.4 |
| Four Class | 36 | 613685 | 613962 | 0.799 | < 0.001 | 27.9, 10.3, 37.1, 24.7 |
| Five Class | 40 | 611898 | 612206 | 0.763 | 0.004 | 32.9, 6.5, 24.2, 21.4, 15.0 |
| Six Class | 44 | 610804 | 611142 | 0.740 | 0.085 | 18.3, 30.8, 8.2, 17.6, 17.8, 7.2 |

| **Anxiety symptoms (GAD7) LCGA** | **Parameters** | **AIC** | **BIC** | **Entropy** | **VLMR LRT**  ***p*-value** | **Individuals per class (%)** |
| --- | --- | --- | --- | --- | --- | --- |
| Growth Curve | 24 | 638901 | 639086 | NA | NA | 100 |
| Two Class | 28 | 607070 | 607285 | 0.839 | < 0.001 | 58.7, 41.3 |
| Three Class | 32 | 597165 | 597411 | 0.801 | < 0.001 | 23.3, 40.5, 36.2 |
| Four Class | 36 | 593260 | 593537 | 0.766 | < 0.001 | 13.9, 25.4, 35.6, 25.0 |
| Five Class | 40 | 592043 | 592351 | 0.720 | < 0.001 | 15.7, 10.8, 19.1, 24.7, 29.7 |
| Six Class | 44 | 590846 | 591185 | 0.707 | 0.0002 | 10.7, 29.1, 18.9, 15.0, 12.0, 14.3 |

*Note*: A quadratic form with correlations between the residuals of adjacent time points was specified for all classes. AIC = Akaike’s Information Criterion, BIC = Bayesian Information Criterion, VLMR LRT = Vuong-Lo-Mendell-Rubin Likelihood Ratio Test. Number of parameters in the growth curve (single class) differs from that in the latent growth curve (24 compared with 30) as the slope and intercept variance and covariance are fixed here. Individuals per class is based on a patient’s highest posterior probability of belonging to a class.


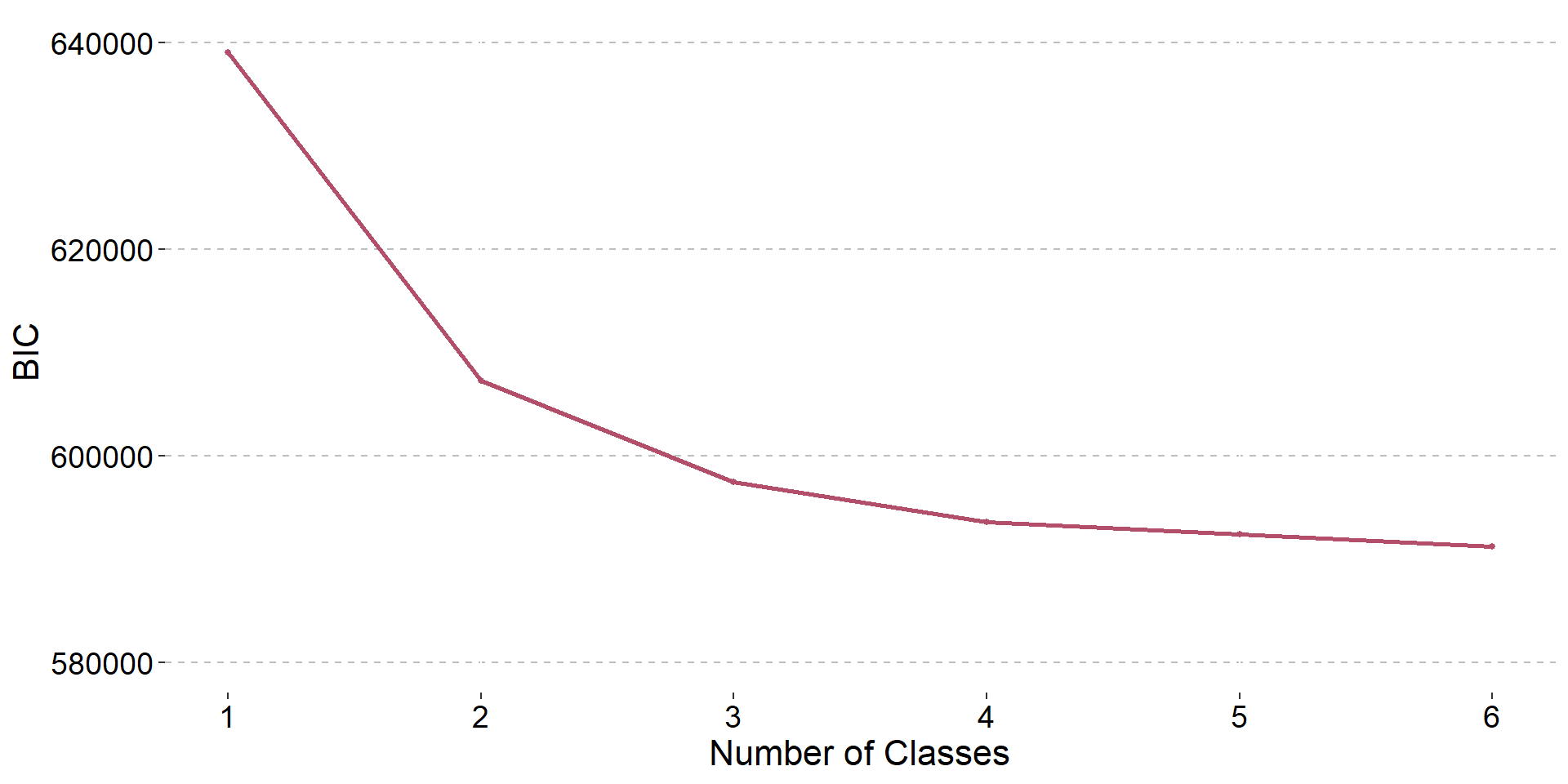
**Elbow plots of Bayesian Information Criterion values for latent class growth analysis of depression (PHQ9; left) and anxiety (GAD7; right) symptoms**


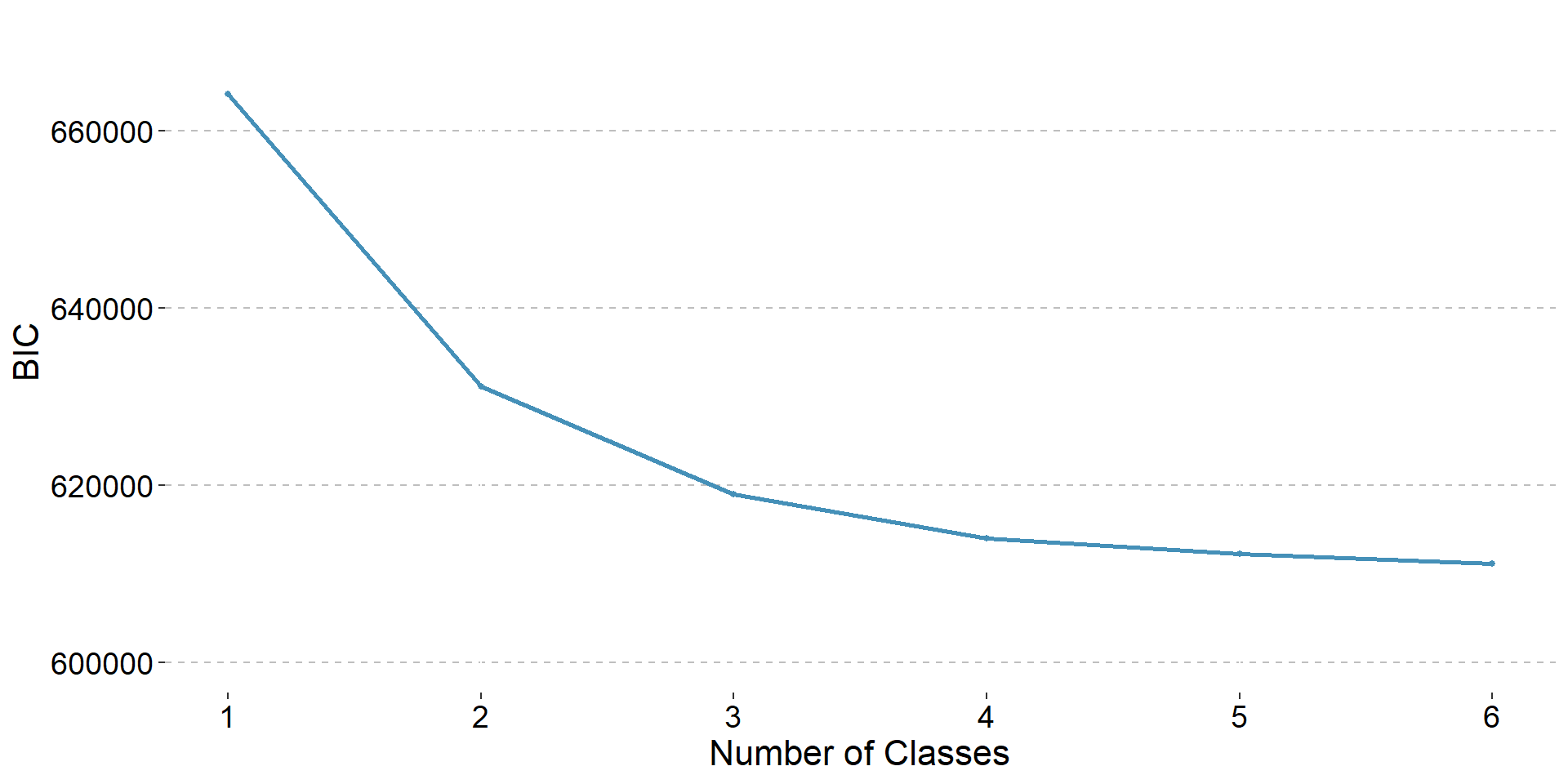


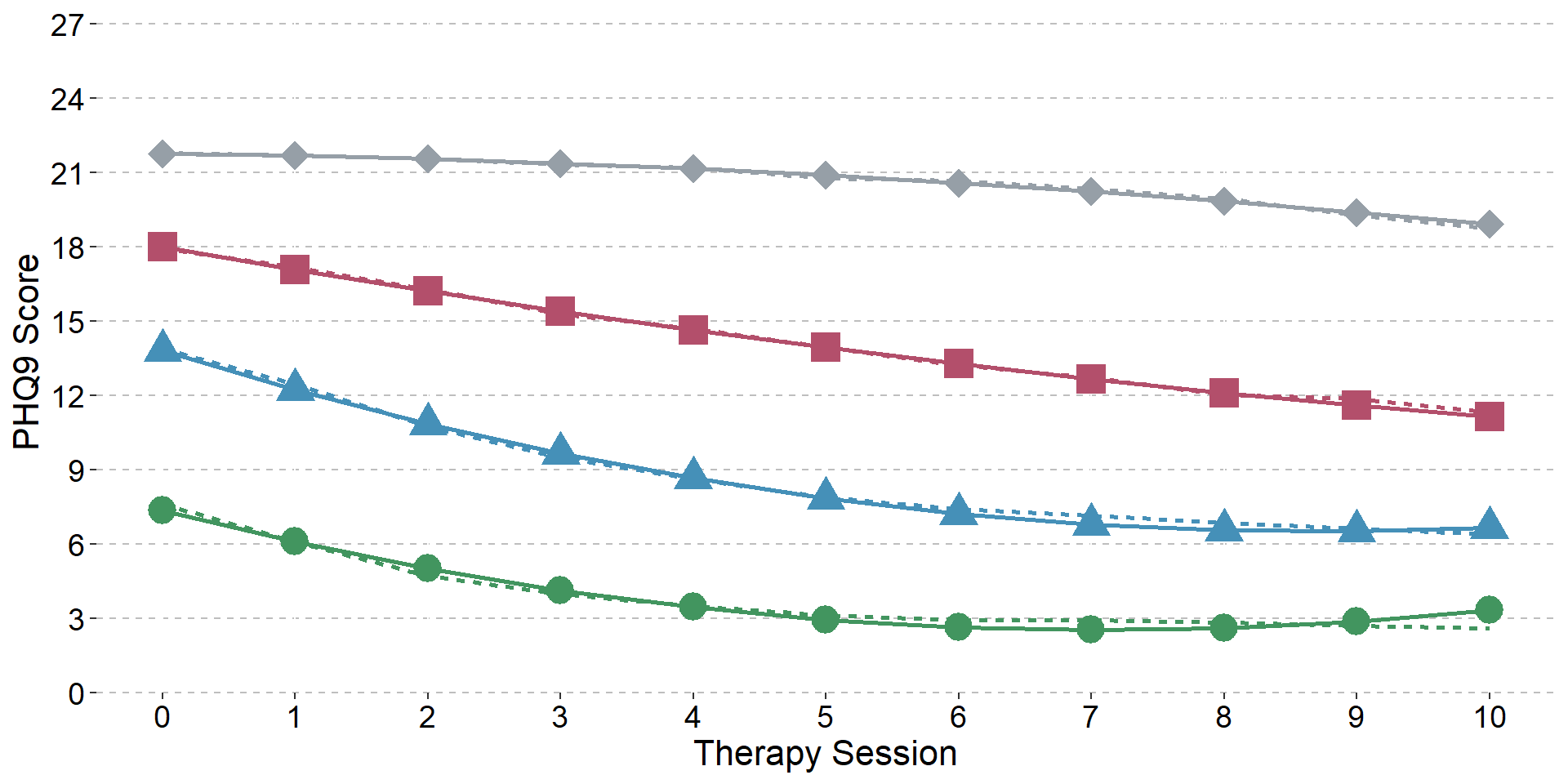

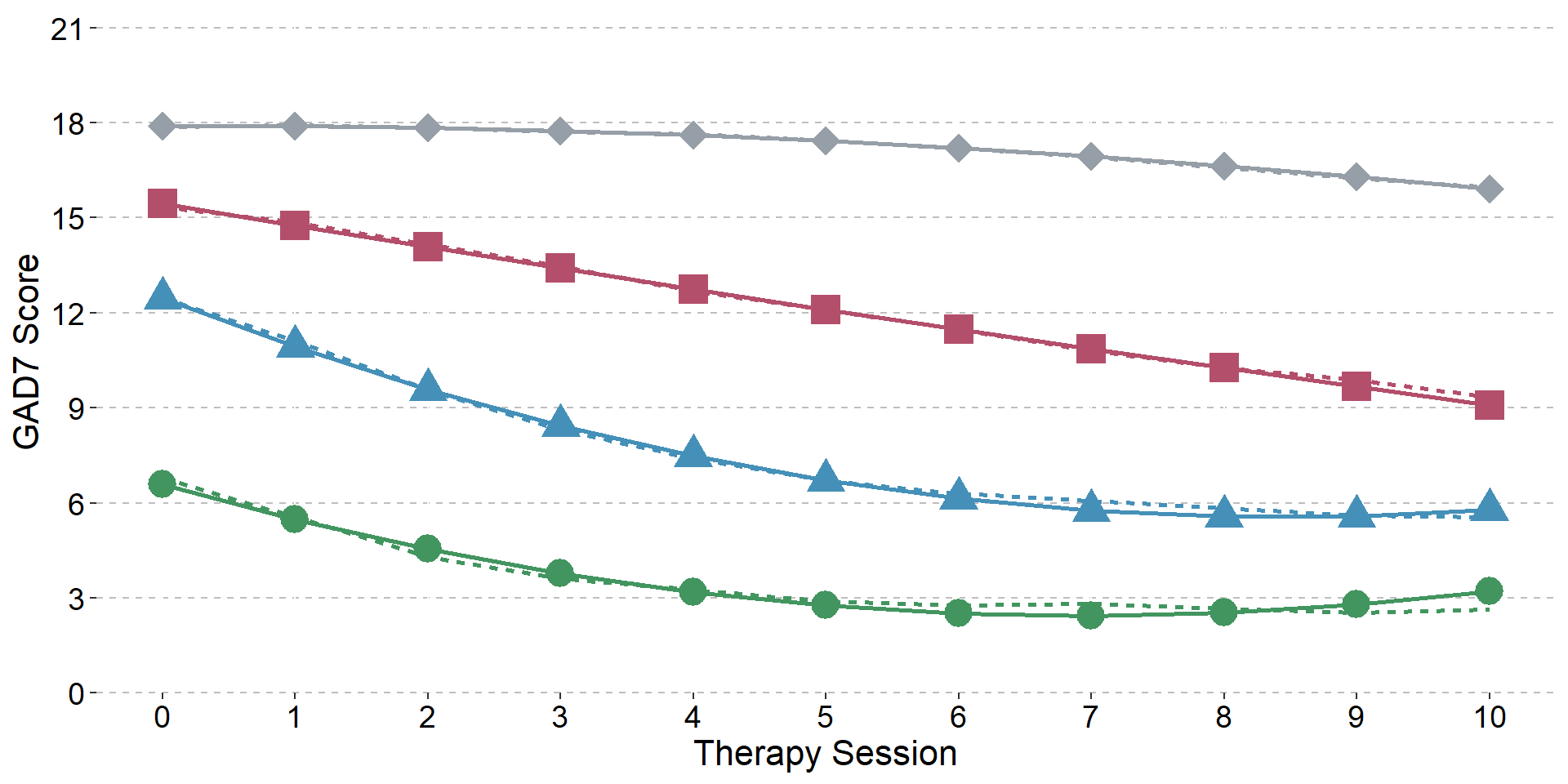
**Four-class latent class growth models of depression (PHQ9; top) and anxiety (GAD7; bottom) symptoms during psychological therapy (N = 16,258)**

6,035 (37.12%)

4,531 (27.87%)

1,672 (10.29%)

4,019 (24.72%)

5,793 (35.63%)

4,068 (25.02%)

2,260 (13.90%)

4,136 (25.44%)

# Supplementary Information 5. Selection of depression symptoms growth mixture model

This section describes the model selection for the growth mixture model of depression symptoms, including fit indices and trajectory plots. Information criteria continued to decrease up to a six-class model and the VLMR LRT *p*-value did not become non-significant, however, six classes were unrealistically high compared with existing studies (hence chosen as the upper number of classes to test). The BIC values elbow-plot suggested a four-class model, consistent with previous literature, and this was selected as the optimal model. Entropy of the four-class model was medium (0.60).

#

**Fit indices for growth mixture models of depression symptoms during psychological therapy (N = 16,258)**

| **Depression symptoms (PHQ9) GMM** | **Parameters** | **AIC** | **BIC** | **Entropy** | **VLMR LRT**  ***p*-value** | **Individuals per class (%)** |
| --- | --- | --- | --- | --- | --- | --- |
| Growth Curve | 25 | 613935 | 614128 | NA | NA | 100 |
| Two Class | 29 | 610886 | 611109 | 0.538 | < 0.001 | 79.1, 20.9 |
| Three Class | 33 | 608940 | 609194 | 0.593 | < 0.001 | 52.3, 22.7, 24.9 |
| Four Class | 37 | 607920 | 608204 | 0.600 | < 0.001 | 13.5, 52.5, 17.6, 16.4 |
| Five Class | 41 | 607573 | 607888 | 0.606 | 0.0017 | 10.1, 20.9, 16.4, 2.2, 50.5 |
| Six Class | 45 | 607186 | 607532 | 0.632 | 0.0001 | 2.9, 9.4, 44.4, 25.5, 2.5, 15.3 |

*Note*: A quadratic form with correlations between the residuals of adjacent time points was specified for all classes. AIC = Akaike’s Information Criterion, BIC = Bayesian Information Criterion, VLMR LRT = Vuong-Lo-Mendell-Rubin Likelihood Ratio Test. Individuals per class is based on a patient’s highest posterior probability of belonging to a class.

**Elbow plot of Bayesian Information Criterion values for growth mixture models of depression symptoms**

#
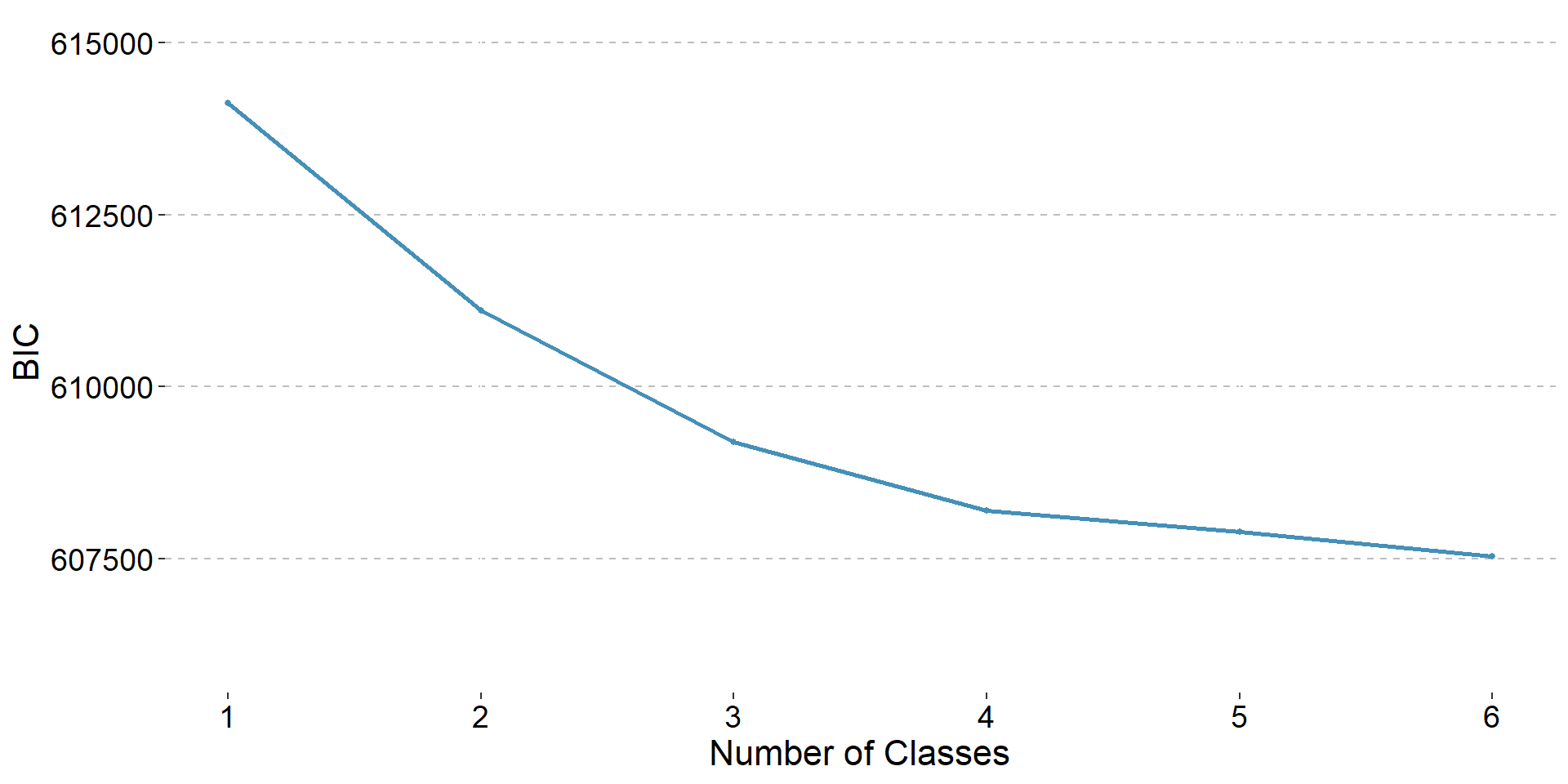


#

#

#

#

#

#

#

#


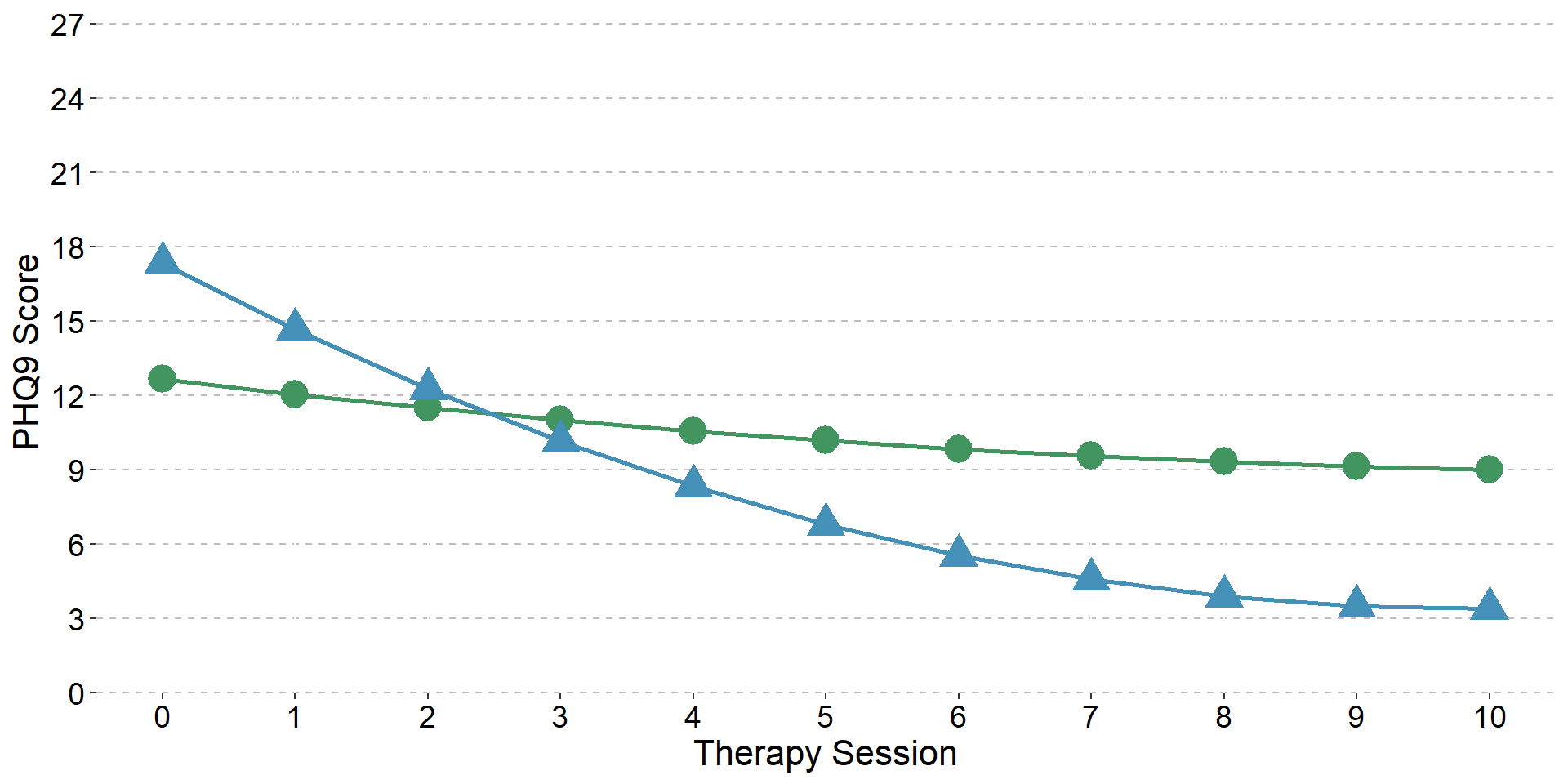

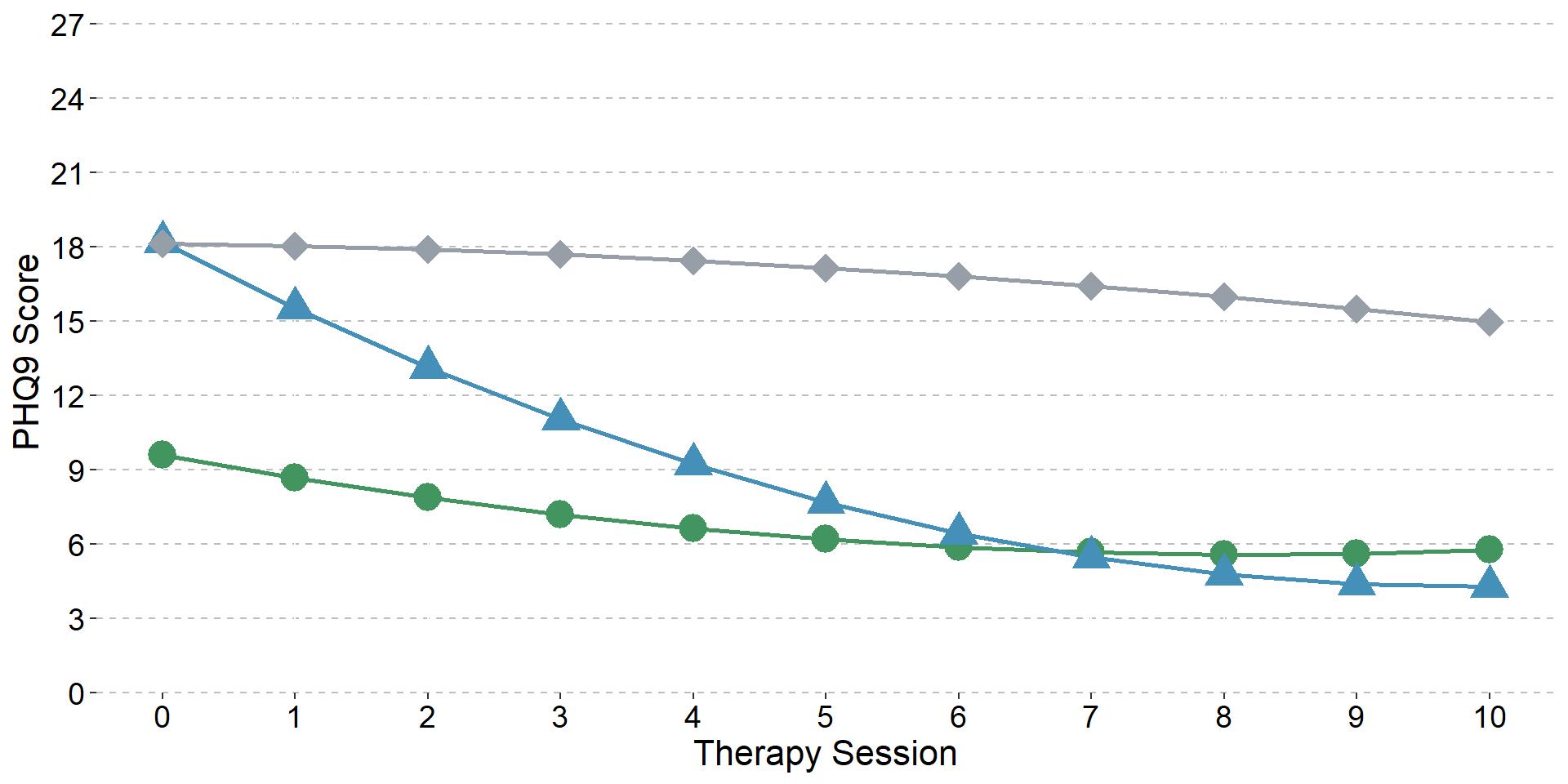

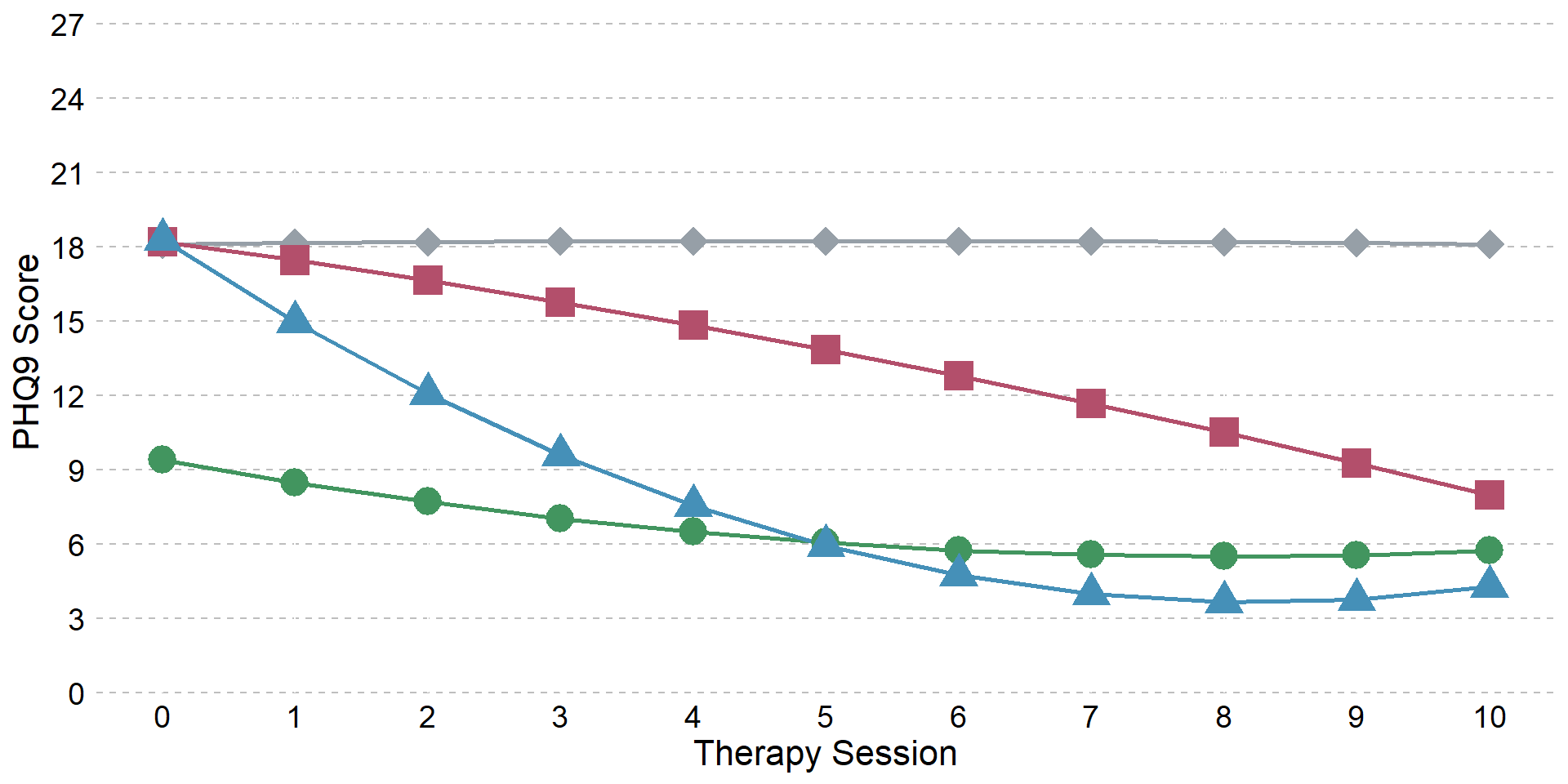

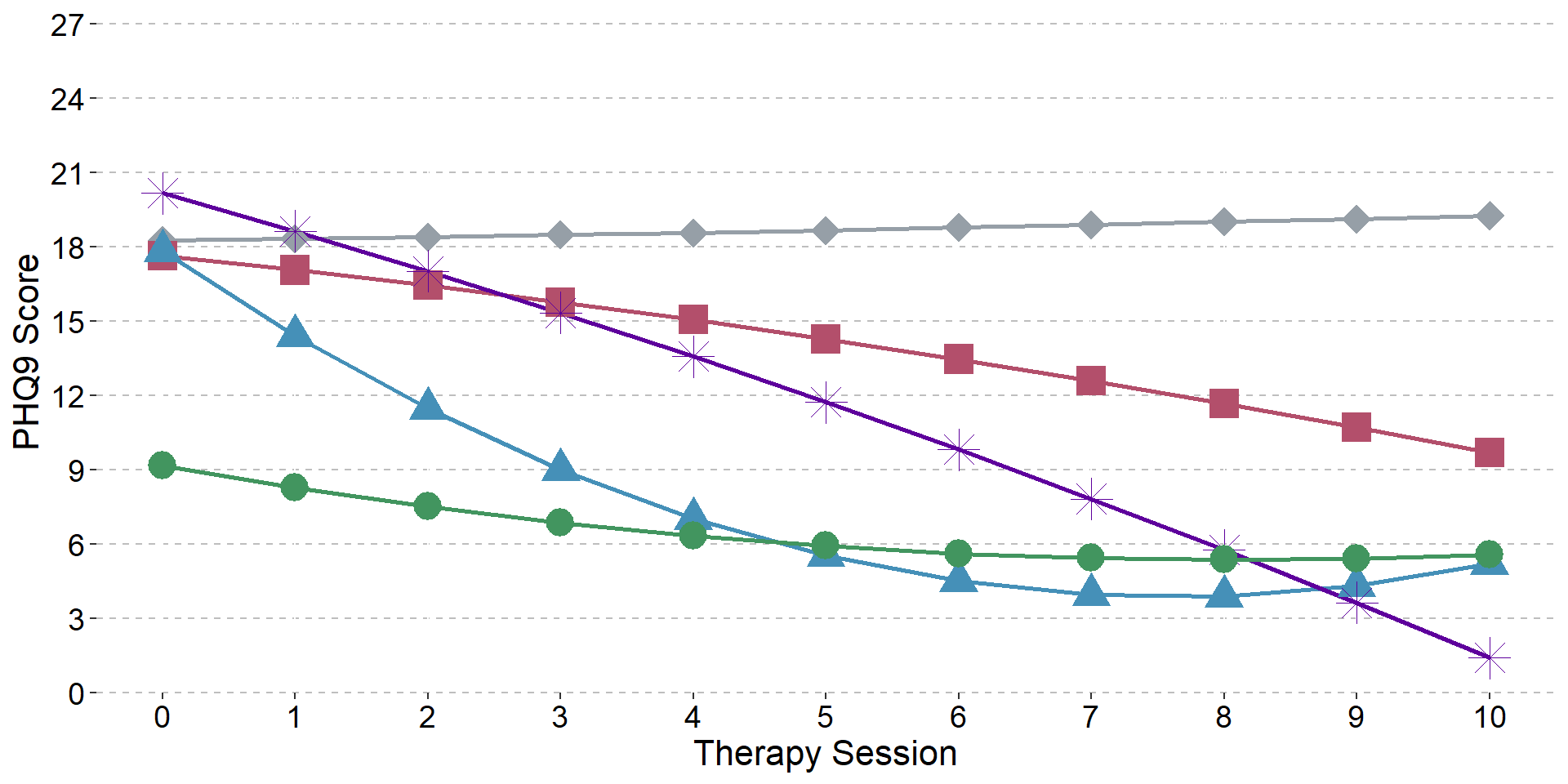

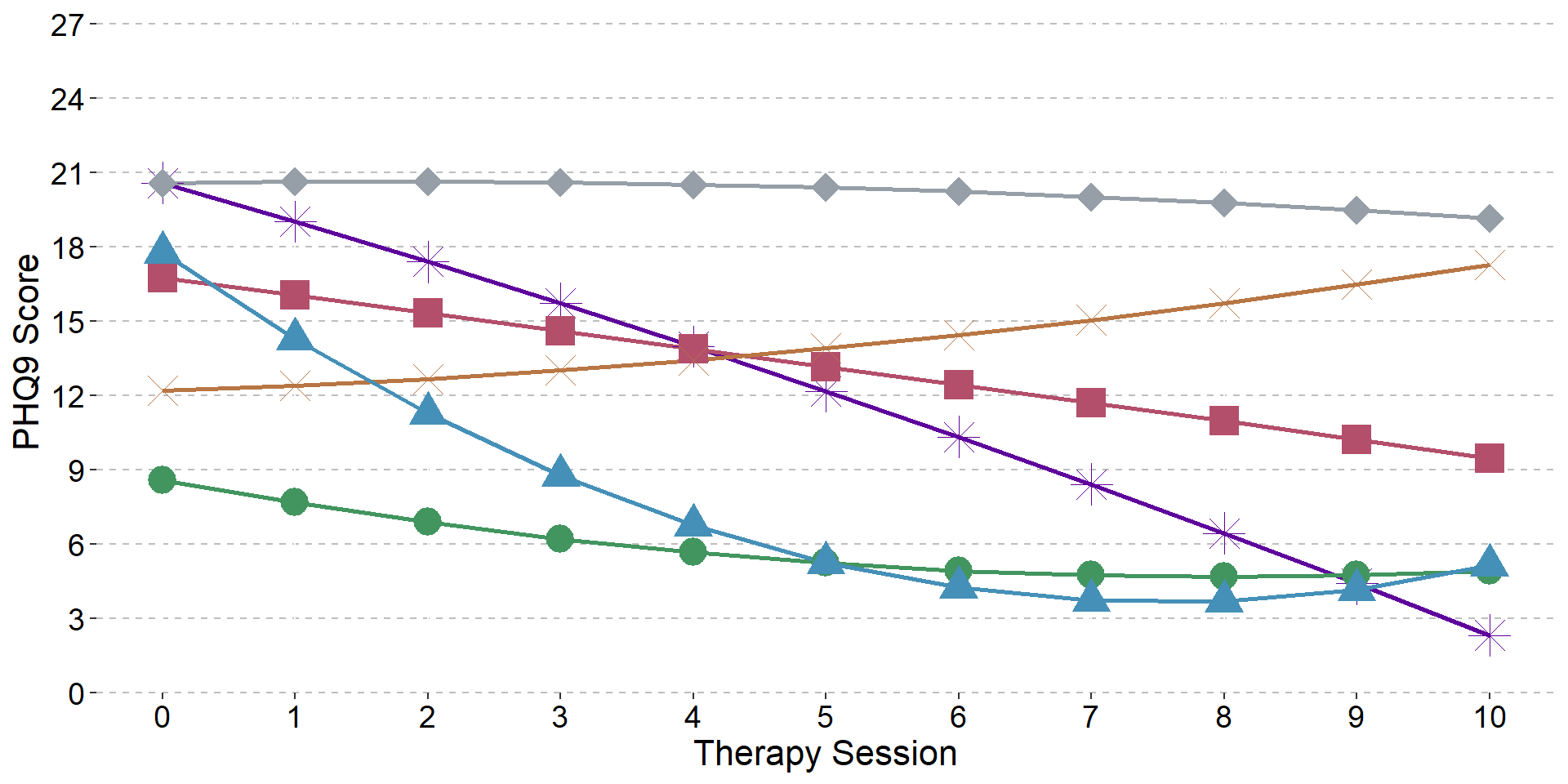
**Two- to six-class growth mixture models of depression symptoms during psychological therapy**

# Supplementary Information 6. Descriptives of four-class growth mixture model of depression symptoms

**Descriptives of the growth factors for the four-class growth mixture model of depression symptoms**

| **Class** | **Parameter** | **Factor** | **Estimate** | **SE** | **Est SE** | ***p*-value** |
| --- | --- | --- | --- | --- | --- | --- |
| Moderate-severe plateau | Means | Intercept | 18.11 | 0.18 | 102.677 | 0 |
|  |  | Linear | 0.06 | 0.06 | 1.047 | 0.295 |
|  |  | Quadratic | -0.01 | 0.01 | -0.851 | 0.395 |
|  | Variances | Intercept | 11.6 | 0.24 | 48.404 | 0 |
|  |  | Linear | 0 | 0 | 999 | 999 |
|  |  | Quadratic | 0 | 0 | 999 | 999 |
| Moderate-severe,  gradual improvement | Means | Intercept | 18.22 | 0.18 | 103.533 | 0 |
|  |  | Linear | -0.72 | 0.10 | -7.17 | 0 |
|  |  | Quadratic | -0.03 | 0.01 | -3.854 | 0 |
|  | Variances | Intercept | 11.6 | 0.24 | 48.404 | 0 |
|  |  | Linear | 0 | 0 | 999 | 999 |
|  |  | Quadratic | 0 | 0 | 999 | 999 |
| Moderate-severe, fast improvement | Means | Intercept | 18.31 | 0.14 | 127 | 0 |
|  |  | Linear | -3.54 | 0.10 | -34.759 | 0 |
|  |  | Quadratic | 0.21 | 0.01 | 16.972 | 0 |
|  | Variances | Intercept | 11.6 | 0.24 | 48.404 | 0 |
|  |  | Linear | 0 | 0 | 999 | 999 |
|  |  | Quadratic | 0 | 0 | 999 | 999 |
| Mild, small improvement | Means | Intercept | 9.41 | 0.11 | 85.924 | 0 |
|  |  | Linear | -0.97 | 0.03 | -31.445 | 0 |
|  |  | Quadratic | 0.06 | 0.00 | 20.742 | 0 |
|  | Variances | Intercept | 11.6 | 0.24 | 48.404 | 0 |
|  |  | Linear | 0 | 0 | 999 | 999 |
|  |  | Quadratic | 0 | 0 | 999 | 999 |

**Model estimated depression symptom scores (PHQ9) per therapy session and trajectory class**

|  | **Session** | | | | | | | | | | |
| --- | --- | --- | --- | --- | --- | --- | --- | --- | --- | --- | --- |
| **Class** | **0** | **1** | **2** | **3** | **4** | **5** | **6** | **7** | **8** | **9** | **10** |
| Moderate-severe plateau | 18.11 | 18.16 | 18.20 | 18.23 | 18.25 | 18.26 | 18.25 | 18.24 | 18.21 | 18.17 | 18.13 |
| Moderate-severe, gradual improvement | 18.22 | 17.47 | 16.65 | 15.78 | 14.85 | 13.86 | 12.80 | 11.69 | 10.52 | 9.29 | 7.99 |
| Moderate-severe, fast improvement | 18.31 | 14.99 | 12.09 | 9.62 | 7.57 | 5.96 | 4.77 | 4.01 | 3.68 | 3.77 | 4.29 |
| Mild, small improvement | 9.41 | 8.50 | 7.71 | 7.04 | 6.50 | 6.07 | 5.76 | 5.58 | 5.51 | 5.57 | 5.74 |

**Descriptives of patients in each class of the four-class growth mixture model of depression symptoms (assigned to their most likely trajectory class)**

|  | **Moderate-severe plateau (N=2200)** | **Moderate-severe, gradual improvement (N=2857)** | **Moderate-severe, fast improvement  (N=2670)** | **Mild, small improvement (N=8530)** |
| --- | --- | --- | --- | --- |
| **Depression symptoms (PHQ9)** |  |  |  |  |
| Mean (SD) | 18.55 (5.04) | 19.13 (3.80) | 19.24 (3.58) | 9.43 (4.34) |
| Range | 0.00 - 27.00 | 0.00 - 27.00 | 6.00 - 27.00 | 0.00 - 25.00 |
| Missing | 26 | 32 | 14 | 80 |
| **Anxiety symptoms (GAD7)** |  |  |  |  |
| Mean (SD) | 15.75 (4.30) | 15.81 (3.86) | 15.44 (4.14) | 9.69 (4.82) |
| Range | 0.00 - 21.00 | 0.00 - 21.00 | 0.00 - 21.00 | 0.00 - 21.00 |
| Missing | 26 | 32 | 15 | 80 |
| **Case on PHQ9 and/or GAD7** ^1^ |  |  |  |  |
| Yes | 2128 (97.88%) | 2816 (99.68%) | 2653 (99.92%) | 6138 (72.65%) |
| No | 46 (2.12%) | 9 (0.32%) | 2 (0.08%) | 2311 (27.35%) |
| Missing | 26 | 32 | 15 | 81 |
| **Functional impairment score (WSAS)** | |  |  |  |
| Mean (SD) | 23.11 (9.42) | 22.98 (8.46) | 20.80 (8.70) | 13.74 (7.72) |
| Range | 0.00 - 40.00 | 0.00 - 40.00 | 0.00 - 40.00 | 0.00 - 40.00 |
| Missing | 740 | 1032 | 1107 | 2567 |
| **Psychotropic medication** | | |  |  |
| Prescribed | 1095 (52.54%) | 1301 (48.06%) | 1015 (39.26%) | 2134 (26.20%) |
| Not prescribed | 989 (47.46%) | 1406 (51.94%) | 1570 (60.74%) | 6012 (73.80%) |
| Missing | 116 | 150 | 85 | 384 |
| **Employment status** |  |  |  |  |
| Employed | 956 (44.61%) | 1531 (55.01%) | 1658 (63.35%) | 5888 (71.08%) |
| Unemployed | 884 (41.25%) | 875 (31.44%) | 612 (23.39%) | 1201 (14.50%) |
| Non-worker ^2^ | 303 (14.14%) | 377 (13.55%) | 347 (13.26%) | 1195 (14.43%) |
| Missing | 57 | 74 | 53 | 246 |
| **Disability** ^3^ |  |  |  |  |
| Yes | 367 (16.68%) | 344 (12.04%) | 254 (9.51%) | 610 (7.15%) |
| No | 1833 (83.32%) | 2513 (87.96%) | 2416 (90.49%) | 7920 (92.85%) |
| **Ethnicity** |  |  |  |  |
| White | 1156 (56.53%) | 1566 (58.43%) | 1464 (57.71%) | 5603 (69.01%) |
| Black | 443 (21.66%) | 555 (20.71%) | 626 (24.67%) | 1339 (16.49%) |
| Mixed | 157 (7.68%) | 205 (7.65%) | 209 (8.24%) | 540 (6.65%) |
| Asian | 189 (9.24%) | 231 (8.62%) | 152 (5.99%) | 389 (4.79%) |
| Other | 100 (4.89%) | 123 (4.59%) | 86 (3.39%) | 248 (3.05%) |
| Missing | 155 | 177 | 133 | 411 |
| **Problem descriptor** ^4^ | |  |  |  |
| Depression | 968 (49.54%) | 1396 (54.42%) | 1362 (55.55%) | 2976 (38.74%) |
| GAD | 133 (6.81%) | 170 (6.63%) | 171 (6.97%) | 919 (11.96%) |
| Other | 128 (6.55%) | 169 (6.59%) | 198 (8.08%) | 928 (12.08%) |
| MADD | 167 (8.55%) | 201 (7.84%) | 202 (8.24%) | 559 (7.28%) |
| Panic/phobia | 103 (5.27%) | 122 (4.76%) | 105 (4.28%) | 673 (8.76%) |
| Adjustment disorder | 134 (6.86%) | 146 (5.69%) | 188 (7.67%) | 852 (11.09%) |
| PTSD | 255 (13.05%) | 277 (10.80%) | 175 (7.14%) | 425 (5.53%) |
| OCD | 66 (3.38%) | 84 (3.27%) | 51 (2.08%) | 349 (4.54%) |
| Missing | 246 | 292 | 218 | 849 |
| **Age (years)** |  |  |  |  |
| Mean (SD) | 39.13 (13.36) | 37.47 (13.44) | 37.58 (13.31) | 37.16 (13.31) |
| Range | 17.00 - 90.00 | 17.00 - 91.00 | 17.00 - 89.00 | 16.00 - 94.00 |
| **Gender** |  |  |  |  |
| Female | 1453 (66.11%) | 1923 (67.43%) | 1808 (67.74%) | 5794 (68.00%) |
| Male | 745 (33.89%) | 929 (32.57%) | 861 (32.26%) | 2727 (32.00%) |
| Missing | 2 | 5 | 1 | 9 |
| **Number of sessions** (including baseline assessment) | | |  |  |
| Mean (SD) | 7.48 (2.79) | 7.13 (2.86) | 6.35 (2.38) | 6.68 (2.62) |
| Range | 3.00 - 11.00 | 3.00 - 11.00 | 3.00 - 11.00 | 3.00 - 11.00 |
| **Recovered** ^5^ |  |  |  |  |
| Yes | 12 (0.57%) | 522 (18.70%) | 1790 (67.83%) | 3579 (58.78%) |
| No | 2101 (99.43%) | 2270 (81.30%) | 849 (32.17%) | 2510 (41.22%) |
| Missing | 87 | 65 | 31 | 2441 |
| **Reason for end of treatment** |  |  |  |  |
| Discharged | 1532 (69.64%) | 2115 (74.03%) | 2187 (81.91%) | 7303 (85.62%) |
| Dropout | 416 (18.91%) | 581 (20.34%) | 393 (14.72%) | 938 (11.00%) |
| Referred | 252 (11.45%) | 161 (5.64%) | 90 (3.37%) | 289 (3.39%) |
| **Service** |  |  |  |  |
| 0 | 880 (40.00%) | 1210 (42.35%) | 1377 (51.57%) | 3560 (41.74%) |
| 1 | 532 (24.18%) | 633 (22.16%) | 567 (21.24%) | 1670 (19.58%) |
| 2 | 371 (16.86%) | 563 (19.71%) | 424 (15.88%) | 1886 (22.11%) |
| 3 | 417 (18.95%) | 451 (15.79%) | 302 (11.31%) | 1414 (16.58%) |

*Note:* Percentages were calculated using the available sample for each variable, after excluding missing values. The "Missing" row represents the number of missing values and was omitted if there was no missing data. ^1^ Case thresholds: PHQ9 ≥10, GAD7 ≥8. ^2^ ‘Non-worker’ included homemaker, carer, retired, student. ^3^ No negative responses were recorded, therefore the absence of any value was taken as a negative response rather than missing. ^4^ GAD = generalised anxiety disorder; PTSD = post-traumatic stress disorder; OCD = obsessive-compulsive disorder; MADD = mixed anxiety and depressive disorder; Panic/phobia = panic disorder, agoraphobia, social phobia, specific phobia; ‘Other’ included somatoform disorder, severe mental illness. ^5^ Calculated for patients who scored above the case threshold on either/both the PHQ9 or GAD7 at the start of treatment and had an observed score for their final session, otherwise coded as missing. Represents whether the patient reached recovery within the 11 sessions modelled; if a patient received more sessions and then recovered, they would appear unrecovered here.

# Supplementary Information 7. Selection of anxiety symptoms growth mixture model

This section describes the model selection for the growth mixture model of anxiety symptoms, including fit indices and trajectory plots. As in the depression model, the VLMR LRT failed to reach non-significance and the information criteria decreased up to six classes. A four-class model was supported by the BIC elbow-plot and was chosen as the best-fitting model. Entropy in the four-class model was low (0.59).

**Fit indices for growth mixture models of anxiety symptoms during psychological therapy (N = 16,258)**

| **Anxiety symptoms (GAD7) GMM** | **Parameters** | **AIC** | **BIC** | **Entropy** | **VLMR LRT**  **p-value** | | **Individuals per class (%)** |
| --- | --- | --- | --- | --- | --- | --- | --- |
| Growth Curve | 25 | 595485 | 595677 | NA | | NA | 100 |
| Two Class | 29 | 592459 | 592682 | 0.453 | | < 0.001 | 29.6, 70.4 |
| Three Class | 33 | 590113 | 590367 | 0.602 | | < 0.001 | 31.3, 40.5, 28.2 |
| Four Class | 37 | 588890 | 589175 | 0.591 | | < 0.001 | 24.8, 21.8, 11.9, 41.5 |
| Five Class | 41 | 588523 | 588838 | 0.635 | | < 0.001 | 11.7, 1.7, 40.7, 22.3, 23.6 |
| Six Class | 45 | 588228 | 588574 | 0.609 | | 0.0002 | 34.0, 6.3, 17.5, 3.0, 19.2, 19.9 |

*Note:* A quadratic form with correlations between the residuals of adjacent time points was specified for all classes. AIC = Akaike’s Information Criterion, BIC = Bayesian Information Criterion, VLMR LRT = Vuong-Lo-Mendell-Rubin Likelihood Ratio Test. Individuals per class is based on a patient’s highest posterior probability of belonging to a class.

#

**Elbow plot of Bayesian Information Criterion values for growth mixture models of anxiety symptoms**

#
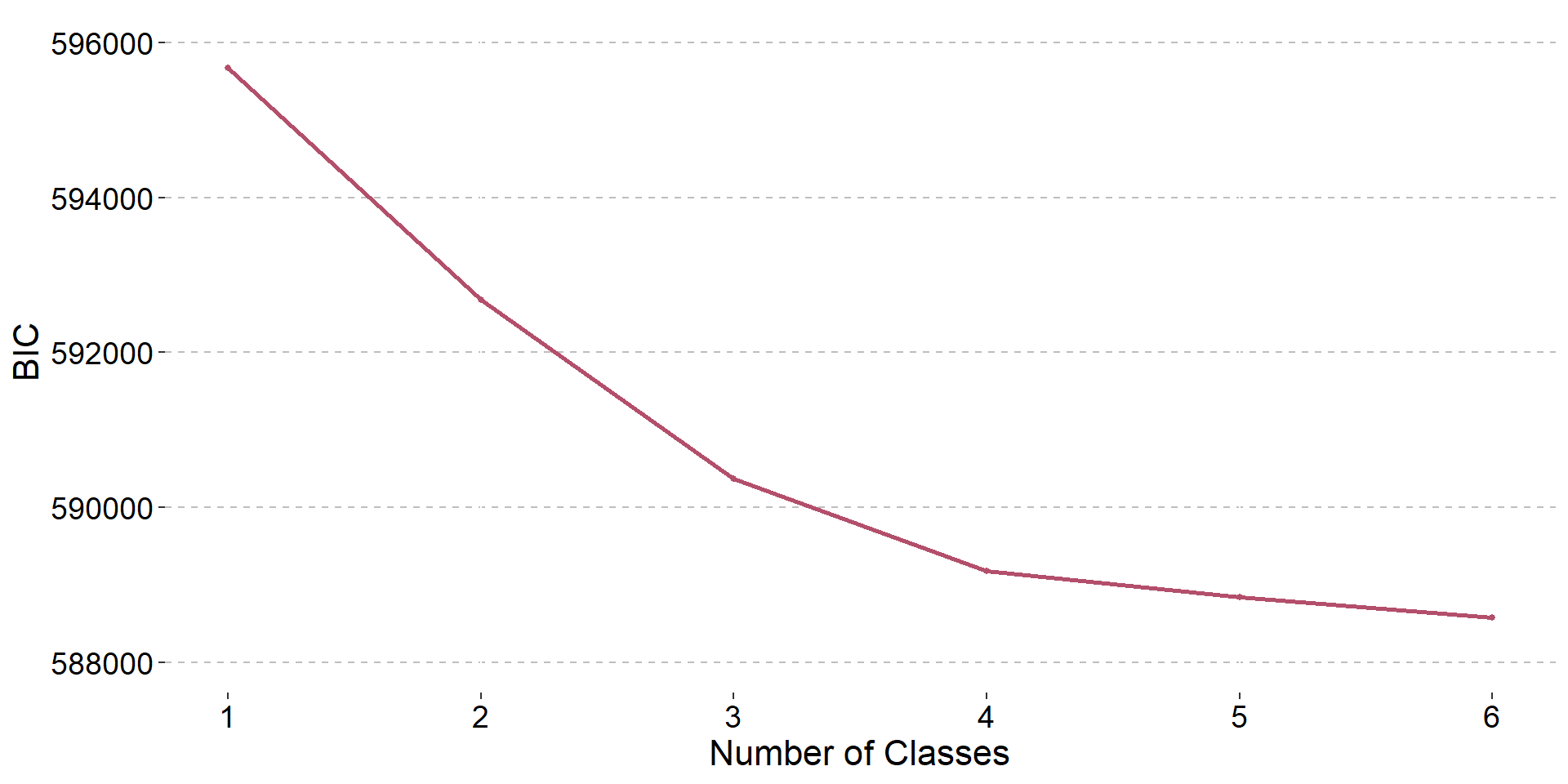


**
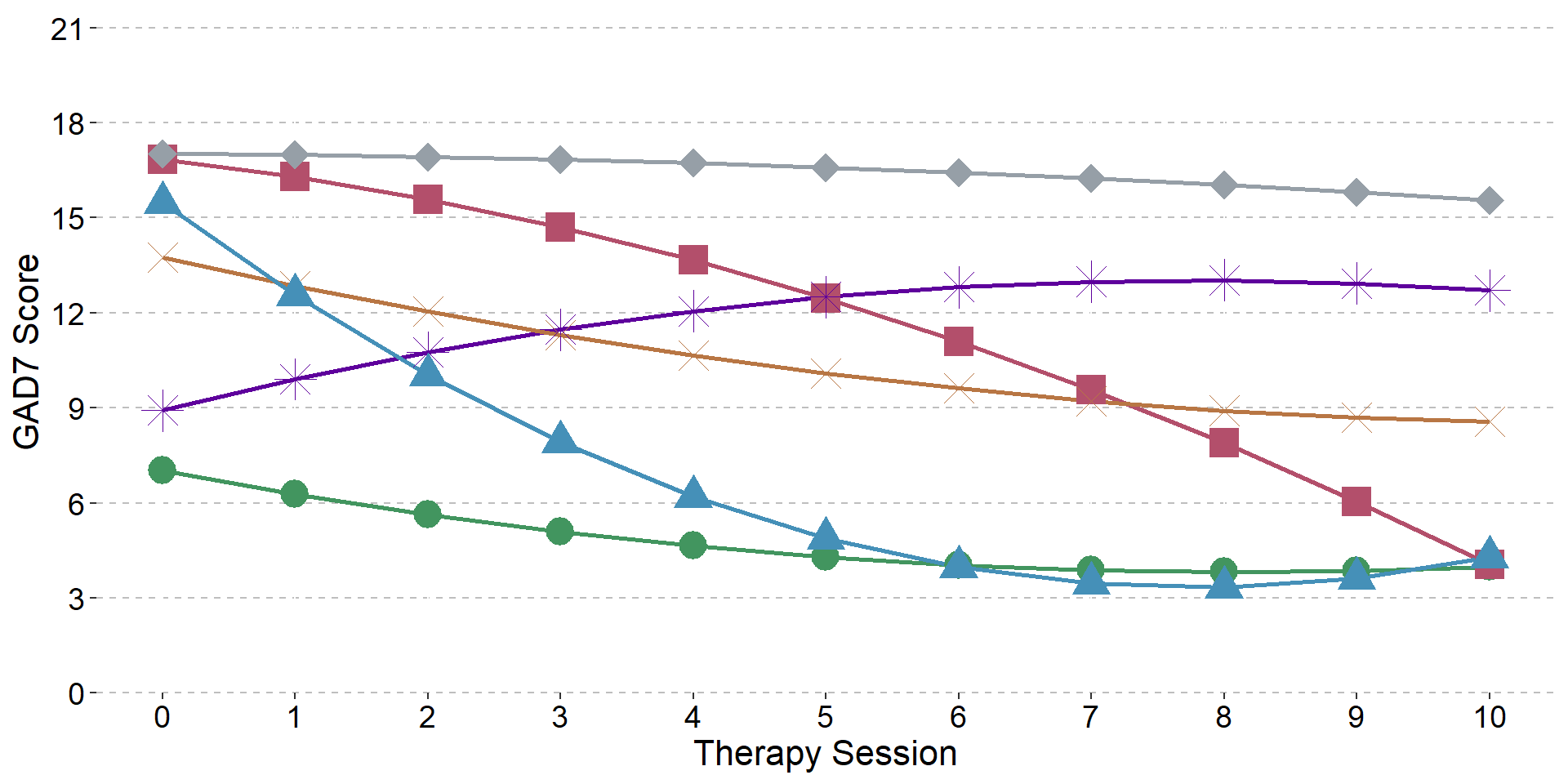
**
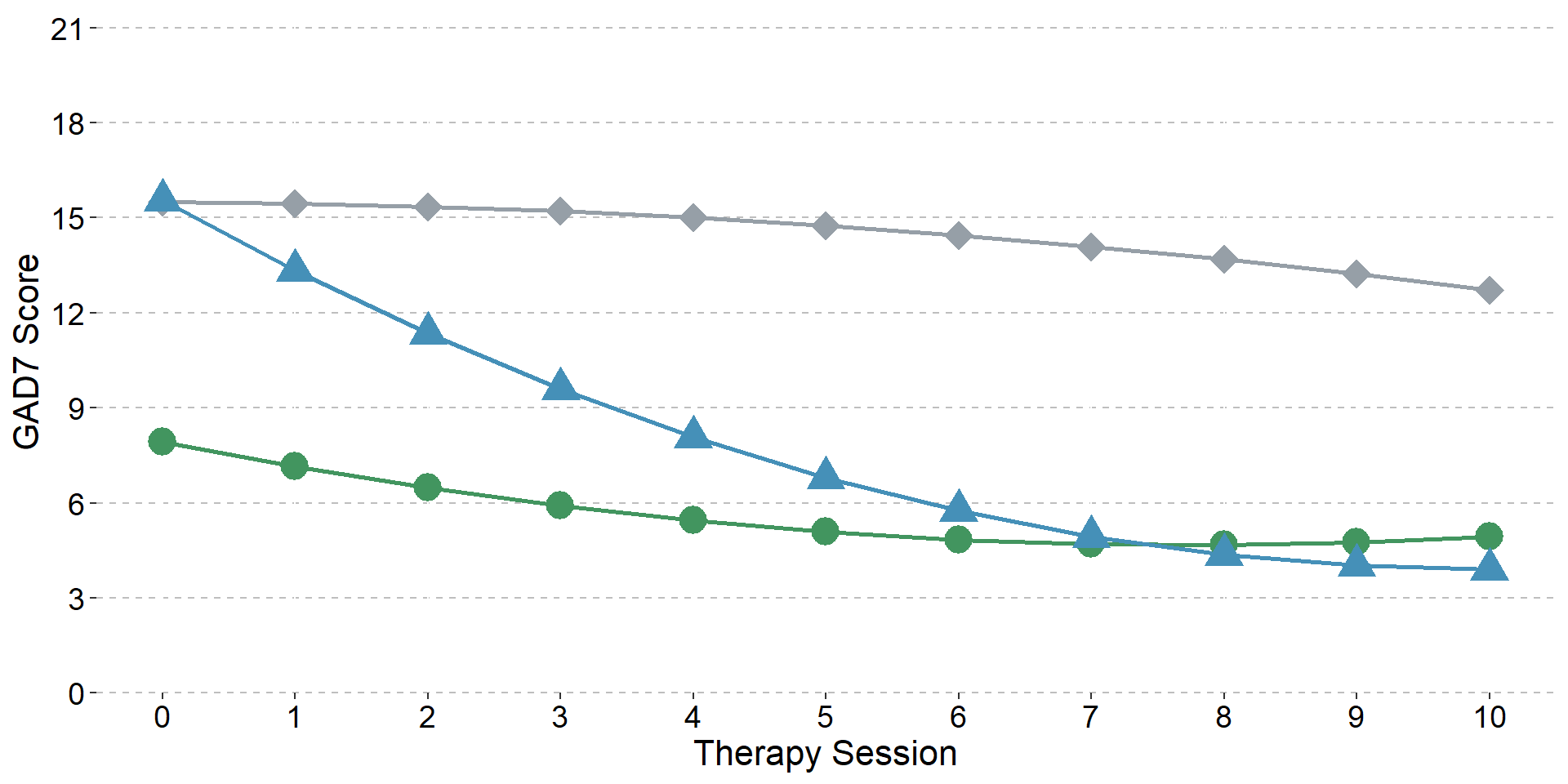

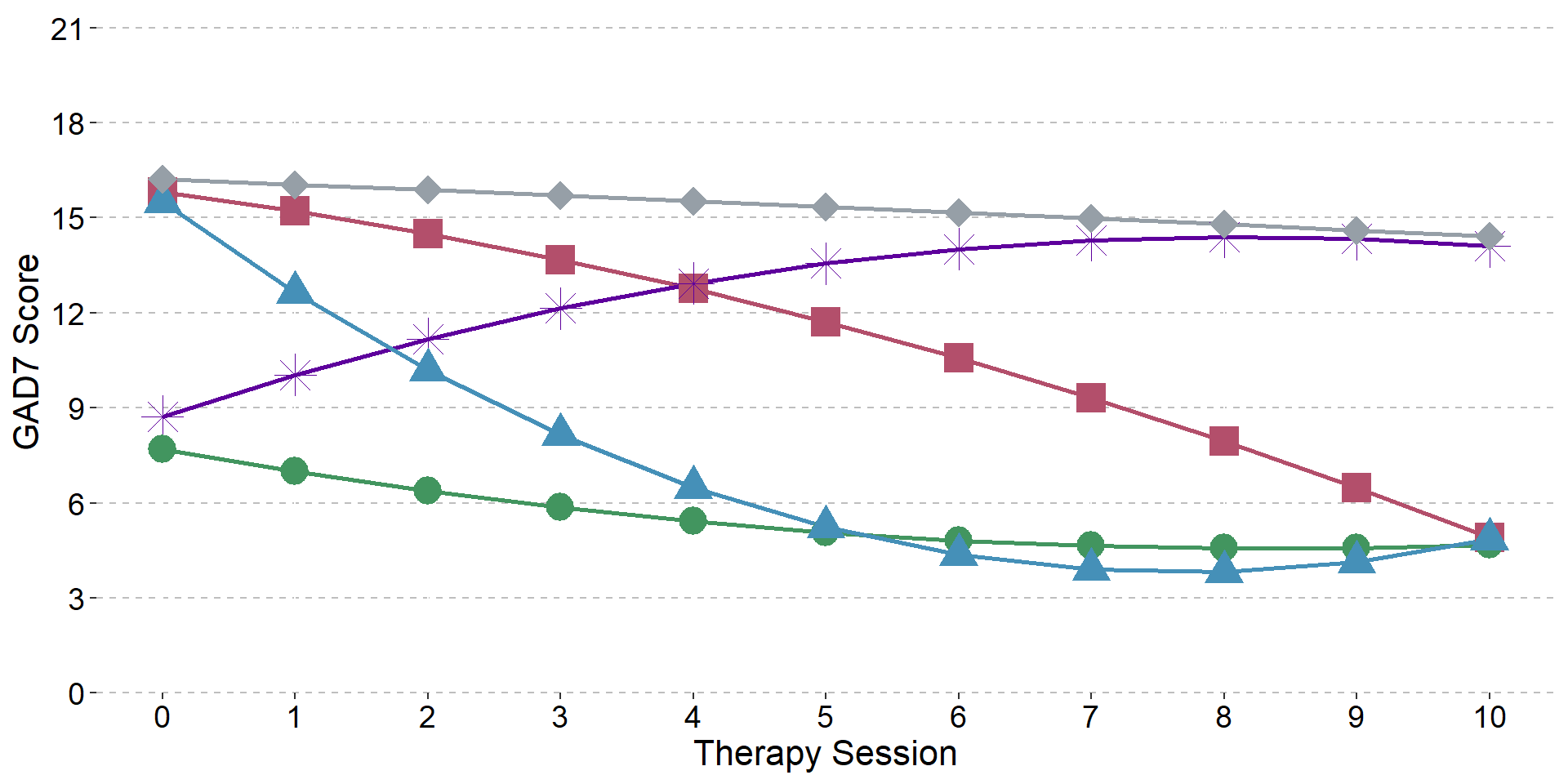

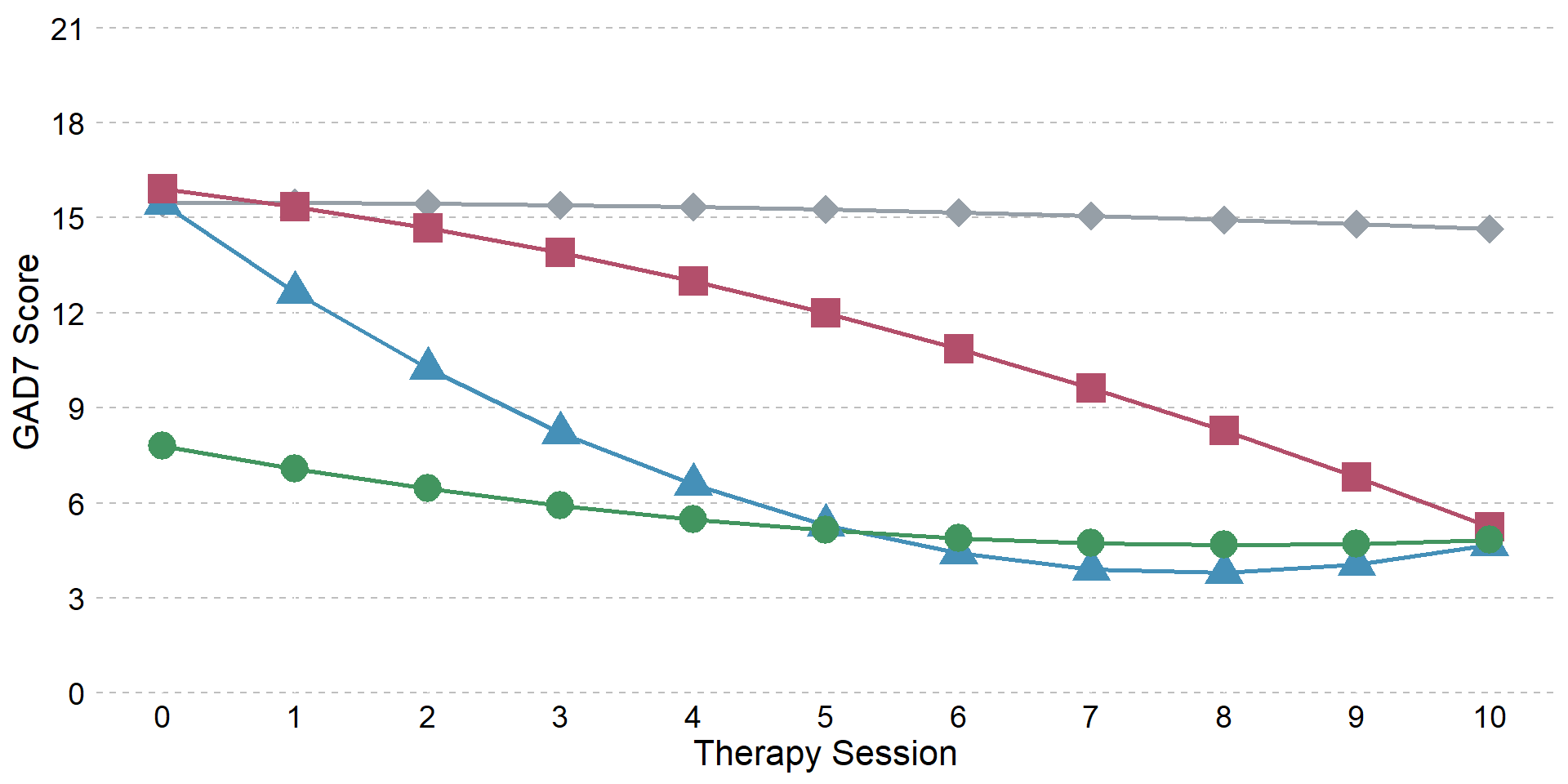
**
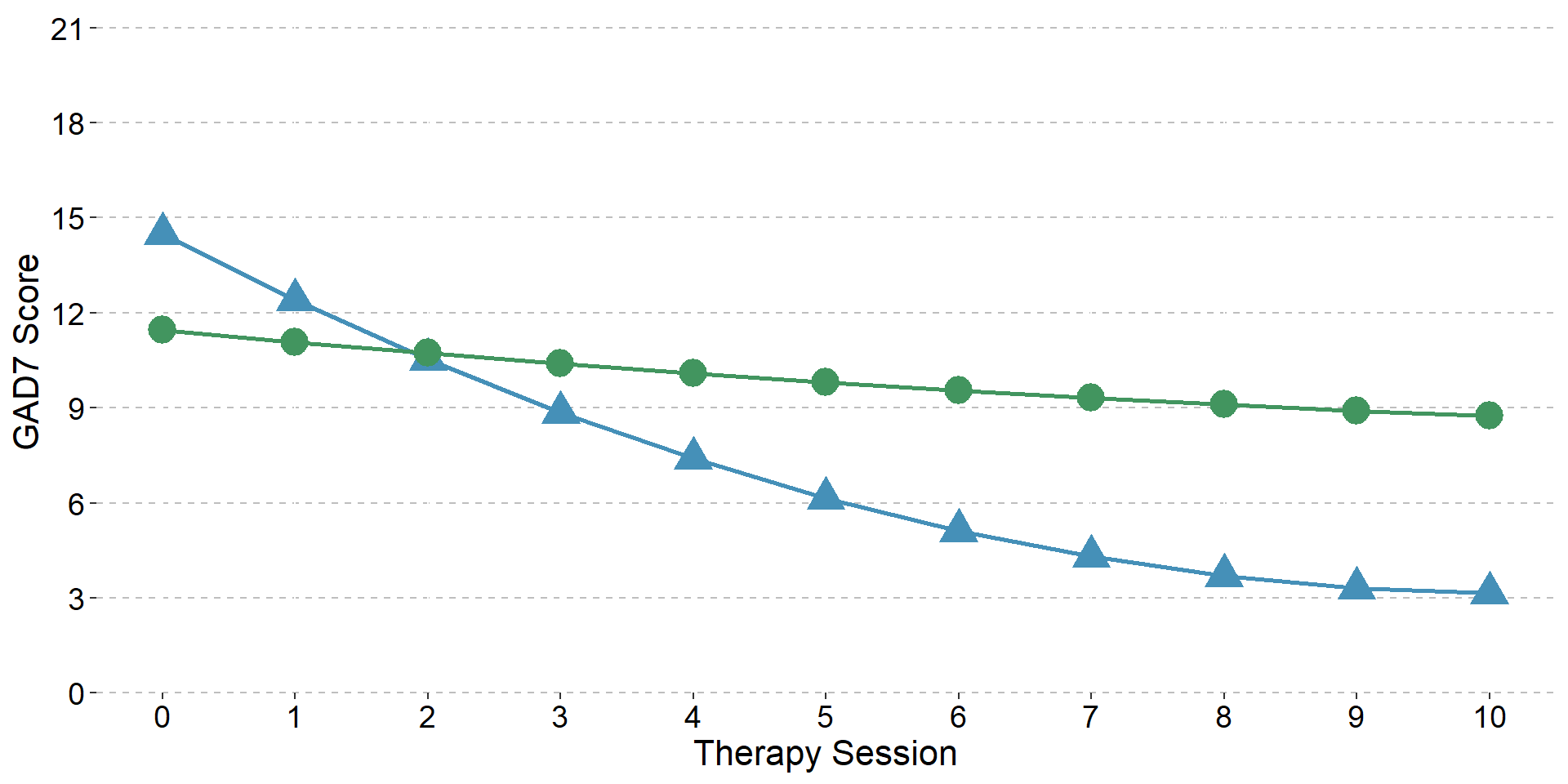
Two- to six-class growth mixture models of anxiety symptoms during psychological therapy**

# Supplementary Information 8. Descriptives of four-class growth mixture model of anxiety symptoms

**Descriptives of the growth factors for the four-class growth mixture model of anxiety symptoms**

| **Class** | **Parameter** | **Factor** | **Estimate** | **SE** | **Est SE** | ***p*-value** |
| --- | --- | --- | --- | --- | --- | --- |
| Moderate-severe plateau | Means | Intercept | 15.475 | 0.094 | 164.421 | 0 |
|  |  | Linear | -0.003 | 0.033 | -0.08 | 0.936 |
|  |  | Quadratic | -0.008 | 0.004 | -2.263 | 0.024 |
|  | Variances | Intercept | 6.811 | 0.125 | 54.584 | 0 |
|  |  | Linear | 0 | 0 | 999 | 999 |
|  |  | Quadratic | 0 | 0 | 999 | 999 |
| Moderate-severe,  gradual improvement | Means | Intercept | 15.908 | 0.12 | 132.867 | 0 |
|  |  | Linear | -0.504 | 0.078 | -6.436 | 0 |
|  |  | Quadratic | -0.056 | 0.008 | -7.128 | 0 |
|  | Variances | Intercept | 6.811 | 0.125 | 54.584 | 0 |
|  |  | Linear | 0 | 0 | 999 | 999 |
|  |  | Quadratic | 0 | 0 | 999 | 999 |
| Moderate-severe,  fast improvement | Means | Intercept | 15.436 | 0.114 | 134.932 | 0 |
|  |  | Linear | -2.977 | 0.06 | -49.832 | 0 |
|  |  | Quadratic | 0.19 | 0.007 | 28.151 | 0 |
|  | Variances | Intercept | 6.811 | 0.125 | 54.584 | 0 |
|  |  | Linear | 0 | 0 | 999 | 999 |
|  |  | Quadratic | 0 | 0 | 999 | 999 |
| Mild, small improvement | Means | Intercept | 7.797 | 0.095 | 82.011 | 0 |
|  |  | Linear | -0.765 | 0.031 | -24.642 | 0 |
|  |  | Quadratic | 0.047 | 0.003 | 15.784 | 0 |
|  | Variances | Intercept | 6.811 | 0.125 | 54.584 | 0 |
|  |  | Linear | 0 | 0 | 999 | 999 |
|  |  | Quadratic | 0 | 0 | 999 | 999 |

**Model estimated anxiety symptom scores (GAD7) per psychological therapy session and trajectory class**

|  | **Session** | | | | | | | | | | |
| --- | --- | --- | --- | --- | --- | --- | --- | --- | --- | --- | --- |
| **Class** | **0** | **1** | **2** | **3** | **4** | **5** | **6** | **7** | **8** | **9** | **10** |
| Moderate-severe plateau | 15.47 | 15.46 | 15.44 | 15.39 | 15.34 | 15.26 | 15.17 | 15.06 | 14.94 | 14.80 | 14.65 |
| Moderate-severe,  gradual improvement | 15.91 | 15.35 | 14.68 | 13.89 | 12.99 | 11.98 | 10.86 | 9.63 | 8.28 | 6.82 | 5.25 |
| Moderate-severe,  fast improvement | 15.44 | 12.65 | 10.24 | 8.22 | 6.57 | 5.30 | 4.42 | 3.91 | 3.79 | 4.05 | 4.68 |
| Mild, small improvement | 7.80 | 7.08 | 6.45 | 5.92 | 5.49 | 5.14 | 4.89 | 4.73 | 4.67 | 4.70 | 4.82 |

**Descriptives of patients in each class of the four-class growth mixture model of anxiety symptoms (assigned to their most likely trajectory class)**

|  | **Moderate-severe plateau (N=4035)** | **Moderate-severe, gradual improvement (N=1931)** | **Moderate-severe, fast improvement (N=3537)** | **Mild, small improvement (N=6754)** |
| --- | --- | --- | --- | --- |
| **Anxiety symptoms (GAD7)** |  |  |  |  |
| Mean (SD) | 15.74 (3.87) | 16.31 (3.27) | 16.07 (2.91) | 7.67 (3.61) |
| Range | 0.00 - 21.00 | 0.00 - 21.00 | 7.00 - 21.00 | 0.00 - 20.00 |
| Missing | 47 | 21 | 18 | 67 |
| **Depression symptoms (PHQ9)** |  |  |  |  |
| Mean (SD) | 17.92 (5.33) | 17.37 (5.21) | 16.02 (5.26) | 9.59 (5.02) |
| Range | 0.00 - 27.00 | 0.00 - 27.00 | 0.00 - 27.00 | 0.00 - 27.00 |
| Missing | 47 | 21 | 18 | 66 |
| **Case on PHQ9 and/or GAD7** ^1^ |  |  |  |  |
| Yes | 3934 (98.65%) | 1895 (99.21%) | 3515 (99.89%) | 4391 (65.67%) |
| No | 54 (1.35%) | 15 (0.79%) | 4 (0.11%) | 2295 (34.33%) |
| Missing | 47 | 21 | 18 | 68 |
| **Functional impairment score (WSAS)** | |  |  |  |
| Mean (SD) | 22.21 (9.39) | 21.06 (8.60) | 18.84 (8.53) | 13.44 (7.91) |
| Range | 0.00 - 40.00 | 0.00 - 40.00 | 0.00 - 40.00 | 0.00 - 40.00 |
| Missing | 1405 | 653 | 1311 | 2077 |
| **Psychotropic medication** | | |  |  |
| Prescribed | 1831 (47.79%) | 766 (41.38%) | 1186 (34.96%) | 1762 (27.33%) |
| Not prescribed | 2000 (52.21%) | 1085 (58.62%) | 2206 (65.04%) | 4686 (72.67%) |
| Missing | 204 | 80 | 145 | 306 |
| **Employment status** |  |  |  |  |
| Employed | 1958 (49.90%) | 1162 (61.55%) | 2309 (66.75%) | 4604 (70.23%) |
| Unemployed | 1413 (36.01%) | 468 (24.79%) | 678 (19.60%) | 1013 (15.45%) |
| Non-worker ^2^ | 553 (14.09%) | 258 (13.67%) | 472 (13.65%) | 939 (14.32%) |
| Missing | 111 | 43 | 78 | 198 |
| **Disability** ^3^ |  |  |  |  |
| Yes | 570 (14.13%) | 190 (9.84%) | 289 (8.17%) | 526 (7.79%) |
| No | 3465 (85.87%) | 1741 (90.16%) | 3248 (91.83%) | 6228 (92.21%) |
| **Ethnicity** |  |  |  |  |
| White | 2225 (58.55%) | 1149 (62.75%) | 2066 (61.40%) | 4349 (68.11%) |
| Black | 806 (21.21%) | 325 (17.75%) | 720 (21.40%) | 1112 (17.42%) |
| Mixed | 275 (7.24%) | 138 (7.54%) | 271 (8.05%) | 427 (6.69%) |
| Asian | 320 (8.42%) | 145 (7.92%) | 198 (5.88%) | 298 (4.67%) |
| Other | 174 (4.58%) | 74 (4.04%) | 110 (3.27%) | 199 (3.12%) |
| Missing | 235 | 100 | 172 | 369 |
| **Problem descriptor** ^4^ | |  |  |  |
| Depression | 1705 (47.00%) | 804 (46.02%) | 1481 (45.75%) | 2712 (44.90%) |
| GAD | 323 (8.90%) | 172 (9.85%) | 378 (11.68%) | 520 (8.61%) |
| Other | 262 (7.22%) | 106 (6.07%) | 296 (9.14%) | 759 (12.57%) |
| MADD | 316 (8.71%) | 145 (8.30%) | 269 (8.31%) | 399 (6.61%) |
| Panic/phobia | 206 (5.68%) | 117 (6.70%) | 202 (6.24%) | 478 (7.91%) |
| Adjustment disorder | 265 (7.30%) | 112 (6.41%) | 278 (8.59%) | 665 (11.01%) |
| PTSD | 398 (10.97%) | 195 (11.16%) | 227 (7.01%) | 312 (5.17%) |
| OCD | 153 (4.22%) | 96 (5.50%) | 106 (3.27%) | 195 (3.23%) |
| Missing | 407 | 184 | 300 | 714 |
| **Age (years)** |  |  |  |  |
| Mean (SD) | 38.03 (13.14) | 36.87 (12.95) | 36.61 (13.07) | 37.96 (13.71) |
| Range | 16.00 - 90.00 | 17.00 - 91.00 | 16.00 - 89.00 | 16.00 - 94.00 |
| **Gender** |  |  |  |  |
| Female | 2745 (68.08%) | 1337 (69.35%) | 2440 (69.04%) | 4456 (66.05%) |
| Male | 1287 (31.92%) | 591 (30.65%) | 1094 (30.96%) | 2290 (33.95%) |
| Missing | 3 | 3 | 3 | 8 |
| **Number of sessions** (including baseline assessment) | | |  |  |
| Mean (SD) | 6.86 (2.93) | 8.15 (2.34) | 6.36 (2.43) | 6.64 (2.60) |
| Range | 3.00 - 11.00 | 3.00 - 11.00 | 3.00 - 11.00 | 3.00 - 11.00 |
| **Recovered** ^5^ |  |  |  |  |
| Yes | 40 (1.03%) | 659 (34.92%) | 2414 (69.11%) | 2790 (64.01%) |
| No | 3854 (98.97%) | 1228 (65.08%) | 1079 (30.89%) | 1569 (35.99%) |
| Missing | 141 | 44 | 44 | 2395 |
| **Reason for end of treatment** |  |  |  |  |
| Discharged | 2745 (68.03%) | 1623 (84.05%) | 2940 (83.12%) | 5829 (86.30%) |
| Dropout | 917 (22.73%) | 222 (11.50%) | 480 (13.57%) | 709 (10.50%) |
| Referred | 373 (9.24%) | 86 (4.45%) | 117 (3.31%) | 216 (3.20%) |
| **Service** |  |  |  |  |
| 0 | 1645 (40.77%) | 829 (42.93%) | 1683 (47.58%) | 2870 (42.49%) |
| 1 | 891 (22.08%) | 443 (22.94%) | 759 (21.46%) | 1309 (19.38%) |
| 2 | 780 (19.33%) | 405 (20.97%) | 654 (18.49%) | 1405 (20.80%) |
| 3 | 719 (17.82%) | 254 (13.15%) | 441 (12.47%) | 1170 (17.32%) |

*Note:* Percentages were calculated using the available sample for each variable, after excluding missing values. The "Missing" row represents the number of missing values and was omitted if there was no missing data. ^1^ Case thresholds: PHQ9 ≥10, GAD7 ≥8. ^2^ ‘Non-worker’ included homemaker, carer, retired, student. ^3^ No negative responses were recorded, therefore the absence of any value was taken as a negative response rather than missing. ^4^ GAD = generalised anxiety disorder; PTSD = post-traumatic stress disorder; OCD = obsessive-compulsive disorder; MADD = mixed anxiety and depressive disorder; Panic/phobia = panic disorder, agoraphobia, social phobia, specific phobia; ‘Other’ included somatoform disorder, severe mental illness. ^5^ Calculated for patients who scored above the case threshold on either/both the PHQ9 or GAD7 at the start of treatment and had an observed score for their final session, otherwise coded as missing. Represents whether the patient reached recovery within the 11 sessions modelled; if a patient received more sessions and then recovered, they would appear unrecovered here.

# Supplementary Table 5. Overlap of patients’ most likely class for four-class growth mixture models of depression (PHQ9) and anxiety (GAD7) symptoms

1. Overlap of class membership across outcomes; values are proportions of the total sample

|  | **Moderate-severe plateau** | **Moderate-severe, gradual improvement** | **Moderate-severe, fast improvement** | **Mild, small improvement** |
| --- | --- | --- | --- | --- |
| **Moderate-severe plateau** | 12.0% |  |  |  |
| **Moderate-severe,  gradual improvement** | 7.5% | 6.6% |  |  |
| **Moderate-severe,  fast improvement** | 1.3% | 3.7% | 10.8% |  |
| **Mild, small improvement** | 5.6% | 5.1% | 11.7% | 35.8% |

1. Values are proportions of patients within the depression class (row) who were assigned to the anxiety class (column)

|  | **Anxiety Class** | | | |
| --- | --- | --- | --- | --- |
| **Depression Class** | **Moderate-severe plateau** | **Moderate-severe, gradual improvement** | **Moderate-severe,  fast improvement** | **Mild, small improvement** |
| **Moderate-severe plateau** | 88.8% | 3.9% | 1.3% | 6.0% |
| **Moderate-severe,  gradual improvement** | 39.5% | 37.6% | 11.1% | 11.9% |
| **Moderate-severe,  fast improvement** | 6.6% | 10.6% | 65.7% | 17.2% |
| **Mild, small improvement** | 9.1% | 5.7% | 16.9% | 68.3% |

1. Values are proportions of patients within the anxiety class (row) who were assigned to the depression class (column)

|  | **Depression Class** | | | |
| --- | --- | --- | --- | --- |
| **Anxiety Class** | **Moderate-severe plateau** | **Moderate-severe, gradual improvement** | **Moderate-severe,  fast improvement** | **Mild, small improvement** |
| **Moderate-severe plateau** | 48.4% | 27.9% | 4.3% | 19.3% |
| **Moderate-severe,  gradual improvement** | 4.5% | 55.6% | 14.6% | 25.3% |
| **Moderate-severe,  fast improvement** | 0.8% | 8.9% | 49.6% | 40.7% |
| **Mild, small improvement** | 2.0% | 5.0% | 6.8% | 86.2% |

*Note*: For panel (b) the cells show the percentage of patients within a specific depression class who belong to each anxiety class. Reading along a row therefore indicates the anxiety classes for all patients in that specific depression class. Panel (c) presents the converse information. For example, 7.5% of patients were in the moderate-severe plateau class in one model and moderate-severe, gradual improvement in the other. For patients in the depression gradual improvement class, 39.5% were in the anxiety plateau class but only 4.5% of patients in the anxiety gradual class were in the depression plateau class.

# Supplementary Table 6. Multinomial regression output of four-class growth mixture model of depression symptoms (PHQ9). Reference class: Moderate-severe plateau. Covariate: Service

| **Class** | **Baseline Variable** | **OR** | **Lower CI** | **Upper CI** | ***p*-value** | **Statistic** | **df** |
| --- | --- | --- | --- | --- | --- | --- | --- |
| Moderate-severe, gradual improvement | (Intercept) | 2.04 | 1.49 | 2.79 | 0.00 | 4.46 | 11799.03 |
| Moderate-severe, gradual improvement | Anxiety symptoms (GAD7) | 1.01 | 1.00 | 1.03 | 0.14 | 1.47 | 6716.22 |
| Moderate-severe, gradual improvement | Functional impairment score (WSAS) | 1.00 | 0.99 | 1.01 | 0.79 | -0.27 | 334.61 |
| Moderate-severe, gradual improvement | Psychotropic medication (Prescribed) | 0.90 | 0.80 | 1.02 | 0.10 | -1.66 | 3287.08 |
| Moderate-severe, gradual improvement | Employment status (Unemployed) | 0.68 | 0.60 | 0.78 | 0.00 | -5.64 | 11374.02 |
| Moderate-severe, gradual improvement | Employment status  (Non-worker) | 0.84 | 0.70 | 1.00 | 0.05 | -1.95 | 6152.48 |
| Moderate-severe, gradual improvement | Disability (Yes) | 0.79 | 0.67 | 0.94 | 0.01 | -2.73 | 16142.33 |
| Moderate-severe, gradual improvement | Problem descriptor (GAD) | 0.82 | 0.65 | 1.05 | 0.11 | -1.59 | 1353.06 |
| Moderate-severe, gradual improvement | Problem descriptor (Other) | 0.89 | 0.70 | 1.14 | 0.37 | -0.89 | 2924.43 |
| Moderate-severe, gradual improvement | Problem descriptor (MADD) | 0.82 | 0.65 | 1.03 | 0.08 | -1.75 | 1773.63 |
| Moderate-severe, gradual improvement | Problem descriptor (Panic/phobia) | 0.79 | 0.60 | 1.04 | 0.10 | -1.67 | 2011.38 |
| Moderate-severe, gradual improvement | Problem descriptor (Adjustment) | 0.77 | 0.60 | 0.98 | 0.04 | -2.09 | 1440.55 |
| Moderate-severe, gradual improvement | Problem descriptor (PTSD) | 0.80 | 0.66 | 0.97 | 0.02 | -2.28 | 3380.85 |
| Moderate-severe, gradual improvement | Problem descriptor (OCD) | 0.79 | 0.56 | 1.10 | 0.17 | -1.39 | 2860.62 |
| Moderate-severe, gradual improvement | Ethnicity (Black) | 0.97 | 0.83 | 1.12 | 0.64 | -0.47 | 5708.46 |
| Moderate-severe, gradual improvement | Ethnicity (Mixed) | 0.93 | 0.74 | 1.16 | 0.50 | -0.67 | 4231.63 |
| Moderate-severe, gradual improvement | Ethnicity (Asian) | 0.95 | 0.77 | 1.17 | 0.63 | -0.49 | 5915.86 |
| Moderate-severe, gradual improvement | Ethnicity (Other) | 0.95 | 0.72 | 1.25 | 0.71 | -0.37 | 7439.76 |
| Moderate-severe, gradual improvement | Age (10 Years) | 1.00 | 0.99 | 1.00 | 0.03 | -2.23 | 15898.82 |
| Moderate-severe, gradual improvement | Gender (Male) | 1.00 | 0.88 | 1.13 | 0.95 | -0.06 | 16031.01 |
| Moderate-severe, fast improvement | (Intercept) | 3.65 | 2.64 | 5.04 | 0.00 | 7.87 | 5720.04 |
| Moderate-severe, fast improvement | Anxiety symptoms (GAD7) | 1.01 | 0.99 | 1.02 | 0.33 | 0.97 | 6725.60 |
| Moderate-severe, fast improvement | Functional impairment score (WSAS) | 0.98 | 0.97 | 0.99 | 0.00 | -3.85 | 141.36 |
| Moderate-severe, fast improvement | Psychotropic medication (Prescribed) | 0.73 | 0.65 | 0.83 | 0.00 | -5.03 | 7485.82 |
| Moderate-severe, fast improvement | Employment status (Unemployed) | 0.48 | 0.42 | 0.55 | 0.00 | -10.34 | 11210.24 |
| Moderate-severe, fast improvement | Employment status  (Non-worker) | 0.76 | 0.64 | 0.92 | 0.00 | -2.93 | 8039.41 |
| Moderate-severe, fast improvement | Disability (Yes) | 0.68 | 0.56 | 0.81 | 0.00 | -4.24 | 15948.96 |
| Moderate-severe, fast improvement | Problem descriptor (GAD) | 0.85 | 0.66 | 1.09 | 0.20 | -1.30 | 936.45 |
| Moderate-severe, fast improvement | Problem descriptor (Other) | 0.99 | 0.77 | 1.26 | 0.90 | -0.12 | 2352.42 |
| Moderate-severe, fast improvement | Problem descriptor (MADD) | 0.97 | 0.76 | 1.23 | 0.77 | -0.29 | 677.29 |
| Moderate-severe, fast improvement | Problem descriptor (Panic/phobia) | 0.78 | 0.58 | 1.06 | 0.11 | -1.60 | 614.04 |
| Moderate-severe, fast improvement | Problem descriptor (Adjustment) | 0.99 | 0.78 | 1.26 | 0.94 | -0.08 | 1537.83 |
| Moderate-severe, fast improvement | Problem descriptor (PTSD) | 0.56 | 0.45 | 0.70 | 0.00 | -5.16 | 1020.18 |
| Moderate-severe, fast improvement | Problem descriptor (OCD) | 0.52 | 0.35 | 0.75 | 0.00 | -3.42 | 2213.52 |
| Moderate-severe, fast improvement | Ethnicity (Black) | 1.18 | 1.02 | 1.37 | 0.03 | 2.19 | 11449.43 |
| Moderate-severe, fast improvement | Ethnicity (Mixed) | 1.01 | 0.80 | 1.26 | 0.94 | 0.07 | 5017.67 |
| Moderate-severe, fast improvement | Ethnicity (Asian) | 0.73 | 0.57 | 0.92 | 0.01 | -2.68 | 3640.97 |
| Moderate-severe, fast improvement | Ethnicity (Other) | 0.73 | 0.53 | 0.99 | 0.04 | -2.04 | 2759.41 |
| Moderate-severe, fast improvement | Age (10 Years) | 1.00 | 0.99 | 1.00 | 0.43 | -0.79 | 14694.27 |
| Moderate-severe, fast improvement | Gender (Male) | 1.05 | 0.92 | 1.19 | 0.49 | 0.70 | 15881.72 |
| Mild, small improvement | (Intercept) | 491.73 | 364.65 | 663.09 | 0.00 | 40.64 | 3594.48 |
| Mild, small improvement | Anxiety symptoms (GAD7) | 0.79 | 0.77 | 0.80 | 0.00 | -33.74 | 4554.31 |
| Mild, small improvement | Functional impairment score (WSAS) | 0.95 | 0.94 | 0.95 | 0.00 | -13.68 | 218.93 |
| Mild, small improvement | Psychotropic medication (Prescribed) | 0.51 | 0.45 | 0.57 | 0.00 | -11.39 | 3767.01 |
| Mild, small improvement | Employment status (Unemployed) | 0.42 | 0.37 | 0.48 | 0.00 | -12.44 | 4064.23 |
| Mild, small improvement | Employment status  (Non-worker) | 0.70 | 0.59 | 0.83 | 0.00 | -4.21 | 4931.36 |
| Mild, small improvement | Disability (Yes) | 0.65 | 0.55 | 0.77 | 0.00 | -4.86 | 12042.08 |
| Mild, small improvement | Problem descriptor (GAD) | 2.48 | 1.99 | 3.10 | 0.00 | 8.05 | 736.47 |
| Mild, small improvement | Problem descriptor (Other) | 1.88 | 1.49 | 2.36 | 0.00 | 5.40 | 1700.28 |
| Mild, small improvement | Problem descriptor (MADD) | 1.23 | 0.98 | 1.56 | 0.08 | 1.75 | 426.93 |
| Mild, small improvement | Problem descriptor (Panic/phobia) | 2.17 | 1.69 | 2.78 | 0.00 | 6.06 | 1254.50 |
| Mild, small improvement | Problem descriptor (Adjustment) | 1.75 | 1.40 | 2.18 | 0.00 | 4.89 | 1319.78 |
| Mild, small improvement | Problem descriptor (PTSD) | 0.86 | 0.70 | 1.05 | 0.14 | -1.50 | 1639.19 |
| Mild, small improvement | Problem descriptor (OCD) | 1.92 | 1.40 | 2.63 | 0.00 | 4.03 | 1184.50 |
| Mild, small improvement | Ethnicity (Black) | 0.71 | 0.61 | 0.82 | 0.00 | -4.53 | 2532.44 |
| Mild, small improvement | Ethnicity (Mixed) | 0.77 | 0.62 | 0.96 | 0.02 | -2.29 | 1619.70 |
| Mild, small improvement | Ethnicity (Asian) | 0.66 | 0.53 | 0.82 | 0.00 | -3.68 | 2032.37 |
| Mild, small improvement | Ethnicity (Other) | 0.61 | 0.45 | 0.81 | 0.00 | -3.39 | 1494.36 |
| Mild, small improvement | Age (10 Years) | 0.99 | 0.99 | 1.00 | 0.00 | -2.92 | 11068.22 |
| Mild, small improvement | Gender (Male) | 0.92 | 0.81 | 1.03 | 0.14 | -1.47 | 10137.76 |

# Supplementary Table 7. Multinomial regression output of four-class growth mixture model of anxiety symptoms (GAD7). Reference class: Moderate-severe plateau. Covariate: Service

| **Class** | **Baseline Variable** | **OR** | **Lower CI** | **Upper CI** | ***p*-value** | **Statistic** | **df** |
| --- | --- | --- | --- | --- | --- | --- | --- |
| Moderate-severe, gradual improvement | (Intercept) | 0.81 | 0.61 | 1.09 | 0.16 | -1.41 | 3800.39 |
| Moderate-severe, gradual improvement | Depression symptoms (PHQ9) | 1.00 | 0.99 | 1.01 | 0.78 | -0.29 | 2604.62 |
| Moderate-severe, gradual improvement | Functional impairment score (WSAS) | 1.00 | 0.99 | 1.00 | 0.26 | -1.13 | 156.60 |
| Moderate-severe, gradual improvement | Psychotropic medication (Prescribed) | 0.84 | 0.75 | 0.94 | 0.00 | -2.93 | 11016.78 |
| Moderate-severe, gradual improvement | Employment status (Unemployed) | 0.63 | 0.55 | 0.72 | 0.00 | -6.59 | 9483.99 |
| Moderate-severe, gradual improvement | Employment status  (Non-worker) | 0.83 | 0.70 | 0.98 | 0.03 | -2.16 | 4734.69 |
| Moderate-severe, gradual improvement | Disability (Yes) | 0.79 | 0.66 | 0.94 | 0.01 | -2.58 | 16015.31 |
| Moderate-severe, gradual improvement | Problem descriptor (GAD) | 1.03 | 0.83 | 1.27 | 0.79 | 0.26 | 1614.51 |
| Moderate-severe, gradual improvement | Problem descriptor (Other) | 0.83 | 0.65 | 1.06 | 0.13 | -1.50 | 4721.10 |
| Moderate-severe, gradual improvement | Problem descriptor (MADD) | 0.96 | 0.77 | 1.19 | 0.72 | -0.36 | 3979.52 |
| Moderate-severe, gradual improvement | Problem descriptor (Panic/phobia) | 1.17 | 0.92 | 1.50 | 0.20 | 1.27 | 2513.73 |
| Moderate-severe, gradual improvement | Problem descriptor (Adjustment) | 0.90 | 0.70 | 1.15 | 0.38 | -0.87 | 1287.93 |
| Moderate-severe, gradual improvement | Problem descriptor (PTSD) | 1.10 | 0.91 | 1.34 | 0.32 | 0.99 | 2268.46 |
| Moderate-severe, gradual improvement | Problem descriptor (OCD) | 1.17 | 0.88 | 1.56 | 0.27 | 1.10 | 1707.62 |
| Moderate-severe, gradual improvement | Ethnicity (Black) | 0.84 | 0.72 | 0.97 | 0.02 | -2.31 | 2695.56 |
| Moderate-severe, gradual improvement | Ethnicity (Mixed) | 0.97 | 0.78 | 1.20 | 0.75 | -0.32 | 7478.47 |
| Moderate-severe, gradual improvement | Ethnicity (Asian) | 0.94 | 0.75 | 1.16 | 0.54 | -0.62 | 4218.63 |
| Moderate-severe, gradual improvement | Ethnicity (Other) | 0.91 | 0.69 | 1.21 | 0.52 | -0.64 | 3134.36 |
| Moderate-severe, gradual improvement | Age (10 Years) | 1.00 | 1.00 | 1.00 | 0.93 | -0.09 | 15735.07 |
| Moderate-severe, gradual improvement | Gender (Male) | 0.99 | 0.88 | 1.11 | 0.84 | -0.21 | 16066.71 |
| Moderate-severe, fast improvement | (Intercept) | 4.11 | 3.23 | 5.22 | 0.00 | 11.54 | 8323.12 |
| Moderate-severe, fast improvement | Depression symptoms (PHQ9) | 0.96 | 0.95 | 0.97 | 0.00 | -6.82 | 2011.34 |
| Moderate-severe, fast improvement | Functional impairment score (WSAS) | 0.98 | 0.98 | 0.99 | 0.00 | -4.50 | 219.56 |
| Moderate-severe, fast improvement | Psychotropic medication (Prescribed) | 0.77 | 0.70 | 0.86 | 0.00 | -4.95 | 4538.06 |
| Moderate-severe, fast improvement | Employment status (Unemployed) | 0.52 | 0.46 | 0.59 | 0.00 | -10.67 | 7746.26 |
| Moderate-severe, fast improvement | Employment status  (Non-worker) | 0.82 | 0.71 | 0.94 | 0.01 | -2.79 | 9144.07 |
| Moderate-severe, fast improvement | Disability (Yes) | 0.73 | 0.62 | 0.86 | 0.00 | -3.86 | 15889.72 |
| Moderate-severe, fast improvement | Problem descriptor (GAD) | 1.06 | 0.89 | 1.26 | 0.50 | 0.68 | 1946.42 |
| Moderate-severe, fast improvement | Problem descriptor (Other) | 1.10 | 0.91 | 1.32 | 0.34 | 0.96 | 5748.92 |
| Moderate-severe, fast improvement | Problem descriptor (MADD) | 1.01 | 0.84 | 1.21 | 0.94 | 0.08 | 2248.86 |
| Moderate-severe, fast improvement | Problem descriptor (Panic/phobia) | 1.00 | 0.81 | 1.25 | 0.97 | 0.04 | 1439.58 |
| Moderate-severe, fast improvement | Problem descriptor (Adjustment) | 1.10 | 0.91 | 1.33 | 0.34 | 0.97 | 1512.85 |
| Moderate-severe, fast improvement | Problem descriptor (PTSD) | 0.73 | 0.61 | 0.88 | 0.00 | -3.40 | 3169.23 |
| Moderate-severe, fast improvement | Problem descriptor (OCD) | 0.61 | 0.47 | 0.80 | 0.00 | -3.55 | 2713.54 |
| Moderate-severe, fast improvement | Ethnicity (Black) | 1.08 | 0.95 | 1.22 | 0.24 | 1.18 | 3553.27 |
| Moderate-severe, fast improvement | Ethnicity (Mixed) | 1.09 | 0.91 | 1.31 | 0.36 | 0.92 | 8158.66 |
| Moderate-severe, fast improvement | Ethnicity (Asian) | 0.80 | 0.66 | 0.97 | 0.02 | -2.29 | 6895.33 |
| Moderate-severe, fast improvement | Ethnicity (Other) | 0.79 | 0.61 | 1.02 | 0.07 | -1.82 | 2938.88 |
| Moderate-severe, fast improvement | Age (10 Years) | 1.00 | 1.00 | 1.00 | 0.37 | -0.90 | 15426.85 |
| Moderate-severe, fast improvement | Gender (Male) | 1.07 | 0.97 | 1.19 | 0.19 | 1.32 | 15623.72 |
| Mild, small improvement | (Intercept) | 166.00 | 129.61 | 212.62 | 0.00 | 40.50 | 2622.32 |
| Mild, small improvement | Depression symptoms (PHQ9) | 0.76 | 0.75 | 0.77 | 0.00 | -43.90 | 1286.53 |
| Mild, small improvement | Functional impairment score (WSAS) | 0.98 | 0.97 | 0.98 | 0.00 | -6.53 | 189.19 |
| Mild, small improvement | Psychotropic medication (Prescribed) | 0.84 | 0.75 | 0.93 | 0.00 | -3.34 | 4701.64 |
| Mild, small improvement | Employment status (Unemployed) | 0.61 | 0.54 | 0.69 | 0.00 | -7.73 | 7036.86 |
| Mild, small improvement | Employment status  (Non-worker) | 0.84 | 0.73 | 0.97 | 0.02 | -2.33 | 10851.74 |
| Mild, small improvement | Disability (Yes) | 0.90 | 0.77 | 1.06 | 0.21 | -1.25 | 14245.83 |
| Mild, small improvement | Problem descriptor (GAD) | 0.38 | 0.31 | 0.45 | 0.00 | -10.59 | 1468.12 |
| Mild, small improvement | Problem descriptor (Other) | 0.82 | 0.68 | 0.99 | 0.04 | -2.11 | 3399.63 |
| Mild, small improvement | Problem descriptor (MADD) | 0.55 | 0.45 | 0.66 | 0.00 | -6.09 | 2099.24 |
| Mild, small improvement | Problem descriptor (Panic/phobia) | 0.58 | 0.47 | 0.73 | 0.00 | -4.83 | 906.81 |
| Mild, small improvement | Problem descriptor (Adjustment) | 0.74 | 0.61 | 0.89 | 0.00 | -3.21 | 2384.31 |
| Mild, small improvement | Problem descriptor (PTSD) | 0.50 | 0.41 | 0.61 | 0.00 | -6.94 | 3581.63 |
| Mild, small improvement | Problem descriptor (OCD) | 0.23 | 0.17 | 0.30 | 0.00 | -10.52 | 1232.33 |
| Mild, small improvement | Ethnicity (Black) | 0.93 | 0.82 | 1.06 | 0.27 | -1.10 | 2685.58 |
| Mild, small improvement | Ethnicity (Mixed) | 0.99 | 0.82 | 1.20 | 0.91 | -0.11 | 6911.04 |
| Mild, small improvement | Ethnicity (Asian) | 0.73 | 0.59 | 0.89 | 0.00 | -3.10 | 5133.23 |
| Mild, small improvement | Ethnicity (Other) | 0.81 | 0.63 | 1.04 | 0.10 | -1.63 | 7302.90 |
| Mild, small improvement | Age (10 Years) | 1.00 | 1.00 | 1.01 | 0.06 | 1.86 | 11238.86 |
| Mild, small improvement | Gender (Male) | 1.24 | 1.12 | 1.38 | 0.00 | 4.16 | 13977.54 |

#

# Supplementary materials references

[Asparouhov, T., & Muthen, B. (2012). Using Mplus TECH11 and TECH14 to test the number of latent classes. *Mplus Web Notes*, 14(22), 1–17.](http://paperpile.com/b/uZsVZL/g3Yrc)

[Bauer, D. J., & Curran, P. J. (2004). The integration of continuous and discrete latent variable models: potential problems and promising opportunities. *Psychological Methods*, 9(1), 3–29.](http://paperpile.com/b/uZsVZL/TMDdZ)

[Clark, S. L., & Muthén, B. (2009). Relating latent class analysis results to variables not included in the analysis, 1–55.](http://paperpile.com/b/uZsVZL/NIsRn)

[Hu, L., & Bentler, P. M. (1999). Cutoff criteria for fit indexes in covariance structure analysis: Conventional criteria versus new alternatives. *Structural Equation Modeling: A Multidisciplinary Journal*, 6(1), 1–55.](http://paperpile.com/b/uZsVZL/EIB50)

[Jung, T., & Wickrama, K. A. S. (2008). An Introduction to Latent Class Growth Analysis and Growth Mixture Modeling. *Social and Personality Psychology Compass*, 2, 302–317.](http://paperpile.com/b/uZsVZL/rni2C)

[Meyer, J. P., & Morin, A. J. S. (2016). A person-centered approach to commitment research: Theory, research, and methodology: Person-Centered Commitment Research. *Journal of Organizational Behavior*, 37(4), 584–612.](http://paperpile.com/b/uZsVZL/9AfGF)

[Muthén, B. O. (2002). Beyond SEM: General latent variable modeling. *Behaviormetrika*, 29(1), 81–117.](http://paperpile.com/b/uZsVZL/cZKRb)

[Nagin, D. S., & Odgers, C. L. (2010). Group-based trajectory modeling in clinical research. *Annual Review of Clinical Psychology*, 6, 109–138.](http://paperpile.com/b/uZsVZL/XMjCV)

[Nylund, K. L., Asparouhov, T., & Muthén, B. O. (2007). Deciding on the Number of Classes in Latent Class Analysis and Growth Mixture Modeling: A Monte Carlo Simulation Study. *Structural Equation Modeling: A Multidisciplinary Journal*, 14(4), 535–569.](http://paperpile.com/b/uZsVZL/CQxFw)

[Petras, H., & Masyn, K. (2010). General Growth Mixture Analysis with Antecedents and Consequences of Change. In A. R. Piquero & D. Weisburd (Eds.), *Handbook of Quantitative Criminology* (pp. 69–100). Springer, New York, NY.](http://paperpile.com/b/uZsVZL/rKwF7)

[Schwarz, G. (1978). Estimating the Dimension of a Model. *The Annals of Statistics*, 6(2), 461–464. Retrieved 17 March 2021 from](http://paperpile.com/b/uZsVZL/jbY24)

[van de Schoot, R., Sijbrandij, M., Winter, S. D., Depaoli, S., & Vermunt, J. K. (2017). The GRoLTS-Checklist: Guidelines for Reporting on Latent Trajectory Studies. *Structural Equation Modeling: A Multidisciplinary Journal*, 24(3), 451–467.](http://paperpile.com/b/uZsVZL/YWelN)

[Wickrama, K. K., Lee, T. K., O’Neal, C. W., & Lorenz, F. O. (2016). *Higher-Order Growth Curves and Mixture Modeling with Mplus: A Practical Guide*. Routledge.](http://paperpile.com/b/uZsVZL/iLVxT)
